# Characterization of Risk Prediction Models for Acute Kidney Injury: A Systematic Review and Meta-analysis

**DOI:** 10.1001/jamanetworkopen.2023.13359

**Published:** 2023-05-15

**Authors:** Yunlin Feng, Amanda Y. Wang, Min Jun, Lei Pu, Steven D. Weisbord, Rinaldo Bellomo, Daqing Hong, Martin Gallagher

**Affiliations:** 1 Department of Nephrology, Sichuan Provincial People’s Hospital, University of Electronic Science and Technology of China, Chengdu, China; 2 The George Institute for Global Health, University of New South Wales, Sydney, Australia; 3 Concord Clinical School, University of Sydney, Sydney, Australia; 4 The Faculty of Medicine and Health Sciences, Macquarie University, Sydney, Australia; 5 Renal Section, Medicine Service, Veterans Affairs Pittsburgh Healthcare System, Pittsburgh, Pennsylvania; 6 Renal-Electrolyte Division, University of Pittsburgh School of Medicine, Pittsburgh, Pennsylvania; 7 Department of Critical Care, University of Melbourne, Melbourne, Australia; 8 South Western Sydney Clinical School, University of New South Wales, Sydney, Australia

## Abstract

**Importance:**

Despite the expansion of published prediction models for acute kidney injury (AKI), there is little evidence of uptake of these models beyond their local derivation nor data on their association with patient outcomes.

**Objective:**

To systematically review published AKI prediction models across all clinical subsettings.

**Data Sources:**

MEDLINE via PubMed (January 1946 to April 2021) and Embase (January 1947 to April 2021) were searched using medical subject headings and text words related to AKI and prediction models.

**Study Selection:**

All studies that developed a prediction model for AKI, defined as a statistical model with at least 2 predictive variables to estimate future occurrence of AKI, were eligible for inclusion. There was no limitation on study populations or methodological designs.

**Data Extraction and Synthesis:**

Two authors independently searched the literature, screened the studies, and extracted and analyzed the data following the Preferred Reporting Items for Systematic Review and Meta-analyses guideline. The data were pooled using a random-effects model, with subgroups defined by 4 clinical settings. Between-study heterogeneity was explored using multiple methods, and funnel plot analysis was used to identify publication bias.

**Main Outcomes and Measures:**

C statistic was used to measure the discrimination of prediction models.

**Results:**

Of the 6955 studies initially identified through literature searching, 150 studies, with 14.4 million participants, met the inclusion criteria. The study characteristics differed widely in design, population, AKI definition, and model performance assessments. The overall pooled C statistic was 0.80 (95% CI, 0.79-0.81), with pooled C statistics in different clinical subsettings ranging from 0.78 (95% CI, 0.75-0.80) to 0.82 (95% CI, 0.78-0.86). Between-study heterogeneity was high overall and in the different clinical settings (eg, contrast medium–associated AKI: *I*^2^ = 99.9%; *P* < .001), and multiple methods did not identify any clear sources. A high proportion of models had a high risk of bias (126 [84.4%]) according to the Prediction Model Risk Of Bias Assessment Tool.

**Conclusions and Relevance:**

In this study, the discrimination of the published AKI prediction models was good, reflected by high C statistics; however, the wide variation in the clinical settings, populations, and predictive variables likely drives the highly heterogenous findings that limit clinical utility. Standardized procedures for development and validation of prediction models are urgently needed.

## Key Points

**Question:**

What are the nature and performance of the many published acute kidney injury (AKI) prediction models across major clinical subsettings, and what is their clinical utility?

**Findings:**

This systematic review and meta-analysis of 150 reported AKI prediction models, with 14.4 million participants, found high variability in study designs, populations examined, AKI definitions used, and model assessments, with most studies deemed at high risk of bias. While the overall discrimination of these models appeared high, there was high between-study heterogeneity that could not be ascribed to any particular variable other than geographical region.

**Meaning:**

In this study, the discrimination of published AKI prediction models appeared good, but the wide variation in the clinical settings, populations, and predictive variables, along with high statistical heterogeneity, limit their clinical utility.

## Teaser

This systematic review and meta-analysis evaluates published acute kidney injury prediction models across all clinical subsettings.

## Introduction

Acute kidney injury (AKI), an abrupt decrease in kidney function encompassing both structural injury and functional impairment, is a growing global health burden that is strongly associated with increased mortality and morbidity.^1,2^ However, for several forms of AKI, there are no effective treatments, which underscores the critical importance of prevention. The cornerstone of prevention for AKI is the identification of patients at high risk for this condition, which has fostered the development of a multitude of models to predict AKI.

The profusion of AKI prediction models published in the last decade is illustrated by a number of systematic reviews in more recent years.^3,4,5,6,7^ These reviews have tended to focus on specific clinical subsettings of patients at risk of AKI, such as after exposure to contrast media,^7^ in general hospital populations,^4^ following noncardiac^3^ or cardiac^5^ surgery, or other specific clinical situations.^6^ Most of these systematic reviews have not proceeded to meta-analyze their findings, primarily due to the heterogeneity of the studies, but they have highlighted a number of limitations in the literature, including variations in performance measures, the use of nonstandardized definitions of AKI, and inconsistent approaches to risk predictor collection.^3,4,5,6,7,8^

Despite this expansion in the available literature, there is little evidence of the uptake of these models beyond their local derivation nor their association with patient outcomes. Understanding the entire AKI risk prediction literature, beyond the specific clinical subsettings of high AKI risk, offers the potential to obtain more general lessons for AKI prevention that, if addressed, may increase the clinical impact of AKI prediction models. Our objective was to systemically review and meta-analyze all published AKI prediction models across all clinical subsettings to define the nature and scope of work in this field and to explore the extent to which these features of the literature may impact the generalizability and uptake of models into clinical care.

## Methods

### Search Strategy and Selection Criteria

We performed a systematic review and meta-analysis of the literature according to the Preferred Reporting Items for Systematic Reviews and Meta-Analyses (PRISMA) statement.^9^ The protocol has been registered on PROSPERO (CRD42021254949). Eligible studies were identified by searching MEDLINE via PubMed (January 1, 1946, to April 8, 2021) and Embase (January 1, 1947, to April 8, 2021) using medical subject headings and text words related to AKI and prediction models (eTable 1 in Supplement 1). All studies that developed a prediction model for AKI (defined as a statistical model with at least 2 risk factors to estimate the future occurrence of AKI), irrespective of the studied population, were eligible for inclusion. Reference lists from included articles were also manually screened to identify any other relevant studies.

### Data Extraction and Quality Assessment

Two authors (Y.F. and L.P.) independently performed the literature search, data extraction, and quality assessment. Published reports were obtained for each eligible study and relevant data were extracted using a standardized data extraction form. Any disagreements in abstract screening and extracted data were resolved by consensus.

Risk of bias of each study was assessed using Prediction Model Risk of Bias Assessment Tool (PROBAST),^10^ which includes 4 domains and 20 signaling questions. Each domain was rated as high risk, low risk, or unclear. A model was rated as having low overall risk of bias if all 4 domains were evaluated as low risk.

Data were extracted from included articles by 2 reviewers (Y.F. and L.P.) and categorized to 5 types, namely study characteristics, covariate information, modeling method, performance assessments, and validation methods (eTable 2 in Supplement 1). AKI definitions were categorized based on the diagnostic criteria used in individual studies, including the Risk, Injury, Failure, Loss of Kidney Function, and End-Stage Kidney Disease (RIFLE) criteria,^11^ Kidney Disease: Improving Global Outcomes (KDIGO) criteria,^12^ Acute Kidney Injury Network (AKIN)^13^ criteria, and self-defined AKI. The AKI stage (or severity) was also assessed according to the diagnostic criteria used. In publications that used the RIFLE criteria, the stage of AKI was defined as stage 1 if the RIFLE Injury criteria were met and stage 3 if the RIFLE Failure criteria were met. The AKI stage from publications that used self-defined AKI criteria was judged on consensus by 2 authors (Y.F. and L.P.) according to the KDIGO criteria.

Studies that reported more than 1 model and had a clearly concluded preference on the best model were treated as reporting a single model, and the best model was considered. For studies that reported more than 1 model and did not present a preferred model, the model with the lowest C statistic was chosen to represent the corresponding study as the most conservative assessment in the primary assessment, and other models were reserved for potential sensitivity analysis. Therefore, each study was treated as having reported 1 model.

### Statistical Analysis

In this study, the C statistic was chosen to represent the discrimination results. Due to the paucity of calibration data reported in the included studies, assessment of calibration was not able to be undertaken in this study.^14^ A detailed description of the data synthesis is provided in eTable 3 in Supplement 1. Briefly, pooled C statistics derived from a random-effects model were used to evaluate the overall discrimination abilities of all prediction models and models in each clinical setting. A summary receiver operating characteristic (sROC) curve with a 95% CI was also generated to evaluate the model performance from the studies that had the requisite data available.^15^ Fagan diagrams were used to examine model effects on post-test probability.^16^ The *I*^2^ statistic^17^ was used to quantify between-study heterogeneity. *I*^2^ values of 25% or less, greater than 25% to 75%, and greater than 75% were considered as having low, moderate, and high heterogeneity, respectively. Between-study heterogeneity was further explored using subgroup analysis, Baujat plot,^18^ influence analysis, and leave-one-out meta-analysis.^19^ Publication bias was evaluated using contour-enhanced funnel plot analysis.^20^

Data analysis was performed using Microsoft Excel 365 (Microsoft Corp), Stata 14 MP (StataCorp), and R version 4.0.3 (R Project for Statistical Computing). Statistical significance was set at *P* < .05, and all tests were 2-tailed.

## Results

### Basic Characteristics

**Figure 1.**
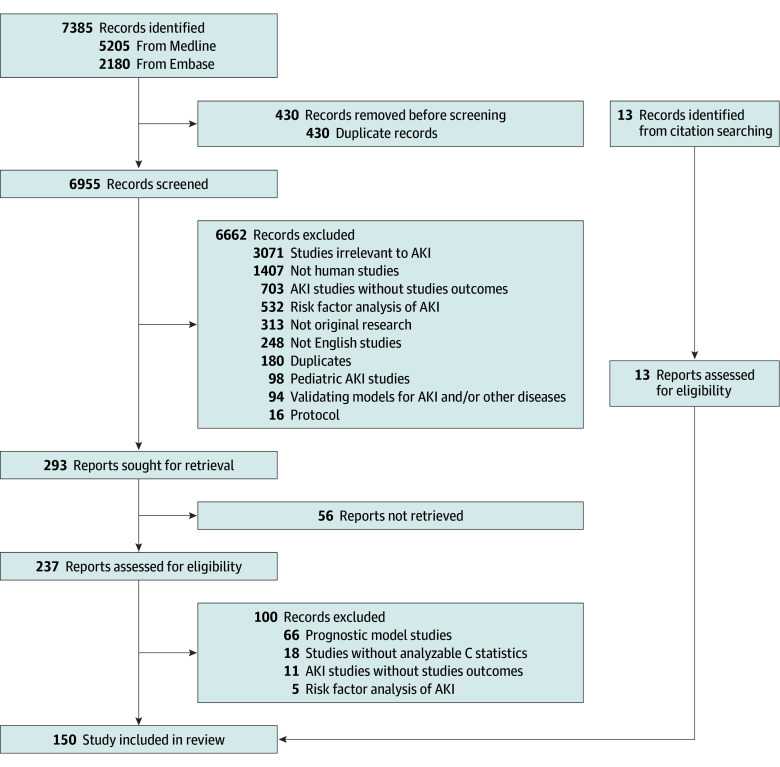
Study Flowchart AKI indicates acute kidney injury.

The study flowchart according to the PRISMA statement is shown in Figure 1. The review included 150 studies (encompassing 14.4 million patients)^21,22,23,24,25,26,27,28,29,30,31,32,33,34,35,36,37,38,39,40,41,42,43,44,45,46,47,48,49,50,51,52,53,54,55,56,57,58,59,60,61,62,63,64,65,66,67,68,69,70,71,72,73,74,75,76,77,78,79,80,81,82,83,84,85,86,87,88,89,90,91,92,93,94,95,96,97,98,99,100,101,102,103,104,105,106,107,108,109,110,111,112,113,114,115,116,117,118,119,120,121,122,123,124,125,126,127,128,129,130,131,132,133,134,135,136,137,138,139,140,141,142,143,144,145,146,147,148,149,150,151,152,153,154,155,156,157,158,159,160,161,162,163,164,165,166,167,168,169,170^ with requisite data to enable sROC curves in 103 studies. The median duration of study data collection was 4 years (range, 3 months to 17 years).

The included studies were highly variable in their study design, study population, AKI definition, and AKI event rate (Table 1; eTable 4 in Supplement 1). Most studies reported prospective data collection (124 [82.7%]).^21,22,24,25,26,28,30,33,34,35,37,38,39,40,41,42,44,45,46,47,48,49,50,51,52,54,55,56,57,59,60,61,62,63,64,65,66,67,68,69,70,71,72,73,74,75,76,77,78,81,82,83,85,86,87,88,89,90,91,92,93,96,97,99,100,101,102,103,104,105,107,108,109,110,111,112,113,115,116,117,118,119,120,121,122,123,124,125,126,127,129,130,131,132,133,134,135,136,137,138,139,141,142,143,144,145,146,147,148,151,153,154,155,157,158,159,160,161,163,164,165,166,167,170^ Based on prespecified clinical scenario subgroups, 29 studies assessed AKI associated with nephrotoxic agents, including 26 studies examining radio-contrast medium associated AKI^23,24,25,33,34,39,43,44,47,52,53,54,55,57,79,80,82,89,115,119,121,128,129,131,132,135^ and 3 studies that reported AKI associated with other nephrotoxins^56,108,126^; 64 studies assessed AKI prediction in the postoperative setting^21,22,26,28,29,30,31,32,36,37,40,41,42,45,46,49,51,58,59,60,63,64,65,66,67,71,72,73,74,75,78,81,86,90,91,92,94,96,97,98,99,101,104,105,107,109,111,112,116,117,118,120,124,127,130,133,134,144,146,161,162,163,164,165^; 21 studies assessed AKI prediction in intensive care units (ICU)^27,84,88,93,95,100,122,123,136,138,140,145,147,148,149,150,155,156,157,168,169^; 35 studies assessed AKI in general hospitalizations^35,38,48,50,61,62,68,69,70,76,77,83,85,87,102,103,106,110,113,114,125,137,139,141,142,151,152,153,154,158,159,160,166,167,170^; and 1 assessed AKI in the outpatient setting.^143^ The most common region where the studies had been conducted was the Asia-Pacific region, accounting for 40.0% of the included studies (60 studies)^27,34,37,39,42,43,44,46,50,51,54,55,57,64,65,66,67,71,72,74,76,77,78,79,80,81,82,83,88,94,101,102,114,115,117,118,120,122,126,128,129,131,133,134,135,138,144,145,146,149,150,160,161,162,163,165,167,168,169,170^, followed by North America (37.3% [56 studies])^21,22,24,30,32,33,35,38,41,45,47,48,49,52,53,60,61,62,63,68,69,75,84,85,86,87,89,90,93,98,100,104,108,110,113,116,119,124,125,127,130,132,136,137,140,141,142,143,147,148,151,153,154,157,159,166^ and Europe (20.0% [20 studies]).^25,26,28,29,31,36,56,58,59,70,73,91,92,95,96,99,103,105,106,107,109,111,112,121,123,139,155,156,158,164^

**Table 1.** Characteristics of Included Prediction Models^a^

Characteristic	Studies, No. (%)				
	All studies (N = 150)	AKI		AKI in ICU (n = 21)	AKI in general hospitalization (n = 35)
		CM associated (n = 26)	Postoperative (n = 64)		
Study design					
Retrospective	124 (82.7)	20 (76.9)	56 (87.5)	12 (57.1)	32 (91.4)
Prospective	26 (17.3)	6 (23.1)	8 (12.5)	9 (42.9)	3 (8.6)
Population size, median (range)	1598 (45-6 390 410)	982 (192-947 091)	2131 (71-4 449 524)	749 (94-151 098)	2395 (45-6 390 410)
Observed AKI Incidence					
≤10%	55 (36. 7)	15 (57.7)	22 (34.4)	3 (14.3)	14 (40.0)
>10% to ≤20%	43 (28. 7)	10 (38.5)	15 (23.4)	7 (33.3)	9 (25.7)
>20%	52 (34. 7)	1 (3.8)	27 (42.2)	11 (52.4)	12 (34.3)
AKI Definition					
KDIGO	76 (50. 7)	6 (23.1)	26 (40.6)	18 (85.7)	24 (68.6)
AKIN	17 (11.3)	1 (3.8)	10 (15.6)	1 (4.8)	5 (14.3)
RIFLE	9 (6.0)	1 (3.8)	5 (7.8)	0 (0)	3 (8.6)
Others	48 (32.0)	18 (69.2)	23 (35.9)	2 (9.5)	3 (8.6)
Predicted time window					
<48 h	52 (34.7)	8 (30.8)	27 (42.2)	5 (23.8)	12 (34.3)
48 h to 7 d	86 (57.3)	16 (61.5)	32 (50.0)	15 (71.4)	21 (60.0)
>7 d	10 (6.7)	2 (7.7)	4 (6.3)	1 (4.8)	1 (2.9)
Not specified	2 (1.3)	0 (0)	1 (1.6)	0 (0)	1 (2.9)
No. of predictive variables					
≤15	115 (76. 7)	24 (92.3)	49 (76.6)	16 (76.2)	23 (65.7)
>15	35 (23.3)	2 (7.7)	15 (23.4)	5 (23.8)	12 (34.3)
Modeling algorithms					
Logistic regression	109 (72. 7)	22 (84.6)	46 (71.9)	16 (76.2)	22 (62.9)
Machine learning algorithms	41 (27.3)	4 (15.4)	18 (28.1)	5 (23.8)	13 (37.1)
Reported discrimination measures					
C statistics	140 (93.3)	23 (88.5)	60 (93.8)	20 (95.2)	33 (94.3)
Others	21 (14.0)	2 (7.7)	6 (9.4)	6 (28.6)	7 (20.0)
Not reported	9 (6.0)	3 (11.5)	3 (4.7)	1 (4.8)	2 (5.7)
Reported calibration measures					
Calibration plot	31 (20.7)	4 (15.4)	18 (28.1)	4 (19.0)	5 (14.3)
H-L test	56 (37.3)	12 (46.2)	25 (39.1)	8 (38.1)	9 (25.7)
Others	20 (13.3)	4 (15.4)	7 (10.9)	3 (14.3)	6 (17.1)
Not reported	36 (24.0)	6 (23.1)	1 (1.6)	8 (38.1)	19 (54.3)
Internal validations					
Yes	95 (63.3)	20 (76.9)	36 (56.3)	13 (61.9)	24 (68.6)
No	55 (36.7)	6 (23.1)	28 (43.8)	8 (38.1)	11 (31.4)
External validations					
Yes	36 (24.0)	5 (19.2)	19 (29.7)	3 (14.3)	7 (20.0)
No	114 (76.0)	21 (80.8)	45 (70.3)	18 (85.7)	28 (80.0)
Risk of bias					
High	126 (84.0)	23 (88.5)	53 (82.8)	19 (90.5)	29 (82.9)
Low	24 (16.0)	3 (11.5)	11 (17.2)	2 (9.5)	6 (17.1)

Abbreviations: AKI, acute kidney injury; AKIN, Acute Kidney Injury Network; CM, contrast medium; H-L test, Hosmer-Lemeshow test; ICU, intensive care units; KDIGO, Kidney Disease: Improving Global Outcomes; RIFLE, Risk, Injury and Failure, Loss and End-Stage Kidney Disease.

^a^ Four studies were excluded from analysis based on clinical subsettings, including 3 studies of AKI associated with toxic agents other than contrast medium and 1 study of AKI in the primary care clinic setting.

The AKI definitions varied across the studies, with most being the KDIGO criteria (76 [50.7%]),^21,22,27,28,29,31,35,36,37,38,39,40,46,48,50,51,52,53,56,59,67,68,69,71,74,75,76,77,78,80,81,84,85,88,100,102,103,105,106,110,113,115,117,118,120,122,123,125,126,127,133,136,138,139,140,144,145,148,149,150,152,153,154,155,156,157,158,159,160,161,162,163,166,167,168,169^ followed by the AKIN criteria (17 [11.3%])^42,49,60,61,62,65,72,73,91,94,109,114,119,137,142,146,147^ and the RIFLE criteria (9 [6.0%]).^66,70,86,87,96,111,112,132,141^ Nearly a quarter of the studies (35 [23.3%]) defined AKI using criteria specific to their particular studies.^23,24,25,26,30,32,33,34,43,47,55,57,58,79,82,83,89,92,93,97,98,101,104,107,108,121,128,129,130,131,135,151,164,165,170^ A large majority of the studies defined AKI using the least severe stage of the disease (stage 1, 118 of 146 [80.8%]).^21,22,23,24,25,26,27,28,29,31,33,34,35,36,37,39,40,42,43,46,47,48,49,50,51,52,53,54,55,56,58,60,61,62,65,66,67,68,69,70,71,72,73,74,75,76,77,78,79,80,81,82,83,85,86,87,89,91,94,96,97,100,102,103,106,108,110,111,112,113,114,115,117,118,119,120,121,122,123,125,126,127,128,129,130,131,132,133,135,136,137,138,139,141,142,145,148,149,150,151,152,153,154,155,156,157,158,159,160,161,162,163,164,166,167,168,169,170^ The median (IQR) values for AKI event rate and sample size for model development were 13.4% (7.14%-27.9%) and 1598 (478-16 668), respectively.

### Model Assessment Results

The included prediction models varied in terms of the number and nature of predictive variables, modeling algorithms, discrimination measures, calibration measures, and use of both internal and external validation (eTable 5 in Supplement 1). Most studies (110 [73.3%]) used regression algorithms,^23,24,25,26,27,28,29,31,32,34,36,37,38,39,40,41,42,43,44,45,46,47,48,51,53,54,55,56,57,58,59,60,63,64,65,66,68,70,73,78,79,80,81,82,83,84,86,87,88,89,90,91,95,96,97,98,99,102,104,106,107,108,109,110,111,112,114,116,117,118,119,121,123,124,126,128,129,132,133,135,136,137,138,139,140,141,142,143,144,145,146,148,149,150,151,152,153,154,155,156,158,160,161,162,164,165,166,168,169,170^ while the others used various machine learning algorithms. Among the 140 studies (93.3%) that reported predictive variables,^21,22,23,24,25,26,27,28,29,30,31,32,33,34,36,37,38,39,40,41,42,43,44,45,46,47,48,49,50,51,52,53,54,55,56,57,58,59,60,61,63,64,65,66,67,68,69,70,71,72,73,76,77,78,79,80,81,82,83,84,86,87,88,89,90,92,94,95,96,97,98,99,101,102,103,104,105,106,107,108,109,110,111,112,113,114,115,116,117,118,119,120,121,123,124,126,127,128,129,130,131,132,133,134,135,136,137,138,139,140,141,142,143,144,145,146,147,148,149,150,151,152,153,154,155,156,157,158,159,160,161,162,163,164,165,166,167,168,169,170^ the median (IQR) number of predictive variables was 7 (4-13). Predictive variables in each model also varied, ranging from demographic characteristics (eg, age, sex) to clinical variables (eg, laboratory values, vital signs, imaging results, and drugs) and biomarkers not yet routinely used in clinic practice.

The availability of predictive variables at the time of model prediction was rated as yes or probably yes and no or probably no in 116 studies (77.3%)^21,22,23,24,25,26,27,28,29,30,31,32,33,34,35,36,37,38,39,40,41,42,43,44,45,46,47,48,49,50,51,52,53,54,55,56,57,58,59,60,61,62,63,64,65,66,67,68,69,70,71,72,73,74,75,76,77,78,79,80,81,82,83,84,85,86,87,88,89,90,91,92,93,94,95,96,97,98,99,100,101,102,103,104,105,106,107,108,109,110,111,112,113,114,115,116,117,118,119,120,121,122,123,124,125,126,127,128,129,130,131,132,133,134,135,136^ and 34 studies (22.7%),^137,138,139,140,141,142,143,144,145,146,147,148,149,150,151,152,153,154,155,156,157,158,159,160,161,162,163,164,165,166,167,168,169,170^ respectively. The possibility of overlap between the window of outcome prediction and the starting point of the prediction in all included models was rated as no or probably no and yes or probably yes in 118 studies (78.7%)^21,22,23,24,25,26,27,28,29,30,31,32,33,34,35,36,37,38,39,40,41,42,43,44,46,47,48,49,50,51,52,53,54,55,56,57,58,59,60,61,62,63,64,65,66,67,68,69,70,71,72,73,74,75,76,77,78,79,80,81,82,83,84,85,86,87,88,89,90,91,92,93,94,95,96,97,98,99,100,101,102,103,104,105,106,107,108,109,110,111,112,113,114,115,116,117,118,119,120,121,122,123,124,125,126,127,128,129,130,131,132,133,134,135,136,137,142,153^ and 32 studies (21.3%),^45,138,139,140,141,143,144,145,146,147,148,149,150,151,152,154,155,156,157,158,159,160,161,162,163,164,165,166,167,168,169,170^ respectively. The most frequently reported measures in performance assessments were the C statistic for discrimination and Hosmer-Lemeshow test for calibration. Among the 150 studies, 95 (63.3%) were internally validated,^21,22,23,26,28,29,30,33,34,35,36,37,38,39,41,42,43,44,45,46,47,50,51,54,56,57,59,63,66,68,69,70,71,72,74,75,76,79,80,81,82,83,84,86,87,88,89,92,96,100,101,102,103,105,110,111,112,113,114,115,116,117,118,119,120,121,122,125,126,127,128,129,131,132,133,134,137,139,140,147,148,149,150,151,153,154,155,157,158,160,163,167,168,169,170^ whereas 36 studies (24.0%) were externally validated.^24,31,33,48,55,58,67,78,83,84,89,90,92,94,97,98,99,104,107,108,110,113,121,124,126,135,139,140,144,146,152,161,162,163,166,169^

### Pooled Performance Assessment

The pooled C statistics across all included studies was 0.80 (95% CI, 0.79-0.81) (Table 2). The pooled C statistics across the different clinical settings of contrast medium associated AKI, postoperative AKI, AKI in the ICU, and AKI in general hospitalizations were in the range of 0.78 (95% CI, 0.75-0.80) to 0.82 (95% CI, 0.78-0.86), with minimal differences among these 4 subgroups (Table 2). Graphical illustrations of the pooled analyses of C statistics are presented as forest plots (eFigures 1-4 in Supplement 1) and Drapery plots (eFigures 5-8 in Supplement 1) for each subgroup, illustrating the narrow confidence intervals of estimates from most studies and the heterogeneity of results.

**Table 2.** Pooled Analysis of C Statistics From All Studies and by Clinical Settings

Setting	C statistic	95% CI	95% PI
All studies (N = 150)	0.80	0.79-0.81	0.65-0.96
CM-associated AKI (n = 26)	0.81	0.78-0.85	0.64-0.99
Postoperative AKI (n = 64)	0.81	0.79-0.83	0.66-0.96
AKI in ICU (n = 21)	0.82	0.78-0.86	0.64-1.00
AKI in general hospitalizations (n = 35)	0.78	0.75-0.80	0.61-0.94

Abbreviations: AKI, acute kidney injury; CM, contrast medium; ICU, intensive care unit; PI, prediction interval.

**Figure 2.**
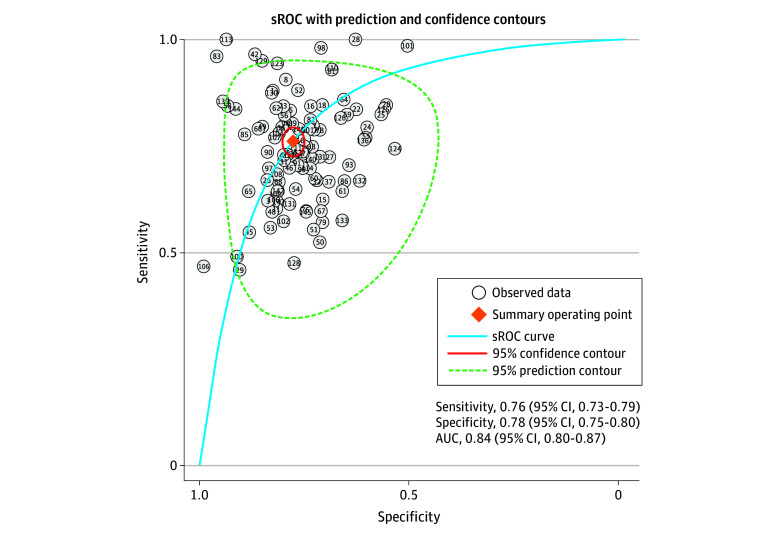
Summary Receiver Operating Characteristic (sROC) Curves of C Statistics of All Studies The pooled area under the curve (AUC) values provided with sROC curves were different from the pooled C statistics due to differences in available data sets.

sROC curves for all studies (Figure 2) and each individual clinical setting (eFigure 9-12 in Supplement 1) illustrate the variations in sensitivity and specificity of models in predicting AKI, with the broad 95% prediction contours (or regions) reflecting the range within which the true sensitivity and specificity lie for such models. The pooled sensitivities and specificities were in the range of 0.75 to 0.79 and 0.75 to 0.82, respectively (eTable 6 in Supplement 1). Fagan nomograms were used to indicate the impact of positive and negative model results on the posttest probability of developing AKI (eFigure 13 in Supplement 1 for all studies and eFigures 14-17 in Supplement 1 for each setting), assuming that the risk of developing AKI across the study populations is 20%.

High between-study heterogeneity reflected by the high *I*^2^ values seen overall and in all 4 subgroups: contrast medium–associated AKI (*I*^2^ = 99.9%; *P* < .001), postoperative AKI (*I*^2^ = 99.4%; *P* < .001), AKI in the ICU (*I*^2^ = 99.6%; *P* < .001), and AKI in general hospitalizations (*I*^2^ = 99.1%; *P* < .001). These findings were also supported by wide prediction intervals on the Drapery plots of each subgroup (eFigures 5-8 in Supplement 1).

### Sensitivity Analyses of Pooled C Statistics

**Figure 3.**
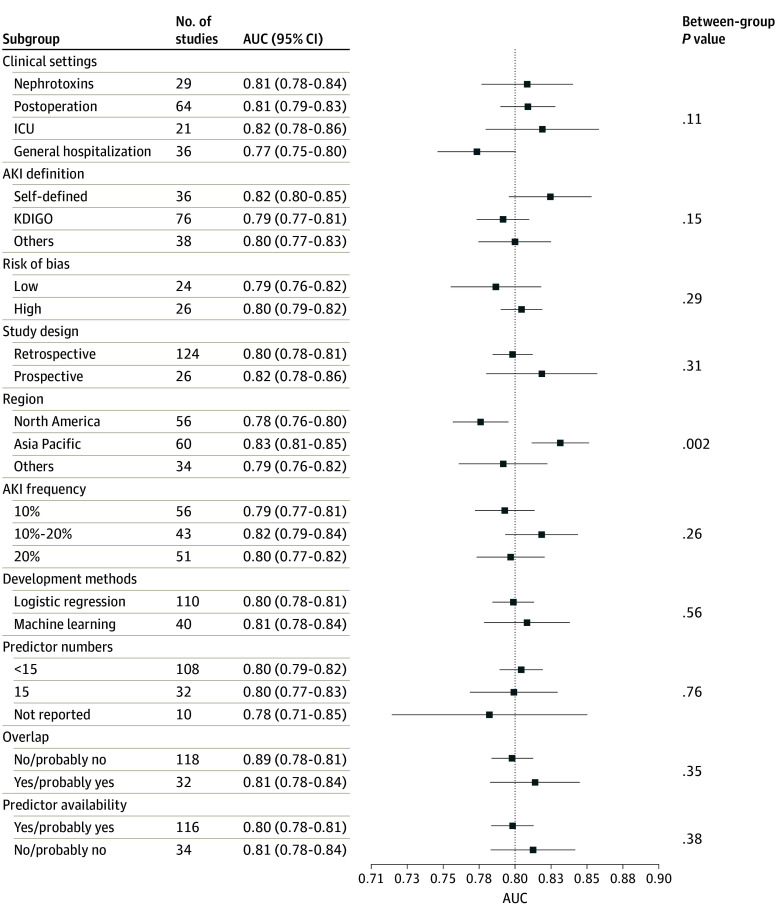
Subgroup Analysis of Pooled C Statistics to Explore Potential Sources for Between-Study Heterogeneities AKI indicates acute kidney injury; AUC, area under the curve; ICU, intensive care unit; KDIGO, Kidney Disease: Improving Global Outcomes.

To further explore potential sources of the high heterogeneity, several sensitivity analyses were conducted. Subgroup analysis of pooled C statistics (Figure 3) and sROC analysis (eTable 7 in Supplement 1) did not identify any discrete sources of the heterogeneity observed between studies, except for study region for pooled C statistics. Metaregression analysis of potential influencing variables against C statistics (eFigure 18 in Supplement 1) and sensitivity analyses using Baujat plots (eFigures 19-22 in Supplement 1) and leave-one-out meta-analysis (eFigures 23-26 in Supplement 1) for each subgroup also failed to identify any significant source of such high heterogeneities in all studies and each clinical setting. In addition, the reported standard error of the C statistic in studies with small sample size tended to be smaller than the corresponding estimated standard error (eFigure 27 in Supplement 1). The differences between reported standard error and estimated standard error decreased with increasing sample size.

### Publication Bias Assessment

Publication bias was assessed using funnel plot analysis (eFigures 28-31 in Supplement 1). In all 4 subgroups, regular funnel plot analysis using the Egger test did not indicate the presence of funnel plot asymmetry. These findings were further confirmed by results of contour-enhanced funnel plot analysis. In addition, adjusted funnel plot using the Duval and Tweedie trim-and-fill method also confirmed the symmetric distribution of included publications. Taken together, these results indicated that publication bias was not a source for between-study heterogeneity in any subgroups.

### Risk of Bias Assessment

Risk of bias assessments based on the PROBAST tool indicated that most studies had high risk of bias (126 [84.4%]), including 23 studies (88.5%) of AKI associated with nephrotoxins,^23,24,25,34,39,43,44,47,53,54,55,57,79,80,82,89,119,121,128,129,131,132,135^ 53 studies (82.8%) of postoperative AKI,^21,22,26,31,32,37,40,42,45,49,58,59,60,63,64,65,66,71,72,73,74,75,78,81,86,91,92,94,96,97,99,101,104,107,109,111,112,116,117,118,120,124,127,130,133,134,144,146,161,162,163,164,165^ 19 studies (90.5%) of AKI in the ICU,^27,84,93,95,122,123,136,138,140,145,147,148,149,150,155,156,157,168,169^ and 29 studies (82.9%) of AKI in general hospitalizations^35,48,62,68,70,76,83,87,102,103,106,110,113,114,125,137,139,141,142,151,152,153,154,158,159,160,166,167,170^ (eFigures 32-35 in Supplement 1). In all 4 subgroups, the analysis domain most frequently reported high risk of bias.

## Discussion

This systematic review of the rapidly expanding field of AKI prediction models included 150 studies encompassing 14.4 million participants. Our findings indicate that while predictive models for AKI have good overall discriminative power, the populations studied, methods used, and the estimates derived vary widely across clinical settings and populations such that their clinical utility is seriously limited.

Previous systematic reviews of predictive models have focused on clinical subsets of AKI, and most have not meta-analyzed the data due to the statistical heterogeneity of studies, which is commonly seen when aggregating prediction models.^4,7^ Similarly, we observed that this literature uses widely varying populations, outcome definitions, predictive variables, and approaches to data collection. However, by meta-analyzing our results, most notably in the derivation of summary ROC figures, we illustrated how this variation leads to significant imprecision in the estimates of model sensitivity and specificity, and thereby seriously compromises the applicability and/or external validity.

The variation in all elements of the AKI prediction models examined here is profound, seen in the nature of the clinical populations studied, the timing and nature of the prediction variables, and in the timing and nature of the AKI outcomes. Despite testing many potential sources of heterogeneity, no discrete factor was identified as primarily driving the wide variation seen. These findings were consistent across the totality of studies, and in the 4 clinical subsettings of AKI prediction models, suggesting that despite many publications, the sector appears little closer to robust and generalizable risk prediction tools. The high proportion of study data sets derived retrospectively and not externally validated also points to frequent use of existing data fields that may not be readily available in different clinical settings and further challenges external validation and broader model clinical application. The meta-analysis powerfully illustrates the wide variations in model discrimination and performance but also highlights the incongruity of a number of models with extremely high sensitivity and specificity (and therefore discrimination) that are unlikely to be reflected in their clinical application.

To bring greater structure and clinical utility to this space there needs to be greater consistency around the populations studied and the methods used. We examined 4 common clinical settings for the development of AKI, but the value of work in these areas is debatable. For example, recent studies of radiocontrast-associated AKI^171,172^ have shown AKI rates of approximately 10%; however, only a minority (approximately 1 in 10) of these events represent severe (or stage 3) AKI, where the associations with significant long-term harms are greatest.^173^ Defining clinical phenotypes and settings of patients at high risk for more severe AKI (stage 2 or greater), that are replicable internationally, will be essential to enhancing the clinical utility and impact of any future AKI models.

Consistency of analytical methods is also central to reducing heterogeneity and enhancing the value of AKI models. The variation in methods is best illustrated by the different definitions of AKI used in studies, with approximately one-third of studies not using 1 of the 3 recognized definitions, and the time window for AKI prediction varying from less than 2 days to more than 7 days. Beyond this variation in outcomes is an array of bespoke risk prediction variables that, with machine learning techniques, can number into the hundreds and may lack any formal definitions. Without consistency across the outcomes and prediction variables it is not possible to build on the work of others by externally validating model performance and testing the association of additional variables with the performance of existing models. Should we develop greater consistency in AKI populations and analyses, approaches to validation as suggested by Debray et al^174^ would offer a way forward in model validation and development. Existing guideline collaborations active in this space, such as the KDIGO group or the Acute Disease Quality Initiative, may be best placed to lead the standardization of AKI modeling work.

A strength of our analysis is its comprehensive literature search that illustrates the breadth and nature of AKI prediction models. To our knowledge, it is also the first systematic review in AKI to use summary ROC curves to illustrate the imprecision of summary estimates and to use the PROBAST tool to assess risk of bias, with only 16% of reports rated as being at low risk of bias. To our knowledge, there are no prediction models that have been specifically recommended by any academic guidelines, however some prediction models are used in clinical practice, such as the prediction model for postangiography AKI by Mehran et al^89^ and the AKIpredictor in critically ill patients by Flechet et al.^157^

### Limitations

There are several limitations. First, the pooled analysis only assessed model discrimination, as most models reported this in some form (usually C statistics), with scarce reporting of model calibration precluding its meta-analysis. Second, the high variability in studies, perhaps driven in part by the breadth of our approach, saw us unable to conclude on specific factors that were driving the high between-study heterogeneity of the reported AKI prediction models.

## Conclusions

This comprehensive systematic review and meta-analysis of AKI prediction models highlighted important limitations and challenges in this field, which profoundly limit the clinical utility of this literature in preventing AKI or defining high-risk populations in which preventative treatments can be tested. While no specific factors were found to drive the between-study heterogeneity, the high variability in the study populations, reported outcomes, and predictive variables are limiting the ability of studies to improve the outcomes of patients with AKI. More standardized approaches to the development and validation of prediction models are urgently needed and should cover aspects including study population selection, predictive variables selection, data analysis, performance assessment, and reporting paradigm.

## Notes

Supplement 1. **eTable 1.** Literature Search Strategy **eTable 2.** Details of Extracted Variables **eTable 3.** Details of Data Synthesis **eTable 4.** Details of Basic Characteristics of the Included Prediction Models **eTable 5.** Details of Model Assessment Results of Included Prediction Models **eTable 6.** Pooled C Statistics, Sensitivities, and Specificities From sROC Analysis **eTable 7.** Results of Subgroup Analysis for Potential Significant Sources of High Heterogeneities **eFigure 1.** Forest Plot for Meta-analysis of C Statistics of Contrast Medium Associated AKI Prediction Models **eFigure 2.** Forest Plot for Meta-analysis of C Statistics of Postoperative AKI Prediction Models **eFigure 3.** Forest Plot for Meta-analysis of C Statistics of Prediction Models of AKI in ICU **eFigure 4.** Forest Plot for Meta-analysis of C Statistics of Prediction Models of AKI in General Hospitalization **eFigure 5.** Drapery Plot for Meta-analysis of C Statistics of Contrast Medium Associated AKI Prediction Models **eFigure 6.** Drapery Plot for Meta-analysis of C Statistics of Postoperative AKI Prediction Models **eFigure 7.** Drapery Plot for Meta-analysis of C Statistics of Prediction Models of AKI in ICU **eFigure 8.** Drapery Plot for Meta-analysis of C Statistics of Prediction Models of AKI in General Hospitalization **eFigure 9.** Summary ROC Curves of C Statistics of Contrast Medium Associated AKI Prediction Models **eFigure 10.** Summary ROC Curves of C Statistics of Postoperative AKI Prediction Models **eFigure 11.** Summary ROC Curves of C Statistics of Prediction Models of AKI in ICU **eFigure 12.** Summary ROC Curves of C Statistics of Prediction Models of AKI in General Hospitalization **eFigure 13.** Bayesian Analysis of C Statistics of AKI Prediction Models in All Clinical Settings **eFigure 14.** Bayesian Analysis of C Statistics of Contrast Medium Associated AKI Prediction Models **eFigure 15.** Bayesian Analysis of C Statistics of Postoperative AKI Prediction Models **eFigure 16.** Bayesian Analysis of C Statistics of Prediction Models of AKI in ICU **eFigure 17.** Bayesian Analysis of C Statistics of Prediction Models of AKI in General Hospitalization **eFigure 18.** Metaregression Analysis of Variables That Were Suspected to Be the Sources of Heterogeneities Against C Statistics (Weighted Against Standard Errors of C Statistics) **eFigure 19.** The Baujat Plot for Between-Study Heterogeneity Analysis of Contrast Medium Associated AKI Prediction Models **eFigure 20.** The Baujat Plot for Between-Study Heterogeneity Analysis of Postoperative AKI Prediction Models **eFigure 21.** The Baujat Plot for Between-Study Heterogeneity Analysis of Prediction Models of AKI in ICU **eFigure 22.** The Baujat Plot for Between-Study Heterogeneity Analysis of Prediction Models of AKI in General Hospitalization **eFigure 23.** Influence Analysis for Between-Study Heterogeneity Analysis of Contrast Medium Associated AKI Prediction Models **eFigure 24.** Influence Analysis for Between-Study Heterogeneity Analysis of Postoperative AKI Prediction Models **eFigure 25.** Influence Analysis for Between-Study Heterogeneity Analysis of Prediction Models of AKI in ICU **eFigure 26.** Influence Analysis for Between-Study Heterogeneity Analysis of Prediction Models of AKI in General Hospitalization **eFigure 27.** The Comparisons Between Reported Standard Error and Estimated Error of C Statistics of Each Study in the Whole Population and Each Subgroup **eFigure 28.** Funnel Plot Analysis for Meta-analysis of C Statistics of Contrast Medium Associated AKI Prediction Models **eFigure 29.** Funnel Plot Analysis for Meta-analysis of C Statistics of Postoperative AKI Prediction Models **eFigure 30.** Funnel Plot Analysis for Meta-analysis of C Statistics of Prediction Models of AKI in ICU **eFigure 31.** Funnel Plot Analysis for Meta-analysis of C Statistics of Prediction Models of AKI in General Hospitalization **eFigure 32.** Risk of Bias Assessment Results Using PROBAST Tool for Prediction Models on Contrast Medium Associated AKI **eFigure 33.** Risk of Bias Assessment Results Using PROBAST Tool for Prediction Models on Postoperative AKI **eFigure 34.** Risk of Bias Assessment Results Using PROBAST Tool for Prediction Models on Postoperative AKI **eFigure 35.** Risk of Bias Assessment Results Using PROBAST Tool for Prediction Models of AKI in General Hospitalization
eReferences.

Supplement 2.
Data Sharing Statement
