# Supplementary material for: Characterization of Risk Prediction Models for Acute Kidney Injury: A Systematic Review and Meta-analysis
Source: JAMA Netw Open. 2023 May 15;6(5):e2313359. doi: 10.1001/jamanetworkopen.2023.13359 (PMC12011341; doi:10.1001/jamanetworkopen.2023.13359)
Supplement: Supplement 1. — eTable 1. Literature Search Strategy eTable 2. Details of Extracted Variables eTable 3. Details of Data Synthesis eTable 4. Details of Basic Characteristics of the Included Prediction Models eTable 5. Details of Model Assessment Results of Included Prediction Models eTable 6. Pooled C Statistics, Sensitivities, and Specificities From sROC Analysis eTable 7. Results of Subgroup Analysis for Potential Significant Sources of High Heterogeneities eFigure 1. Forest Plot for Meta-analysis of C Statistics of Contrast Medium Associated AKI Prediction Models eFigure 2. Forest Plot for Meta-analysis of C Statistics of Postoperative AKI Prediction Models eFigure 3. Forest Plot for Meta-analysis of C Statistics of Prediction Models of AKI in ICU eFigure 4. Forest Plot for Meta-analysis of C Statistics of Prediction Models of AKI in General Hospitalization eFigure 5. Drapery Plot for Meta-analysis of C Statistics of Contrast Medium Associated AKI Prediction Models eFigure 6. Drapery Plot for Meta-analysis of C Statistics of Postoperative AKI Prediction Models eFigure 7. Drapery Plot for Meta-analysis of C Statistics of Prediction Models of AKI in ICU eFigure 8. Drapery Plot for Meta-analysis of C Statistics of Prediction Models of AKI in General Hospitalization eFigure 9. Summary ROC Curves of C Statistics of Contrast Medium Associated AKI Prediction Models eFigure 10. Summary ROC Curves of C Statistics of Postoperative AKI Prediction Models eFigure 11. Summary ROC Curves of C Statistics of Prediction Models of AKI in ICU eFigure 12. Summary ROC Curves of C Statistics of Prediction Models of AKI in General Hospitalization eFigure 13. Bayesian Analysis of C Statistics of AKI Prediction Models in All Clinical Settings eFigure 14. Bayesian Analysis of C Statistics of Contrast Medium Associated AKI Prediction Models eFigure 15. Bayesian Analysis of C Statistics of Postoperative AKI Prediction Models eFigure 16. Bayesian Analysis of C Statistics of Prediction Models of AKI in ICU eFi [file jamanetwopen-e2313359-s001.pdf]

Supplemental Online Content

Feng Y, Wang AY, Jun M, et al. Characterization of risk prediction models for acute kidney injury: a systematic review and meta-analysis. *JAMA Netw Open*. 2023;6(5):e2313359. doi:10.1001/jamanetworkopen.2023.13359

eTable 1. Literature Search Strategy
eTable 2. Details of Extracted Variables
eTable 3. Details of Data Synthesis
eTable 4. Details of Basic Characteristics of the Included Prediction Models
eTable 5. Details of Model Assessment Results of Included Prediction Models
eTable 6. Pooled C Statistics, Sensitivities, and Specificities From sROC Analysis
eTable 7. Results of Subgroup Analysis for Potential Significant Sources of High Heterogeneities
eFigure 1. Forest Plot for Meta-analysis of C Statistics of Contrast Medium Associated AKI Prediction Models
eFigure 2. Forest Plot for Meta-analysis of C Statistics of Postoperative AKI Prediction Models
eFigure 3. Forest Plot for Meta-analysis of C Statistics of Prediction Models of AKI in ICU
eFigure 4. Forest Plot for Meta-analysis of C Statistics of Prediction Models of AKI in General Hospitalization
eFigure 5. Drapery Plot for Meta-analysis of C Statistics of Contrast Medium Associated AKI Prediction Models
eFigure 6. Drapery Plot for Meta-analysis of C Statistics of Postoperative AKI Prediction Models
eFigure 7. Drapery Plot for Meta-analysis of C Statistics of Prediction Models of AKI in ICU
eFigure 8. Drapery Plot for Meta-analysis of C Statistics of Prediction Models of AKI in General Hospitalization
eFigure 9. Summary ROC Curves of C Statistics of Contrast Medium Associated AKI Prediction Models
eFigure 10. Summary ROC curves of C Statistics of Postoperative AKI Prediction Models
eFigure 11. Summary ROC Curves of C Statistics of Prediction Models of AKI in ICU
eFigure 12. Summary ROC Curves of C Statistics of Prediction Models of AKI in General Hospitalization
eFigure 13. Bayesian Analysis of C Statistics of AKI Prediction Models in All Clinical Settings
eFigure 14. Bayesian Analysis of C Statistics of Contrast Medium Associated AKI Prediction Models
eFigure 15. Bayesian Analysis of C Statistics of Postoperative AKI Prediction Models
eFigure 16. Bayesian Analysis of C Statistics of Prediction Models of AKI in ICU
eFigure 17. Bayesian Analysis of C Statistics of Prediction Models of AKI in General Hospitalization

© 2023 Feng Y et al. *JAMA Network Open.*

eFigure 18. Metaregression Analysis of Variables That Were Suspected to Be the Sources of Heterogeneities Against C Statistics (Weighted Against Standard Errors of C Statistics)
eFigure 19. The Baujat Plot for Between-Study Heterogeneity Analysis of Contrast Medium Associated AKI Prediction Models
eFigure 20. The Baujat Plot for Between-Study Heterogeneity Analysis of Postoperative AKI Prediction Models
eFigure 21. The Baujat Plot for Between-Study Heterogeneity Analysis of Prediction Models of AKI in ICU
eFigure 22. The Baujat Plot for Between-Study Heterogeneity Analysis of Prediction Models of AKI in General Hospitalization
eFigure 23. Influence Analysis for Between-Study Heterogeneity Analysis of Contrast Medium Associated AKI Prediction Models
eFigure 24. Influence Analysis for Between-Study Heterogeneity Analysis of Postoperative AKI Prediction Models
eFigure 25. Influence Analysis for Between-Study Heterogeneity Analysis of Prediction Models of AKI in ICU
eFigure 26. Influence Analysis for Between-Study Heterogeneity Analysis of Prediction Models of AKI in General Hospitalization
eFigure 27. The Comparisons Between Reported Standard Error and Estimated Error of C Statistics of Each Study in the Whole Population and Each Subgroup
eFigure 28. Funnel Plot Analysis for Meta-analysis of C Statistics of Contrast Medium Associated AKI Prediction Models
eFigure 29. Funnel Plot Analysis for Meta-analysis of C Statistics of Postoperative AKI Prediction Models
eFigure 30. Funnel Plot Analysis for Meta-analysis of C Statistics of Prediction Models of AKI in ICU
eFigure 31. Funnel Plot Analysis for Meta-analysis of C Statistics of Prediction Models of AKI in General Hospitalization
eFigure 32. Risk of Bias Assessment Results Using PROBAST Tool for Prediction Models on Contrast Medium Associated AKI
eFigure 33. Risk of Bias Assessment Results Using PROBAST Tool for Prediction Models on Postoperative AKI
eFigure 34. Risk of Bias Assessment Results Using PROBAST Tool for Prediction Models on Postoperative AKI
eFigure 35. Risk of Bias Assessment Results Using PROBAST Tool for Prediction Models of AKI in General Hospitalization
eReferences.

This supplemental material has been provided by the authors to give readers additional information about their work.

eTable 1. Literature Search Strategy

1. Embase (Embase Classic+Embase 1947 to April 08, 2021)
1. exp Acute Renal Failure/
2. exp Acute kidney injury/
3. acute renal failure).tw.
4. (acute kidney injury).tw.
5. (ARF).tw.
6. (AKI).tw.
7. 1 OR 2 OR 3 OR 4 OR 5 OR 6
8. (diagnos$ adj5 model).tw.
9. (prognos$ adj5 model).tw.
10. (predict$ adj5 model).tw.
11. exp machine learning/
12. (machine learning).tw.
13. 11 OR 12
14. exp artificial intelligence/
15. (artificial intelligence).tw.
16. 14 OR 15
17. exp algorithm/
18. algorithm.tw.
19. 17 OR 18
20. exp deep learning/
21. (deep learning).tw.
22. 20 OR 21
23. 8 OR 9 OR 10 OR 13 OR 16 OR 19 OR 22
24. 7 AND 23
2. Medline (via Pubmed, from 1946 through April 08, 2021)
((Acute Renal Failure) OR (Acute kidney injury) OR (ARF) OR (AKI)) AND ((diagnos* model) OR (prognos* model) OR (machine learning) OR (artificial intelligence) OR algorithm OR (deep learning))

© 2023 Feng Y et al. *JAMA Network Open.*

eTable 2. Details of Extracted Variables

Categories	Details
Study characteristics	description of the studied population, AKI definition, number of AKI events, number of models reported and total sample size of development population
Covariate information	number of candidate predictive variables, possibility of overlap between the timing of predictive variables and the outcome window, availability of predictive variables at the time of prediction and baseline serum creatinine
Modelling method	algorithm used to develop prediction models
Performance assessments	discrimination and calibration measures reported in the publication, including any summaries of discrimination and calibration as recommended in the TRIPOD statement [1]
Validation methods	use of internal and external validation methods

© 2023 Feng Y et al. *JAMA Network Open.*

eTable 3. Details of Data Synthesis

Pooled pool c-statistics were used to evaluate the discrimination performance, which were derived from a random-effects model due to the potentially high between-study heterogeneity. Missing standard errors were estimated using the method proposed by Hanley and McNeil [2], which approximated standard errors from the reported c-statistic, the sample size and the total number of events. A summary ROC (sROC) curve with a 95% confidence interval (CI) was also generated using the hierarchical summary receiver operating characteristics (HSROC) model [3] to evaluate the pooled discrimination ability from the studies where the requisite data was available. To generate the sROC curve, C statistics and corresponding sensitivity and specificity data that were reported only graphically were extracted manually using GetData Graph Digitizer (version 2.26). Fagan diagrams were used to examine model effects upon the post-test probability [4]. The included studies were weighted against the inverse of the variance of effect estimate. Hartung-Knapp (HK) adjustments was used to calculate the confidence interval of pooled effect [5]. Forest plot and Drapery plot [6] were used to report effect size and corresponding 95% confidence intervals (CIs) for each study, and the pooled estimate. The pooled 95% prediction interval (PI) was calculated using the equation proposed by Higgins et al. [7] which calculates prediction interval based on a t distribution with K-2 degrees of freedom, where K corresponds to the number of studies included.

Between-study heterogeneity variance (tau^2) was calculated using restricted maximum likelihood (REML) estimator [8]. I^2 statistic [9], which is based on the Q test and denotes an estimate of the percentage of error due to between-study variations, was used to quantify between-study heterogeneity. I^2 values of ≤25%, >25 to 75% and >75% were considered as low, moderate and high heterogeneity, respectively. Between-study heterogeneity was further explored using several methods, including subgroup analysis (studies grouped according to clinical setting, study design, AKI definitions, geographical region, AKI frequency, model development methods, number of predictive variables, risk of bias, overlap of predictor and outcome at prediction, and predictor availability at predicton), Baujat plot[10], influence analysis, and leave-one-out meta-analysis[11]. Publication bias was evaluated using contour-enhanced funnel plot analysis [12], in which funnel plot asymmetry was also adjusted using Duval &

© 2023 Feng Y et al. *JAMA Network Open.*

Tweedie trim and fill method [13] to further illustrate the asymmetry in the funnel plot. In addition, Egger's regression test [14] was also used to quantify the asymmetry in the funnel plot.

Data analysis was performed using Microsoft Excel (Microsoft Corporation, Redmond, WA), Stata 14 MP (STATA, College Station, TX, USA), and R 4.0.3 (The R Core Team, R Foundation for Statistical Computing, Vienna, Austria) running on R Studio 1.4.1103 (R Studio Team, R Studio Inc. Boston, MA, USA), with "forestplot", "metamisc", "metafor", "dmetar", "PerformanceAnalytics", and "robvis" packages.

© 2023 Feng Y et al. *JAMA Network Open.*

eTable 4. Details of Basic Characteristics of the Included Prediction Models

Author/Year	Study Design	Study Period	Study Population	Study Region	AKI Definition			sample size	
					Class	Definition	Baseline Cr	Total	AKI
**I. Prediction models of AKI induced by contrast medium (n=26)**									
Ando, G. et al 2014[15]	Retrospective	2009–2011	patients referred for primary PCI	North America	Self-defined	CI-AKI, defined as an absolute increase in serum creatinine concentration ≥ 0.5 mg/dL or an increase ≥25% from baseline within 72 hours after the administration of contrast medium, without any other plausible cause	on hospital admission	470	25
Brown, J. R. et al 2015[16]	Retrospective	January 1, 2009 to October 1, 2013	patients undergoing cardiac catheterization or PCI	North America	Self-defined	CIN: serum Cr increased ≥0.5 mg/dL within 7 days after surgery	most recent serum Cr between 365 and 7 days prior to the procedure	115633	3763
Chen, Y. L. et al 2014[17]	Retrospective	January 2009 to May 2011	patients who underwent PCI	Asian Pacific	Self-defined	CI-AKI, defined as an increase of ≥ 25% or ≥0.5 mg/dL serum creatinine within five days after PCI	serum creatinine before surgery	1500	246
Duan, C. et al 2017[18]	Prospective	January 2010 to October 2012	patients who underwent coronary angiography or PCI	Asian Pacific	Self-defined	an increase in the level of serum creatinine by 50% or 0.3 mg/dL from baseline	sCr measured before procedure	1074	64
Fan, P. C. et al 2018[19]	Retrospective	January 1, 1997 to December 31, 2011	patients with a diagnosis of ACS who underwent PCI	Asian Pacific	ICD-codes	ICD-9-CM code	not reported	57530	2670

© 2023 Feng Y et al. *JAMA Network Open.*

Ghani, A. A et al 2009[20]	Prospective	March to May 2005	patients admitted for PCI	Middle East	Self-defined	an increase in sCr of ⩾44.2umol/L	sCr before the procedure	247	13
Gurm, H. S. et al 2013[21]	Retrospective	January 1, 2010 to June 30, 2012	patients undergoing PCI	North America	Self-defined	CIN, defined as > 0.5 mg/dL absolute increase in serum Cr from baseline	sCr within a month before procedure, or the closest one to the procedure if there were multiple assessment	48001	1243
Hu, X. et al 2017[22]	Prospective	May 2014 to July 2015	adults who underwent selected PCI	Asian Pacific	Self-defined	CIN, an increase in serum Cr of 0.5 mg/dL above baseline within 48-72 hours after PCI	sCr prior to pre-procedural hydration	192	32
Huang, C. et al 2018[23]	Retrospective	June 1, 2009 to June 30, 2011	individuals who underwent PCI	North America	KDIGO	a change in post procedure Cr larger than 0.3 mg/dl or a 1.5-fold increase from before the procedure	sCr before the procedure	947091	69826
Ibrahim, N. E. et al 2019[24]	Prospective	2008 to 2011	patients undergoing coronary and/or peripheral angiography with or without intervention	North America	KDIGO	an abrupt reduction in kidney function with an absolute increase in serum Cr of more than or equal to 0.3 mg/dL, a percentage increase in sCr of ⩾50%, or a reduction in urine output within 7 days after contrast exposure	sCr immediately before the angiographic procedure	889	43
Inohara, T.. et al 2015[25]	Retrospective	January 2011 to March 2013	patient undergoing PCI procedures	Asian Pacific	KDIGO	increase in sCr of 50% or 0.3 mg/dL after PCI	preprocedural sCr	3957	358
Jeon, J. et al 2019[26]	Retrospective	October 2009 and July 2017	cancer patients underging CT under preventive measures	Asian Pacific	Self-defined	sCr increase more than 25% within 2-6 days after	sCr concentration on the day of CECT	2240	55

© 2023 Feng Y et al. *JAMA Network Open.*

						contrast-enhanced CT(CECT)			
Ji, L. et al 2015[27]	Retrospective	December 2011 to June 2013	patient who underwent PCI procedures, including emergency PCI because of ACS	Asian Pacific	Self-defined	an increase of >25% or >0.5mg/dl (44.2umol/L) in baseline serum creatinine level within 72 hours after use of contrast agents, without other factors lead to renal impairment according to ESUR guideline	sCr before the procedure	565	68
Lian, D. et al 2017[28]	Retrospective	August 2009 to February 2013	patients aged >65 years and who underwent elective coronary angiography (CAG)	Asian Pacific	KDIGO	a maximal increase in sCr by ⩾0.3 mg/dl or ⩾50% from the baseline within 48-72 hours after CM exposure	Scr measured before CM exposure	459	48
Lin, K. Y. et al 2017[29]	Prospective	January 2010 and December 2013	patients who underwent coronary angiography or PCI	Asian Pacific	Self-defined	an absolute increase in the sCr concentration by 0.5mg/dl compared to the baseline value within 72 hours of contrast exposure	not reported	461	32
Liu, L. et al 2020[30]	Prospective	January 2010 to October 2012	adult patients with baseline hypoalbuminemia who underwent CAG or PCI	Asian Pacific	KDIGO	an increase of ⩾ 0.3 mg/dl or 50% in sCr compared to baseline in the 48 to 72 hours after CAG/PCI.	not reported	848	71
Liu, Y. et al 2020[31]	Retrospective	January 2010 to December 2013	adult patients undergoing PCI or coronary angiogram (CAG)	Asian Pacific	Self-defined	CIN, defined as an increase in sCr⩾ 0.5 mg/dl from baseline within 48-72 hours after CAG or PCI.	sCr concentration measured preoperatively	2428	78

© 2023 Feng Y et al. *JAMA Network Open.*

Sun, L. et al 2020[32]	Retrospective	January 2012 to January 2018	adult patients with clinically diagnosed acute myocardial infarction (AMI)	Asian Pacific	KDIGO	KDIGO, CI-AKI, defined as an absolute increase in sCr ⩾0.3 mg/dl within 48 hours after procedure or an increase of ⩾ 150% from baseline, or urine volume <0.5ml/kg/h for 6 hours	sCr within the prior 7 days	1122	169
Tsai, T. T. et al 2014[33]	Retrospective	June 1 2009 to June 30 2011	patients receiving PCI	North America	AKIN	AKIN	pre-operative sCr	662504	48593
Yin, W. J. et al 2017[34]	Retrospective	September 2007 to January 2015	adult patients who were treated with contrast medium for coronary angiography or PCI or received intravenous CM such as for CT or endovascular procedures	Asian Pacific	Self-defined	an increase of sCr of 0.5 mg/dL (44.2 umol/L) or 25% relative increase in sCr from the baseline to 72 hours after exposure to CM	the earliest sCr within 14 days before a procedure	7040	942
Zambetti, B. R. et al 2017[35]	Retrospective	January 2008 to September 2013	patients referred for primary PCI treatment of STEMI	North America	RIFLE	RIFLE	not reported	408	68
Zhou, X. et al 2018[36]	Retrospective	January 2013 to July 2017	patients with AMI undergoing CAG or PCI	Asian Pacific	Self-defined	CI-AKI, an absolute increase of serum Cr ⩾0.3 mg/dL or increase to ⩾ 150% from baseline	sCr measured immediately at the time of admission	534	114
Yao Zhifeng et al 2020[37]	Retrospective	not reported	patients with diabetes who underwent primary PCI for chronic stable coronary artery disease or	Asian Pacific	Self-defined	CIN, an increase in serum Cr of 0.5 mg/dL or 25% within 72 hours after contrast exposure	sCr at the time of admission before the procedure	742	94

© 2023 Feng Y et al. *JAMA Network Open.*

			urgent PCI for ACS						
Roxana Mehran et al 2004[38]	Retrospective	a period of 6 years	patients undergoing PCI	North America	Self-defined	CIN, defined as an increase of sCr⩾ 0.5 mg/dl or ⩾ 25% in pre-PCI sCr	sCr before procedure	5571	729
Guiseppe Ando et al 2012[39]	Retrospective	January 2008 to June 2011	patients undergoing primary PCI for STEMI	Europe	Self-defined	CIN, defined as an absolute increase in sCr ⩾0.5 mg/dL or an increase⩾ 25% from baseline	sCr at admission	481	24
Dimitrios Tziakas et al 2011[40]	Retrospective	September 2008 to January 2010	patients who were treated with PCI on an elective or emergency basis	Europe	Self-defined	CIN, defined as an increase of sCr⩾ 0.5 mg/dl or ⩾ 25% in pre-PCI sCr	sCr measured 18 to 24 hours before procedure	488	50
**II. Prediction models of AKI induced by agents other than contrast medium (n=3)**									
Jeon, N. et al 2019[41]	Retrospective	January 2012 to December 2013 (UF Health Shands) or March 2013 to December 2013 (UF Health Jacksonville)	patients who received a nephrotoxic medication for systemic use during admission	Europe	KDIGO	KDIGO stage 2 criteria	most recent sCr within 2 days prior to a given risk model day	62561	550
Motwani, S. S. et al 2018[42]	Retrospective	2005 to 2014	adult patients who had been treated with cisplatin	North America	Self-defined	a ⩾0.3 mg/dl rise in sCr from baseline to peak measurement after the first course of cisplatin	sCr within the month before the index date	2118	288
Xu, N. et al 2020[43]	Retrospective	January 1, 2016 to December 31, 2018	adult patients who received vancomycin treatment for ⩾ 48 hours during hospitalization	Asian Pacific	KDIGO	KDIGO	sCr measured on the day before vancomycin administration	341	55
**III. Prediction models of post-operative AKI (n=64)**									
STARSurg Collaborative, 2018[44]	Prospective	September 2015 to November 2015	Adult patients (aged 18 years or above) undergoing elective or	Europe	KDIGO	meeting Kidney Disease Improving Global Outcomes (KDIGO) criteria11	a preadmission value measured within 90 days preceding the	4544	646

© 2023 Feng Y et al. *JAMA Network Open.*

			emergency gastrointestinal resection, liver resection, or reversal of ileostomy or colostomy, using any operative approach			(26·5 μmol/l increase in serum creatinine concentration within 48 h, or a greater than 50 per cent increase in creatinine concentration from baseline within 7 days) or underwent unplanned renal replacement therapy	date of surgery; the first preoperative value measured on the index admission; the last value measured on index admission before surgery, whichever the closest to the index surgery		
Adhikari et al, 2019[45]	Retrospective	2000 to 2010	adult that underwent surgery	North America	KDIGO	defined using the KDIGO criteria as at least a 50% or 0.3 mg/dl increase in serum creatinine relative to the reference creatinine	1) minimum serum Cr within 7 days of the index hospitalization; 2) median Cr obtained within 8-365 days prior to admission; 3) estimated reference serum Cr by MDRD	2038	1163
Al-Jefri et al, 2020[46]	Retrospective	2008-2015	Patients (age≥18) who underwent five different types of surgery (abdominal, cardiac, retroperitoneal, thoracic and vascular)	North America	KDIGO	based on the sCr changes according to the KDIGO definition	first Cr at admission or baseline level in the Alberta Kidney Disease Network (AKDN) database	40890	2745
Antunes et al, 2009[47]	Retrospective	January 1992 to December 2001	patients undergoing CABG	Europe	Self-defined	At least one of the following: (1) a postoperative serum creatinine (Scr) level 2.1 mg/dl plus an increase in	at admission	4567	256

© 2023 Feng Y et al. *JAMA Network Open.*

						the Scr level 0.9 mg/dl from preoperative to maximum postoperative values if preoperative Scr 2.0 mg/dl; (2) an increase in the Scr level 1.5 mg/dl if baseline Scr 2.0 mg/dl; (3) a new requirement for dialysis			
Bell et al, 2015[48]	Retrospective	1 Jan 2005 to 31 Dec 2011	all adults aged more than 18 years who underwent orthopaedic surgical procedures	Europe	KDIGO	development of any severity of acute kidney injury (stages 1-3 based on KDIGO criteria)	the serum creatinine most recent before surgery	6220	672
Berg et al, 2013[49]	Prospective	2000 to 2007	adult patients undergoing open heart surgery	Europe	KDIGO	defined as either a relative increase in serum creatinine concentration of at least 50% after surgery compared with the concentration before surgery, an absolute increase in serum creatinine concentration by 26.4 mmol/l (0.3 mg dl/L) or more, or a new requirement for dialysis	serum creatinine 1 to 2 days before surgery or at admission in emergency patients	4978	633
Bihorac et al, 2019[50]	Retrospective	1 Jan 2000 to 30 Nov 2010	patients with age greater or equal to 18 years, admitted for longer than	North America	Self-defined	AKI by consensus criteria occurring anytime during	preoperative sCr	51457	20025

© 2023 Feng Y et al. *JAMA Network Open.*

			24hours after anytype of inpatient operative procedure			hospitalization after the index surgery			
Birnie et al, 2014[51]	Prospective	1996 - 2010 at University Hospital of Bristol, 2002-2010 at University Hospitals of Birmingham National Health Services (NHS) Foundation Trust, 2004-2010 at Wolverhampton Heart and Lung Center	all patients aged ⩾16 years, who underwent cardiac surgery, with or without cardiopulmonary bypass (CBP), and including those who underwent surgery to the thoracic aorta	Europe	KDIGO	KDIGO guidelines	preoperative sCr	16527	3794
Che et al, 2019[52]	Retrospective	January 1, 2006 to December 31, 2009	patients undergoing cardiac surgery	Asian Pacific	KDIGO	severe AKI, defined as stage 2 or 3 according to the KDIGO definition, doubling of sCr from the baseline value, or renal replacement therapy requiring dialysis.	latest serum Cr measurement recorded after admission but before surgery	1692	175
Chen et al, 2020[53]	Retrospective	not reported	patients who underwent cardiac surgery	Asian Pacific	AKIN	severe AKI, defined AKIN stages 2 or 3	serum creatinine before surgery	204	42
Coulson et al, 2021[54]	Retrospective	September 1, 2016 to December 31, 2018	patients admitted for cardiac surgery	Asian Pacific	KDIGO	AKI was defined using KDIGO criteria and creatinine only	immediate preoperative creatinine value		
Mehmet et al, 2020[55]	Retrospective	May 2008 to February 2018	patients who underwent heart valve replacement surgery	Middle East	KDIGO	KDIGO criteria	sCr within 3 days before surgery	219	85

© 2023 Feng Y et al. *JAMA Network Open.*

Du et al, 2021[56]	Retrospective	June 2017 to July 2020	patients who underwent PCI or CPB surgery	Asian Pacific	AKIN	AKIN criteria	sCr 1-2 days before surgery	350	58
Grimm et al, 2015[57]	Retrospective	2005 to 2012	adult patients who received single or double lung transplantation	North America	RRT	renal failure necessitating haemodialysis	not reported	8771	
Guan et al, 2019[58]	Retrospective	October 2012 to October 2017	patients who received cardiac surgery	Asian Pacific	KDIGO	KDIGO criteria	the latest Scr value before surgery	4395	932
Hofer et al, 2020[59]	Retrospective	March 17, 2013 to July 16, 2016	adult surgical cases	North America	AKIN	AKIN criteria	preoperative sCr, the most recent Cr within 6 months prior to surgery	47985	3871
Hu et al, 2021[60]	Retrospective	January 2005 to December 2010	patients aged ⩾ 60 years who underwent valve surgery and/or CABG with cardiopulmonary bypass	Asian Pacific	KDIGO	KDIGO	the last preoperative serum creatinine	597	369
Hu et al, 2020[61]	Retrospective	March 2013 to December 2017	patients with renal cell carcinoma who had undergone radical or partial nephrectomy	Asian Pacific	KDIGO	KDIGO	latest serum Cr within 30 days preoperatively	265	73
Jiang et al, 2016[62]	Prospective	January 2010 to April 2013	cardiac surgery patients	Asian Pacific	KDIGO	increase in sCr by ⩾0.3 mg/dL (⩾ 26.5 μmol/L) within 48 hours; or increase in sCr to ⩾1.5 times baseline that is known or presumed to have occurred within the prior 7 days or urine volume <0.5 mL/kg per hour for 6 hours	not reported	6081	2159

© 2023 Feng Y et al. *JAMA Network Open.*

Jorge-Monjas et al, 2016[63]	Prospective	January 2012 to January 2014	adult patients with normal renal function, scheduled for CABG and/or valve surgery with CPB	Europe	KDIGO+ICD-codes	RIFLE criteria	sCr at hospital admission	810	137
Kalisvaart et al, 2019[64]	Retrospective	Queen Elizabeth Hospital in Birmingham, UK (2007–2015) and the Erasmus MC University Medical Centre in Rotterdam, the Netherlands (2008–2014)	adult patients who underwent primary, orthotopic deceased-donor liver transplantation	Europe	KDIGO	KDIGO stage 2 & 3	not reported	1230	429
Kashani et al, 2015[65]	Retrospective	January 3, 2003 to May 29, 2008	adult patients who underwent a major open vascular surgery	North America	AKIN	AKIN criteria, using sCr levels and hourly urine output	not reported	845	258
Kim et al, 2014[66]	Retrospective	January 2008 to December 2011	patients who underwent liver transplantation	Asian Pacific	RRT	AKI needing CRRT	preoperative sCr	157	42
Kim et al, 2011[67]	Retrospective	April 2006 to July 2007	adult patients who underwent isolated OPCAB (off-pump CABG)	Asian Pacific	AKIN	AKIN criteria	preoperative sCr	448	34
Ko et al, 2020[68]	Retrospective	June 2018 to May 2019	patients who underwent unilateral or bi-lateral TKA	Asian Pacific	KDIGO	KDIGO	latest preoperative level within 6 months before surgery	5302	539
Lee et al, 2018[69]	Retrospective	2008 to 2015	patients who underwent coronary artery surgery, valve replacement, or thoracic aortic surgery	Asian Pacific	KDIGO	KDIGO	the most recent sCr measured before surgery	1005	282

© 2023 Feng Y et al. *JAMA Network Open.*

Lee et al, 2018[70]	Retrospective	November 2004 to December 2015	patients who underwent living donor liver transplantation or deceased donor liver transplantation	Asian Pacific	AKIN	AKIN criteria	the most recent sCr measured before surgery	848	254
Legrand et al, 2013[71]	Retrospective	January 2000 to December 2010	patients diagnosed of infectious endocarditis who underwent cardiac surgery with cardiopulmonary bypass	Europe	AKIN	AKIN	Scr before hospital admission or the lowest sCr measured during the hospital stay	202	120
Lei et al, 2020[72]	Retrospective	January 2008 to October 2015	patients who underwent primary hepatocellular carcinoma resection	Asian Pacific	KDIGO	KDIGO	not reported	1173	77
Lei et al, 2019[73]	Retrospective	January 1, 2014 to April 30, 2018	adult patients undergoing noncardiac surgery	North America	KDIGO	KDIGO	lowest sCr measurement value within 7 days before the start of surgery	34121	4318
Li et al, 2020[74]	Retrospective	January 1, 2013 to December 31, 2014	patients receiving cardiac surgeries	Asian Pacific	KDIGO	KDIGO	not reported	3639	1364
Lin et al, 2020[75]	Retrospective	January 2012 to December 2017	patients who had an impaired LVEF value (<50%) and underwent an isolated CABG procedure	Asian Pacific	KDIGO	a maximal increase in sCr by ⩾0.3 mg/dl within 48 hours, or by ⩾1.5 times baseline in 7 days	not reported	1208	90
Liu et al, 2016[76]	Retrospective	October 2007 to June 2015	hypertensive adult patients who underwent only one elective surgery	Asian Pacific	KDIGO	KDIGO using the peak-to-nadir sCr	lowest sCr recorded within the first 7 days of hospitalization	17089	1396
Martini et al, 2019[77]	Retrospective	2008 to 2017	patients who underwent robot-assisted partial	North America	RIFLE	RIFLE criteria	preoperative sCr	1190	274

© 2023 Feng Y et al. *JAMA Network Open.*

			nephrectomy for suspected renal cell carcinoma						
McBride et al, 2019[78]	Retrospective	May 2012 to August 2013	cardiac patients who scheduled for elective cardiac surgery	Europe	Self-defined	eGFR drop ⩾25% from preoperative baseline at any time postoperatively	preoperative sCr	344	71
Metzger et al, 2016[79]	Retrospective	not reported	patients undergoing cardiac surgery	Europe	AKIN	AKIN stage 1	not reported	120	60
Meyer et al, 2018[80]	Retrospective	Jan 1, 2000 to Dec 31, 2016	patients who underwent major open heart surgery	Europe	Self-defined	renal failure	not reported	813	407
Nah et al, 2016[81]	Prospective	July 2008 to October 2011	patients who underwent CABG, valve surgery or combined CABG/valve surgery	Asian Pacific	AKIN	AKIN criteria	preoperative sCr that obtained closest to surgery	2385	728
Ortega-Loubon et al, 2018[82]	Retrospective	January 2012 to December 2016	patients undergoing elective or urgent isolated primary CABG surgery under CPB	Europe	RIFLE	RIFLE criteria	the most recent sCr measured before surgery	435	45
Pannu et al, 2016[83]	Prospective	Jan 1, 2004 to Mar 31, 2009	patients who underwent cardiac catheterization	North America	KDIGO+RRT	AKI based on KDIGO guideline and treated with RRT	within 6 months before surgery	6061	154
Paparella et al, 2014[84]	Retrospective	Jan 1 2011 to Dec 31 2011	adult patients undergoing cardiac operations with extracorporeal circulation	Europe	RRT	AKI needing RRT	not reported	1642	71
Penny-Dimri et al, 2020[85]	Retrospective	April 2001 to December 2016	patients who underwent cardiac surgery	Asian Pacific	Self-defined	(1) A new postoperative and in-hospital serum creatinine greater than 200 mmol/L (2.26 mg/dL),	preoperative sCr	97964	4599

© 2023 Feng Y et al. *JAMA Network Open.*

						AND a doubling or greater increase in creatinine over the baseline preoperative value, AND the patient did not require preoperative renal replacement therapy (RRT), (2) a new in-hospital requirement for RRT			
Ramonell et al, 2016[86]	Retrospective	2009	patients who underwent colorectal surgeries	North America	Self-defined	ARF (a patient who did not require dialysis preoperatively with worsening of renal dysfunction postoperatively requiring haemodialysis, peritoneal dialysis, hemofiltration, hemodiafiltration, or ultrafiltration) or progressive renal insufficiency (the reduced capacity of the kidney to perform its function as evidenced by a rise in creatinine of >2 mg/dL from preoperative value, but with no requirement for dialysis within 30 days of the operation)	not reported	21720	357

© 2023 Feng Y et al. *JAMA Network Open.*

Rank et al, 2020[87]	Retrospective	October 2012 to February 2018	adult patients who were admitted for cardiothoracic surgery	Europe	KDIGO	KDIGO stage 2 or 3	last sCr before surgery or the first postoperative value	2224	
Rueggeberg et al, 2008[88]	Retrospective	August 2000 to June 2001	orthotopic liver transplantation recipients	Europe	Self-defined	serum Cr ⩾132 umol/l, and requirement of RRT	not reported	71	13
Simonini et al, 2014[89]	Retrospective	December 2009 to July 2012	patients undergoing cardiac surgery	Europe	AKIN	AKIN stage 2 or 3	sCr measured within 2 months prior to surgery	802	79
Slankamenac et al, 2013[90]	Retrospective	1 July 2002 to 31 October 2007	patients undergoing liver surgery	Europe	RIFLE	RIFLE criteria (R)	preoperative sCr	549	82
Slankamenac et al, 2009[91]	Retrospective	1 July 2002 to 31 October 2007	patients undergoing liver resection	Europe	RIFLE	RIFLE criteria (R)	preoperative sCr	380	58
Thakar et al, 2005[92]	Retrospective	April 1993 to December 2002	patients undergoing open-hear surgery	North America	RRT	ARF needing dialysis	not reported	15838	269
Tian et al, 2021[93]	Retrospective	January 1, 2012 to January 1, 2019	adult patients undergoing open heart surgery	Asian Pacific	KDIGO	KDIGO	not reported	43799	15140
Trongtrakul et al, 2020[94]	Retrospective	April 2010 to January 2013	major non-cardiothoracic surgery patients who were admitted to ICU	Asian Pacific	KDIGO	KDIGO	the lowest sCr during ICU admission or the best sCr 3 months preceding ICU admission	3474	333
Tseng et al, 2020[95]	Retrospective	August 2016 to August 2018	patients who underwent CABG, valve surgery or combined CABG/valve surgery	Asian Pacific	KDIGO	KDIGO	sCr before surgery	469	114
Xue et al, 2021[96]	Retrospective	June 1, 2012 to August 31, 2016	adult patients undergoing surgery	North America	KDIGO	KDIGO	not reported	111888	652

© 2023 Feng Y et al. *JAMA Network Open.*

Yayac et al, 2021[97]	Retrospective	2005 - 2017	patients who underwent primary, elective, unilateral THA and TKA	North America	Self-defined	an increase in Cr by 50% or 0.3 mg/dL from preoperative to postoperative serum level	the most recent sCr measured within 60 days before surgery	20800	814
Zhang et al, 2020[98]	Retrospective	October 2012 to October 2017	patients undergoing intracranial aneurysm clipping surgery	Asian Pacific	KDIGO	KDIGO	the first sCr measured during hospitalization	365	68
Zhou et al, 2020[99]	Retrospective	2009 to 2017	patients who underwent thoracoabdominal aorta aneurysm replacement	Asian Pacific	RRT	acute renal failure that required CRRT	not reported	212	27
Jeremiah et al, 2013[100]	Prospective	2001 to 2005	patients undergoing isolated CABG surgery	North America	Self-defined	severe renal insufficiency defined as <30 eGFR after CABG surgery	latest preoperative level	8363	229
Rahendra et al, 2006[101]	Retrospective	July 2002 to December 2004	patients undergoing CABG, mitral or aortic surgery, or both	North America	RRT	AKI requiring dialysis	not reported	4449524	6451
Duminda et al, 2007[102]	Retrospective	May 1999 to July 2014	patients who underwent cardiac surgery under CPB	North America	RRT	AKI requiring RRT	not reported	10751	139
Paloma et al, 2007[103]	Retrospective	July 2003 to July 2005	adults undergoing elective CABG surgery, valve replacement or both	South America	Self-defined	an increase of Cr > 2.0 mg/dl or >50% over baseline value	last sCr before surgery or the first measurement during hospitalization	603	66
Sevag et al, 2012[104]	Retrospective	April 2000 to January 2008	patients who underwent cardiac surgery	North America	RRT	AKI requiring dialysis	sCr before procedure	25898	429
Sachin et al, 2009[105]	Retrospective	2005 to 2006	patients undergoing general surgery	North America	RRT	ACS-NSQIP defined progressive renal insufficiency	the most recent sCr measured	56519	561

© 2023 Feng Y et al. *JAMA Network Open.*

						or AKI necessitating dialysis	within 90 days before surgery		
Ng et al, 2014[106]	Retrospective	2001 to 2009	patients who underwent cardiac surgery	Asian Pacific	Self-defined	increased sCr to > 200umol/L, or doubling or greater increase in sCr over preoperative value, or new requirement for RRT	preoperative sCr	28422	1642
Kim et al, 2013[107]	Retrospective	1997 to 2010	patients undergoing aortic surgery with cardiopulmonary bypass	Asian Pacific	RIFLE	RIFLE	sCr measured before surgery	417	148
**IV. Prediction models of AKI in ICU (n=21)**									
An, S. et al 2020[108]	Retrospective	January 2017 to December 2017	patients admitted to neurosurgery ICU	Asian Pacific	KDIGO	standard of stage 1 (serum creatinine, 1.5–1.9 times baseline or ⩾26.5 μmol/L increase; urine output, <0.5 mL/kg/h for 6–12 hours) AKI based on KDIGO criteria	Cr at admission	583	71
Asada, T. et al 2016[109]	Prospective	April 2010 to March 2011	Patients aged 18 years or older who were admitted to the ICU	Asian Pacific	KDIGO	AKI was determined by changes in serum creatinine levels according to KDIGO criteria and was defined as an increase in serum creatinine level by 0.3 mg/dL within 48 h or at least 1.5 times over baseline levels	minimum value among all outpatient values within the last 6 months prior to hospital admission, inpatient value before ICU admission and last value before hospital discharge	249	147

© 2023 Feng Y et al. *JAMA Network Open.*

Bhatraju, P. K. et al 2019[110]	Prospective	1999-2010	Adult patients who were enrolled in the ICU and met two or more systemic inflammatory response syndrome criteria	North America	KDIGO	Severe AKI within 72 hours after enrolment (KDIGO stage 2 or 3) was defined by ⩾ 100% increase in serum creatinine from reference or a serum creatinine $4 mg/dl after study enrolment	maximum day 0 serum creatinine	749	62
Chen, Y. et al 2019[111]	Retrospective	September 2016 and December 2017	patients admitted in surgical ICU	Asian Pacific	KDIGO	KDIGO	average serum Cr level during the first 3 ICU days	499	149
Chiofolo, C. et al 2019[112]	Retrospective	October 1 2004 and April 30, 2011	adult (⩾18) ICU patients, including medical, surgical, and mixed ICU	North America	AKIN	AKIN stage 2-3	not reported	4572	616
Deng, F. et al 2020[113]	Retrospective	June 1, 2001 to October 31, 2012	ICU patients with sepsis	North America	KDIGO	KDIGO criteria	not reported	2042	1167
Deng, Y. et al 2017[114]	Prospective	October 2014 to February 2016	patient ⩾18 years that admitted in general ICU	Asian Pacific	KDIGO	KDIGO criteria	EARLYARF trial's criteria using 5 rules ranked in descending order of preference	1084	326
Deng, Y. et al 2020[115]	Prospective	October 2014 to July 2016	patients who underwent resection of intracranial space-occupying lesions and were admitted to neurosurgical ICU	Asian Pacific	KDIGO	KDIGO criteria	EARLYARF tial's criteria using 5 rules ranked in descending order of preference	605	67
Ferrari, F. et al 2019[116]	Retrospective	1 June 2016 to 31 March 2017	critically ill patients admitted to ICU	Europe	KDIGO	KDIGO criteria	pre-morbid sCr measured 90-180 days before ICU admission	455	176

© 2023 Feng Y et al. *JAMA Network Open.*

Ferrari, F. et al 2019[117]	Prospective	June 1 to December 31, 2016	adult patients admitted to ICU	Europe	KDIGO	KDIGO criteria	pre-morbid sCr measured 90-180 days before ICU admission, or the lowest in-patient sCr during the first 10 days of ICU admission	442	169
Flechet, M. et al 2017[118]	Retrospective	August 2007 to November 2010	adult patients in ICU	North America	KDIGO	KDIGO criteria	the lowest sCr during the 3 months prior to ICU admission for elective admissions and the lowest sCr from 3 months to 1 week before ICU admission for emergency admissions	2123	588
Malhotra, R. et al 2017[119]	Prospective	1 June, 2006 to 31 December, 2008	ICU patients	North America	KDIGO	an increase of serum Cr ⩾ 0.3 mg/dl within 48 hours or ⩾50% above the reference value within 7 days after study enrolment	mean of all Sce measurements 7-365 days prior to admission	573	127
Matsuura, R. et al 2020[120]	Retrospective	2012 to 2014	adult ICU patients	Asian Pacific	KDIGO	KDIGO stage 2/3 persisting over 72 hours	sCr on ICU day 0	4151	537
Mohamadlou, H. et al 2018[121]	Retrospective	2001 to 2012	inpatients (Stanford) and ICU patients (BIDMC)	North America	Self-defined	National Health Service (NHS) England AKI Algorithm	the lowest sCr from the past 0 to 7 days or the median value from the past 8 to 365 days	24595	1410
Nusshag, C. et al 2019[122]	Prospective	May 2017 to July 2018	ICU patients who full filled Sepsis-3 criteria	Europe	RRT	AKI needing RRT	Most recent value within seven days prior	100	19

© 2023 Feng Y et al. *JAMA Network Open.*

							to hospital admission or elective surgery; or nadir value within seven days prior to hospital admission or elective surgery; or sCr value closest to enrolment.		
Parreco, J. et al 2019[123]	Retrospective	2014 to 2015	ICU admission	North America	KDIGO	KDIGO	not reported	151098	8461
Wang, Q. et al 2019[124]	Prospective	1 March 2017 to 31 December 2017	ICU patients	Asian Pacific	KDIGO	KDIGO	sCr measured at ICU admission	656	213
Wang, Y. et al 2020[125]	Retrospective	not reported	ICU patients	Asian Pacific	KDIGO	KDIGO	not reported	10921	
Wiersema, R. et al 2020[126]	Retrospective	27 March 2015 to 22 July 2015	adult patients admitted to ICU	Europe	KDIGO	KDIGO	sCr at ICU admission	1003	414
Xie, Y. et al 2021[127]	Prospective	January 2017 to December 2019	ICU adult patients with sepsis	Asian Pacific	KDIGO	KDIGO	lowest sCr in the last 6 months before the onset of AKI or the lowest value during hospitalization without dialysis	251	49
Zimmerman, L. P. et al 2019[128]	Retrospective	2001 to 2012	ICU patients	North America	KDIGO	KDIGO	sCr on day 1 of ICU admission	23950	3954
**V. Prediction models of AKI in general hospitalizations (n=35)**									
RISK Investigators et al 2019[129]	Prospective	a single 24-hour period in September 2016	patients aged 18 years or older admitted to their AMU over a single 24-hour period in September 2016	Europe	KDIGO	hAKI, defined as a change in serum creatinine meeting the KDIGO (Kidney Disease Improving Global Outcomes) criteria	the serum creatinine concentration on admission, after exclusion of cAKI	1235	97

© 2023 Feng Y et al. *JAMA Network Open.*

						and occurring >24 hours after hospital admission			
Abusaada, K. et al 2017[130]	Retrospective	January 2011 to December 2013	all patients who present within 24 h of onset of an ischemic event with a primary diagnosis of myocardial infarction	North America	AKIN	an absolute increase in serum creatinine C0.3 mg/dl (⩾26.4 µmol/l) or a percentage increase in serum creatinine ⩾50 % (AKIN criteria)	serum creatinine at admission	1107	147
Argyropoulos, A. et al 2019[131]	Retrospective	18th March to 31st December 2015	patients admitted into the Royal Cornwall Hospital Trust (RCHT)	Europe	KDIGO	KDIGO AKI classification	serum creatinine between 24h before admission and 24h after admission, whichever is earlier	5504	216
Bihorac, A. et al 2013[132]	Retrospective	November 2003 to March 2008	adult severe blunt trauma patients	North America	RIFLE	AKI was defined by the RIFLE classification using the change in sCr during the first 28 days of hospitalization compared with reference sCr	the lowest measured sCr in the first 24 hours after trauma or the estimated sCr, whichever is lower	147	33
Breidthardt, T. et al 2012[133]	Retrospective	November 2003 to March 2005	patients presenting to the ER with lower respiratory tract infections	North America	AKIN	by AKIN classification	not reported	372	16
Cheng, P. et al 2017[134]	Retrospective	November 2007 to March 2016	adult admission	North America	KDIGO	KDIGO criteria as an increase in serum creatinine >= 0.3 mg/dl within 48 hours, >=50% in 7 days	either the last measurement within 2-day time window prior to admission or the first available	48955	4405

© 2023 Feng Y et al. *JAMA Network Open.*

							measurement during the stay		
Cronin, R. M. et al 2015[135]	Retrospective	January 1, 2003 to December 31, 2012	inpatients	North America	KDIGO	AKI stage 2 or 3	the mean outpatient creatinine value from –365 days up to –7 days	6390410	15684
Drawz, P. E. et al 2008[136]	Retrospective	starting with January 1, 2013	inpatients	North America	Self-defined	an increase in serum creatinine level of 0.5 mg/dL (44 µmol/L) or greater for patients with a baseline serum creatinine level of 1.9 mg/dL (168 µmol/L) or less, 1.0 mg/dL (88 µmol/L) or greater for patients with a baseline level of 2.0 mg/dL (177 µmol/L) to 4.9 mg/dL (433 µmol/L), and 1.5 mg/dL (133 µmol/L) or greater for patients with a baseline level greater than 5.0 mg/dL (442 µmol/L)	admission sCr level	360	120
Elrewihby, W. et al 2021[137]	Prospective	January 1 to June 30, 2019	patients above 18 years and admitted with normal renal function	Africa	KDIGO	KDIGO criteria	not reported	247	107
Fan, C. et al 2021[138]	Retrospective	2001 to 2012	patients with sepsis	North America	KDIGO	KDIGO criteria	the first measurement after admission	11008	6683

© 2023 Feng Y et al. *JAMA Network Open.*

Fan, T. et al 2021[139]	Retrospective	2001 to 2012	patients with diabetic ketoacidosis	North America	KDIGO	KDIGO criteria	sCr within 24 hours of ICU admission	532	228
Phillips, A. O.et al 2021[140]	Retrospective	April to August 2016	adult patients who attended the ED	Asian Pacific	KDIGO	KDIGO	not reported	17693	548
Haines, R. W. et al 2018[141]	Retrospective	November 1st, 2014 to May 1st, 2016	trauma admission to the emergency department	Europe	KDIGO	KDIGO	first documented sCr in hospital	830	163
He, J. et al 2018[142]	Retrospective	November 2007 to December 2016	adult inpatients	North America	KDIGO	KDIGO	the last measurement within 2-day time window prior to admission or the first sCr after admission	76957	7259
He, L. et al 2021[143]	Retrospective	February 10, 2020 to April 9,2020	patients with COVID-2019	Asian Pacific	KDIGO	KDIGO	sCr value on admission	174	20
Hectors, S. J. et al 2020[144]	Retrospective	March 9, 2020 to May 13, 2020	patients with COVID-2019	North America	KDIGO	KDIGO criteria	not reported	45	16
Hsu, C. N. et al 2020[145]	Retrospective	2010-2016	adults' hospitalization	Asian Pacific	KDIGO	KDIGO	the latest sCr or the mean sCr within 8-90 days before the index date	204064	17230
Kate, R. J. et al 2020[146]	Retrospective	2013 to 2015	inpatients over 60	North America	AKIN	AKIN criteria	not reported	44691	3786
Kate, R. J. et al 2016[147]	Retrospective	2013	inpatients over 60	North America	AKIN	AKIN criteria	not reported	25521	2258
Koyner, J. L. et al 2016[148]	Retrospective	November 2008 to January 2013	adult patients hospitalized on the wards	North America	KDIGO	KDIGO	first sCr measured on hospital admission, then updated on a rolling basis	121777	9808

© 2023 Feng Y et al. *JAMA Network Open.*

Koyner, J. L. et al 2018[149]	Retrospective	November 2008 to January 2016	all adult patients without pre-existing renal failure at admission	North America	KDIGO	KDIGO stage 1	the admission sCr value which updated on a rolling basis	72695	
Latus, J. et al 2015[150]	Retrospective	September 2012 to April 2013	patient with serologically and clinically confirmed nephropathia epidemica induced by hantavirus	Europe	RIFLE	RIFLE criteria	not reported	137	81
Li, Y. et al 2020[151]	Retrospective	October 1, 2014 to September 30, 2015	inpatients with GI cancers	Asian Pacific	KDIGO	a maximal increase in sCr by ⩾0.3 mg/dl within 48 hours, or by ⩾1.5 times baseline within the previous 7 days	the first test within 24 hours after admission	5845	837
Li, Y. et al 2020[152]	Retrospective	October 1, 2014 to September 30, 2015	patients with hematologic malignancies	Asian Pacific	KDIGO	KDIGO	first test within 24 hours after admission	2395	370
Maiwall, R. et al 2017[153]	Retrospective	October 2012 to December 2013	patients with ACLF in AARC data base	Asian Pacific	KDIGO+RRT	an increase of serum Cr ⩾ 0.3 mg/dl within 48 hours or 50% increase from the admission value at day 7 and/or requirement of RRT	the admission sCr	1363	421
Martinez, D. A. et al 2020[154]	Retrospective	January 1, 2014 to July 31, 2017	adult patients in ED	North America	KDIGO	KDIGO stage 1	sCr measured at ED arrival	91258	7209
Matheny, M. E. et al 2010[155]	Retrospective	August 1, 1999 to July 31, 2003	adult admission	North America	RIFLE	RIFLE-Injury	the average of all sCr values within 48 hours around admission	26107	726

© 2023 Feng Y et al. *JAMA Network Open.*

Patidar, K. R. et al 2019[156]	Retrospective	June 2014 to October 2018	patients with cirrhosis	North America	KDIGO	KDIGO	sCr at admission	397	59
Porter, C. J. et al 2017[157]	Retrospective	April 1, 2007 to March 31, 2011	patients admitted with hip fracture	Europe	KDIGO	KDIGO	average serum Cr level from all values measured 7-365 days pre hospitalization	1880	451
Qu, C. et al 2020[158]	Retrospective	January 2014 to January 2019	patients with acute pancreatitis	Asian Pacific	KDIGO	KDIGO	not reported	423	80
Simonov, M. et al 2019[159]	Retrospective	December 31 2012 to February 9 2016	inpatients	North America	KDIGO	KDIGO	the lowest sCr over the preceding 7 days	60701	11593
Song, X. et al 2020[160]	Retrospective	2010 to 2018	adult inpatients	North America	KDIGO	KDIGO	not reported		
Sujan, R. et al 2018[161]	Prospective	not reported	patients hospitalized with severe alcoholic hepatitis	Asian Pacific	AKIN	AKIN criteria	not reported	390	126
Wu, L. et al 2018[162]	Retrospective	November 2007 to December 2016	adult inpatients	North America	KDIGO	KDIGO stage 1	latest sCr within 2 days prior to admission or the first sCr after admission	76957	7259
Zhou, J. et al 2019[163]	Retrospective	March 2009 to June 2016	adult patients with sepsis	Asian Pacific	Self-defined	an increase of ⩾ 26.4 umol/L or ⩾ 50% baseline sCr in sCr or oliguria of <0.5 ml/kg/h for >6 h within 48 hours.	lowest sCr in 2 days prior to ICU admission or the first sCr within 2 days after ICU admission	1554	415
**VI. Prediction models of AKI in primary clinics (n=1)**									
Burckhardt, P. et al 2018[164]	Retrospective	2009 to 2013	CKD patients	North America	ICD-codes	ICD codes	not reported	1944	771

© 2023 Feng Y et al. *JAMA Network Open.*

eTable 5. Details of Model Assessment Results of Included Prediction Models

Author/Year	Predictors					Prediction Model		Performance		Validation	
	No	Prediction Time 0	Prediction Window	Overlap	Availability	Method	Detailed Method	Discrimination	Calibration	Interval	External
**I. Prediction models of AKI induced by contrast medium (n=26)**											
Ando, G. et al 2014[15]	2	at completion of the contrast use	within 72 hours after the administration of contrast medium	No	Yes	LR	stepwise multivariable regression model	AUC: 0.91 (0.86-0.95)	H-L test: x2=8.93, p=0.35	No	Yes
Brown, J. R. et al 2015[16]	39	at completion of the index surgery	48 hours after surgery to during hospitalization or within 7 days after surgery	No	Yes	ML	LASSO regression model	AUC: 0.741 (0.737-0.746)	calibration plot	Yes	Yes
Chen, Y. L. et al 2014[17]	9	at completion of PCI	within 5 days after PCI	No	Yes	LR	stepwise multivariable logistic regression model	AUC: 0.82 (0.79-0.85)	H-L test: $\chi2$ = 2.96, p = 0.89	Yes	No
Duan, C. et al 2017[18]	5	at completion of PCI	within 48 hours	No	Probably Yes	LR	logistic regression with variable selection by a bootstrap technique	AUC: 0.890 (0.749-0.970)	H-L test (x2=20.16, p=0.981)	Yes	No
Fan, P. C. et al 2018[19]	8	at completion of the index surgery	during index admission	Probably No	Probably Yes	LR	multivariable logistic regression analysis	AUC: 0.874(0.868-0.881)	not reported	Yes	No
Ghani, A. A et al 2009[20]	5	at completion of the procedure	within 48 hours after the procedure	No	Yes	LR	multivariable binary logistic regression model	not reported	Chi-square test: 5.35 p=0.37	Yes	No
Gurm, H. S. et al 2013[21]	15	at completion of the index surgery	one week following the procedure	No	Yes	LR	random forest regression	not reported	not reported	Yes	No

© 2023 Feng Y et al. *JAMA Network Open.*

Hu, X. et al 2017[22]	4	at completion of PCI	within 48-72 hours after PCI	Probab ly No	Yes	LR	logistic regression analysis	AUC: 0.913 (0.883-0.973)	H-L test	Yes	No
Huang, C. et al 2018[23]	20	at completion of the index surgery	Post procedure	No	Yes	ML	XGBoost	AUC: 0.725(0.722-0.728)	calibration slope; resolution measure (MSE between the deciles of predicted risks and the event rate of the entire cohort): 0.0004x10^-2(0.0000x10^-2-0.0009x10^-2); Brier score: 0.0630 (0.0628-0.0632)	No	No
Ibrahim, N. E. et al 2019[24]	6	at completion of coronary angiography	within 7 days after contrast exposure	No	Yes	LR	LASSO with logistic regression	AUC: 0.79, Sn:0.77; Sp: 0.75	AIC: 305.0; BIC: 338.5; H-L test:p=0.96	No	No
Inohara, T.. et al 2015[25]	7	at completion of the index surgery	within 30 days after indexed procedure	No	Yes	LR	backward stepwise multivariate logistic regression model	AUC: 0.799 (0.783-0.815)	not reported	Yes	No
Jeon, J. et al 2019[26]	3	at completion of CECT	within 2-6 days after CECT	Probab ly No	Probab ly Yes	LR	multivariate logistic regression model	AUC: 0.733 (0.657-0.810)	calibration slope: 0.867 (0.719-1.015); H-L test: x2=2.182, p=0.702	No	Yes
Ji, L. et al 2015[27]	9	at completion of PCI	within 72 hours after the use of contrast agents	No	Yes	LR	multivariate logistic regression analysis	AUC: 0.917 (0.877-0.957); Sn: 0.7917, Sp: 0.8008; PPV: 0.3502; NPV: 0.9659	H-L test: x2=128.64, p=0..3936	Yes	No
Lian, D. et al 2017[28]	3	at completion of coronary angiography	within 48-72 hours after CM exposure	No	Yes	LR	multivariate logistic regression analysis using forward	AUC: 0.727 (0.655-0.798)	risk stratification	Yes	No

© 2023 Feng Y et al. *JAMA Network Open.*

							stepwise selection				
Lin, K. Y. et al 2017[29]	4	at completion of PCI	within 72 hours of contrast exposure	Probably No	Probably Yes	LR	multivariate logistic regression analysis	AUC: 0.841 (0.744-0.939)	H-L test: x2=1.367, p=0.505	Yes	No
Liu, L. et al 2020[30]	4	at completion of PCI	within 48 to 72 hours after CAG/PCI	Probably No	Probably Yes	LR	multivariate logistic regression analysis	AUC: 0.816 (0.763-0.862)	H-L test: x2=3.65, p=0.887	Yes	No
Liu, Y. et al 2020[31]	13	at completion of PCI	within 48-72 hours after CAG or PCI	No	Yes	LR	multivariate logistic regression analysis	AUC: 0.854(0.796-0.913)	calibration plot	Yes	No
Sun, L. et al 2020[32]	15	at completion of the index surgery	within 48 hours after procedure	No	Yes	ML	random forest regression	AUC: 0.995 (0.993-0.998)	not reported	Yes	No
Tsai, T. T. et al 2014[33]	11	at completion of PCI	post PCI	No	Yes	LR	multivariate logistic regression	AUC: 0.714 (0.711-0.717)	calibration slope: 1.001	Yes	No
Yin, W. J. et al 2017[34]	13	at completion of the contrast use	72 hours after exposure to CM	No	Yes	ML	random forest	AUC: 0.907; Sp: 0.799; Sn: 0.844; Matthews correlation coefficient: 0.644	accuracy: 0.822	Yes	No
Zambetti, B. R. et al 2017[35]	4	at completion of the index surgery	3-5 days post-STEMI	No	Yes	LR	multivariate logistic regression analysis	AUC: 0.77 (0.70-0.83); Sn:0.80; Sp: 0.60	not reported	Yes	No
Zhou, X. et al 2018[36]	6	at completion of the index surgery	within 48 hours after CAG or PCI	No	Yes	LR	logistic regression	AUC: 0.775 (0.732 - 0.819)	H-L test: p=0.557; calibration plot	No	Yes
Yao Zhifeng et al 2020[37]	4	at completion of the index surgery	within 72 hours after CM exposure	No	Yes	LR	multivariate logistic regression	not reported	not reported	Yes	No
Roxana Mehran et al 2004[38]	8	at completion of the index surgery	within 48 hours after PCI	No	Yes	LR	multivariate logistic	AUC: 0.69	H-L test: x2=8.05, p=0.43	Yes	Yes

© 2023 Feng Y et al. *JAMA Network Open.*

							regression analysis				
Guiseppe Ando et al 2012[39]	3	at completion of CM use	within 72 hours after contrast medium	No	Yes	LR	logistic regression	AUC: 0.88 (0.85 - 0.91)	not reported	No	No
Dimitrios Tziakas et al 2011[40]	5	at completion of the index surgery	within 48 hours after PCI	No	Yes	LR	multivariate logistic regression analysis	AUC: 0.759 (0.719 - 0.797)	H-L test: x2=5.79, p>0.05; calibration plot	Yes	Yes
**II. Prediction models of AKI induced by agents other than contrast medium (n=3)**											
Jeon, N. et al 2019[41]	27	on the day when any nephrotoxic medication was given	within 4 days after admission	Probably No	Probably Yes	LR	multivariate logistic regression analysis	AUC: 0.787(0.767-0.807)	not reported	Yes	No
Motwani, S. S. et al 2018[42]	4	the initial cisplatin administration	within 14 days after the index date	Probably No	Probably Yes	LR	multivariate logistic regression with backward elimination	AUC: 0.72 (0.70-0.76)	H-L: p=0.16	No	Yes
Xu, N. et al 2020[43]	3	the day of the initial therapeutic drug monitoring	during the period of vancomycin treatment	Probably No	Probably Yes	LR	multivariate logistic regression	AUC: 0.793 (0.732-0.855)	H-L test: x2=6.079, p=0.638	Yes	Yes
**III. Prediction models of post-operative AKI (n=64)**											
STARSurg Collaborative , 2018[44]	6	at completion of the index surgery	within 7 days of the index surgery	No	Yes	LR	Bootstrap stability	AUC:0.66 (0.64-0.68)	H-L test: p=0.571, together with the size of differences between observed and predicted risk in deciles of risk	Yes	No
Adhikari et al, 2019[45]	69	at completion of the index surgery	during first 7 days after surgery,	No	Probably Yes	ML	machine learning	AUC: 0.84 (0.82-0.87)	not reported	Yes	No
Al-Jefri et al, 2020[46]	16	at completion of the index surgery	AKI on the next day while a patient is in hospital after the index surgery	No	Yes	ML	logistic regression (LR)	AUC: 0.69; sensitivity 0.67,		Yes	No

© 2023 Feng Y et al. *JAMA Network Open.*

								specificity 0.72			
Antunes et al, 2009[47]	2	at completion of the index surgery	post-op during hospitalization	No	Yes	LR	multivariable regression model	AUC: 0.778(0.738-0.818)	H-L test: x2=11.692,p=0.165; calibration curve	Yes	No
Bell et al, 2015[48]	7	at completion of the index surgery	during the first postoperative week	No	Yes	LR	multivariable logistic regression analysis	AUC: 0.74 (0.73-0.75)	calibration slop: 1	Yes	Yes
Berg et al, 2013[49]	11	at completion of the index surgery	after surgery during hospitalization	No	Probably Yes	LR	Logistic regression with limited backwards elimination	AUC: 0.819 (0.801-0.837)	H-L test: p=0.17; calibration curve	Yes	No
Bihorac et al, 2019[50]	285	at completion of the index surgery	during hospitalization after the index surgery	No	Yes	ML	automated EHR algorithm	AUC:0.80 (0.79-0.80)	H-L test	Yes	No
Birnie et al, 2014[51]	15	at completion of the index surgery	within 7 days	No	Yes	LR	multivariable logistic regression analysis	AUC: 0.73 (0.72-0.74)	H-L test: P=0.490	No	Yes
Che et al, 2019[52]	7	at completion of the index surgery	within 48 hours to 7 days after the index surgery	Probably Yes	Probably No	LR	multivariable logistic regression analysis	AUC: 0.82 (95% CI 0.78–0.85)	H-L: x2=5.73, p=0.220	No	Yes
Chen et al, 2020[53]	3	at completion of the index surgery	48 hours after surgery	Probably Yes	Probably No	LR	binary logistic regression with LASSO selection	AUC: 0.87 (0.80-0.94)	Akaike Information Criterion (AIC): 142.78; Bayesian Information Criterion (BIC): 156.05; Brier's score:0.10	No	Yes
Coulson et al, 2021[54]	5	at the completion of surgery	postoperative	No	Yes	LR	restrictive stepwise model	0.68	calibration plot	Yes	No
Mehmet et al, 2020[55]	6	at completion of the index surgery	within 3 days after surgery	No	Yes	LR	multivariable logistic regression analysis	AUC:0.803	not reported	No	No

© 2023 Feng Y et al. *JAMA Network Open.*

Du et al, 2021[56]	11	at completion of the index surgery	within 48 hours of operation	Probably No	Probably Yes	LR	logistic regression with LOSSO feature selection	AUC: 0.967(0.934-0.985)	not reported	Yes	No
Grimm et al, 2015[57]	15	at completion of the index surgery	after lung transplantation	Probably Yes	Probably Yes	LR	multivariable logistic regression model in forward and backward fashion	AUC: 0.71	calibration plot	Yes	No
Guan et al, 2019[58]	14	at completion of the index surgery	within 7 days after surgery	No	Yes	LR	multiple logistic regression analysis	AUC: 0.796 (0.795-0.797)	calibration plot	Yes	No
Hofer et al, 2020[59]	52	at completion of the index surgery	between the end of the surgery and hospital discharge	Probably No	Probably Yes	ML	deep neural network	AUC: 0.792 (0.775-0.808), Sn: 0.548(0.515-0.579), Sp: 0.881(0.87-0.892)	F1 score: 0.559(0.533-0.587)	No	No
Hu et al, 2021[60]	7	at completion of the index surgery	within 7 days after surgery	Probably No	Yes	LR	logistic regression with LASSO feature selection	AUC: 0.797 (0.762-0.835)	H-L test: p=0.835; calibration curve	Yes	No
Hu et al, 2020[61]	3	at completion of the index surgery	within 7 days after surgery	Yes	Probably No	LR	multivariate logistic regression analysis	AUC: 0.78(0.71, 0.84)	calibration curve	No	Yes
Jiang et al, 2016[62]	5	preoperative	within 48 hours after the index surgery	Probably Yes	Probably No	LR	multivariate logistic regression analysis	AUC: 0.75	H-L test: p=0.569	No	Yes
Jorge-Monjas et al, 2016[63]	4	at ICU admission	during ICU admission	Probably No	Probably Yes	LR	multivariate logistic regression analysis	AUC: 0.89 (0.85-0.92)	H-L test: x2=YESYES.87, p=0.YES6	No	Yes

© 2023 Feng Y et al. *JAMA Network Open.*

Kalisvaart et al, 2019[64]	5	at completion of the index surgery	within the first 7 postoperative days	Probably No	Probably Yes	LR	multivariate logistic regression with backward stepwise approach	AUC: 0.70	H-L test: p=0.664	Yes	No
Kashani et al, 2015[65]	4	preoperative	after surgery	Probably No	Probably Yes	LR	multivariate logistic regression with backward stepwise approach	AUC: 0.669 (0.628-0.710)	H-L test: not reported	No	No
Kim et al, 2014[66]	5	at completion of the index surgery	after transplantation	No	Yes	LR	multivariate logistic regression	AUC: 0.90 (0.85-0.95)	calibration plot	No	No
Kim et al, 2011[67]	3	at completion of the index surgery	within 48 hours after surgery	No	Yes	LR	multivariate logistic regression	AUC: 0.73 (0.64-0.83)	not reported	No	No
Ko et al, 2020[68, 69]	6	at completion of the index surgery	during the first postoperative week	No	Yes	ML	gradient boosting machine	AUC: 0.78(0.74-0.81), Sn:0.65, Sp: 0.77	calibration plot	No	Yes
Lee et al, 2018[69]	72	at completion of the index surgery	the first 7 postoperative days	Probably No	Probably Yes	ML	XGBoost gradient boosting model	AUC: 0.78(0.75-0.80)	test error rate: 0.26	Yes	No
Lee et al, 2018[70]	20	at completion of the index surgery	during first two postoperative days	No	Yes	ML	gradient boosting machine (GBM)	AUC: 0.90 (0.86-0.93)	not reported	Yes	No
Legrand et al, 2013[71]	33	at completion of the index surgery	during the first 7 days after surgery	No	Yes	LR	stepwise regression AIC	AUC: 0.757 (0.689-0.826)	mean squared prediction error	No	No
Lei et al, 2020[72]		at completion of the index surgery	within 7 days after surgery	Probably No	Probably Yes	ML	ranom forst regression	AUC: 0.989± 0.011, precision: 0.979,	recall: 0.852, FYES-score: 0.9YESYES	Yes	No

© 2023 Feng Y et al. *JAMA Network Open.*

								accuracy: 0.989			
Lei et al, 2019[73]		at completion of the index surgery	within 7 days after surgery	No	Yes	ML	gradient boosting machines	not reported	not reported	Yes	No
Li et al, 2020[74]	12	at completion of the index surgery	within 7 days	Probab ly Yes	Probab ly No	ML	Bayesian Network	AUC: 0.755 (0.738-0.771)	not reported	Yes	Yes
Lin et al, 2020[75]	7	at completion of the index surgery	within 7 days after surgery	Probab ly No	Probab ly Yes	LR	multivariate regression analysis	AUC: 0.738	calibration plot	No	Yes
Liu et al, 2016[76]	13	at completion of the index surgery, within the first 7 days of hospitalization	within the first 7 days of hospitalization	Probab ly No	Probab ly Yes	LR	stepwise multivariate logistic regression with Fisher's linear discriminant analysis for feature selection	AUC: 0.89 (0.88-0.90)	AIC: 65YES5.93	Yes	No
Martini et al, 2019[77]	6	at completion of the index surgery	postoperative to discharge	No	Yes	LR	multivariate logistic regression analysis	AUC: 0.74(0.71-0.78)	calibration plot	Yes	No
McBride et al, 2019[78]	6	at completion of the index surgery	postoperative	Probab ly Yes	Probab ly No	LR	logistic regression	AUC: 0.836 (0.785 – 0.888)	not reported	No	No
Metzger et al, 2016[79]		at completion of the index surgery	not reported	No	Yes	LR	logistic regression analysis	AUC: 0.81 (0.72-0.88)	not reported	No	No
Meyer et al, 2018[80]	52	at completion of the index surgery	within the first 24 hours after the initial surgery	Probab ly No	Probab ly Yes	ML	RNN	0.91	not reported	Yes	Yes
Nah et al, 2016[81]	8	at completion of the index surgery	within the first 48 hours postoperatively	No	Yes	ML	multivariate backward step-wise logistic regression	AUC: 0.80 (0.68-0.82)	H-L test: p=0.33	No	Yes

© 2023 Feng Y et al. *JAMA Network Open.*

Ortega-Loubon et al, 2018[82]	6	at completion of the index surgery	after cardiac surgery	No	Yes	LR	multiple logistic regression analysis with stepwise selection	AUC: 0.783 (0.713-0.854)	H-L test: p=0.720	Yes	No
Pannu et al, 2016[83]	8	at completion of the index surgery	YES4 days after cardiac surgery	No	Yes	LR	multivariate logistic regression with bootstrapping selection	AUC: 0.87 (0.85-0.90)	H-L test: p=0.7; calibration slope: 0.96	No	Yes
Paparella et al, 2014[84]	6	at completion of the index surgery	postoperative during hospitalization	No	Yes	LR	multiple logistic regression analysis with stepwise selection	AUC: 0.844	H-L test: p=0.799	No	Yes
Penny-Dimri et al, 2020[85]	56	at completion of the index surgery	during admission	Probably No	Probably Yes	ML	gradient boosted machine	AUC: 0.78± 0.01; Sn: 0.73; Sp: 0.70	not reported	Yes	No
Ramonell et al, 2016[86]	9	at completion of the index surgery	during admission	No	Yes	LR	multivariate logistic regression	AUC: 0.79	not reported	No	Yes
Rank et al, 2020[87]	96	at completion of the index surgery	within 7 days after cardiothoracic surgrey	Probably No	Probably Yes	ML	RNN	AUC: 0.90	H-L test: p=0.37; calibration plot	Yes	No
Rueggeberg et al, 2008[88]	6	at completion of the index surgery	postoperative	No	Yes	LR	forward stepwise binary logistic regression analysis	AUC: 0.914	H-L test: x2=YES.2, p=0.997	No	Yes
Simonini et al, 2014[89]	9	at completion of the index surgery	after surgery	No	Yes	LR	logistic regression analysis	AUC: 0.84 (0.79-0.88)	not reported	No	No
Slankamenac et al, 2013[90]	4	at completion of the index surgery	within 48 hours after surgery	No	Yes	LR	stepwise backward logistic	AUC: 0.81 (0.76-0.86)	calibration plot; H-L test: p=0.93	Yes	No

© 2023 Feng Y et al. *JAMA Network Open.*

							regression model				
Slankamenac et al, 2009[91]	4	at completion of the index surgery	within 48 hours after surgery	No	Yes	LR	multivariate logistic regression model	AUC: 0.80	not reported	Yes	No
Thakar et al, 2005[92]	13	at completion of the index surgery	postoperative period	No	Yes	LR	logistic regression	AUC: 0.81 (0.78-0.83)	not reported	Yes	No
Tian et al, 2021[93]	20	at completion of the index surgery	postoperative period	No	Yes	LR	multivariate logistic regression analysis	AUC: 0.69 (0.68-0.69)	H-L test: p=0.95	Yes	No
Trongtrakul et al, 2020[94]	6	at ICU admission	within the first 7 days of ICU admission	No	Yes	LR	multivariate logistic regression	AUC: 0.839 (0.825-0.852)	H-L test: p=0.302; calibration plot	Yes	No
Tseng et al, 2020[95]	94	at completion of the index surgery	first 7 days postoperative	No	Yes	ML	random forest + Xgboost	not reported	not reported	Yes	No
Xue et al, 2021[96]	711	at completion of the index surgery	post operation	No	Yes	ML	gradient boosting tree	AUC: 0.848 (0.846 - 0.851)	not reported	Yes	No
Yayac et al, 2021[97]	41	at completion of the index surgery	postoperative day YES	No	Yes	ML	stochastic gradient boosting regression model	AUC: 0.967 (0.96 - 0.98); Sn: 0.961; Sp=0.959; precision: 0.487	not reported	No	No
Zhang et al, 2020[98]	5	at completion of the index surgery	within 7 days after CM exposure	No	Yes	LR	multivariate logistic regression analysis with forward stepwise feature selection	AUC: 0.796 (0.795 - 0.797)	calibration plot	Yes	No
Zhou et al, 2020[99]	7	at completion of the index surgery	post operation	Probably No	Probably Yes	ML	random forest	AUC: 0.89± 0.08	not reported	Yes	No

© 2023 Feng Y et al. *JAMA Network Open.*

Jeremiah et al, 2013[100]	8	at completion of the index surgery	after surgery	No	Yes	LR	multivariate logistic regression analysis	AUC: 0.72 (0.68-0.75)	H-L test: x2=8.68, p=0.28; calibration plot	No	No
Rahendra et al, 2006[101]	10	at completion of the index surgery	after surgery	No	Yes	LR	logistic regression	AUC: 0.75	not reported	No	Yes
Duminda et al, 2007[102]	7	at completion of the index surgery	after surgery	No	Yes	LR	logistic regression	AUCL 0.81 (0.78 - 0.84)	H-L test: p=0.27	No	Yes
Paloma et al, 2007[103]	8	at completion of the index surgery	during hospitalization	No	Yes	LR	multivariate logistic regression analysis	AUC: 0.843 (0.78 - 0.89)	H-L test: p=0.803	No	Yes
Sevag et al, 2012[104]	12	preoperative	within 2 weeks after cardiac surgery	No	Yes	LR	multivariate logistic regression analysis using backward selection	AUC: 0.875 (0.859 - 0.891)	H-L test: p=0.2	Yes	No
Sachin et al, 2009[105]	9	at completion of the index surgery	within 30 days after surgery	No	Yes	LR	multivariate logistic regression	AUC: 0.80± 0.01	not reported	Yes	No
Ng et al, 2014[106]	17	at completion of the index surgery	within 30 days after surgery	Probably Yes	Probably No	LR	logistic regression	AUC: 0.81	H-L test: p=0.6	No	No
Kim et al, 2013[107]	6	at completion of the index surgery	within the first 7 days after surgery	No	Yes	LR	multivariate logistic regression analysis	AUC: 0.84 (0.69 - 0.89)	not reported	Yes	No
**IV. Prediction models of AKI in ICU (n=21)**											
An, S. et al 2020[108]	11	at hospital admission	during hospitalization	Probably Yes	Probably No	LR	Least absolute shrinkage and selection operator (LASSO) regularization was used for variable	AUC: 0.8786	H-L test: x2=4.4175, p=0.8176;calibration curve	No	No

© 2023 Feng Y et al. *JAMA Network Open.*

							selection, and logistic regression for modelling				
Asada, T. et al 2016[109]	5	at ICU admission	during ICU hospitalization	No	Probably Yes	LR	multivariable logistic regression analysis	AUC:0.940(0.793-0.985)	not reported	No	No
Bhatraju, P. K. et al 2019[110]	3	at ICU admission	within 72 hours after study enrollment	Probably Yes	Probably No	LR	combined specific variables	AUC: 0.95 (0.91-0.97); sensitivity:0.47 (0.35 to 0.58); specificity:0.99 (0.98 to 0.99); PPV:0.83 (0.71 to 0.94); NPV:0.95 (0.94 to 0.96)		Yes	Yes
Chen, Y. et al 2019[111]	5	at ICU admission	within 48 hours to 7 days	Probably Yes	Probably No	LR	binary logistic regression	AUC: 0.81 (95% CI 0.78-0.85)	H-L test: p=0.588	No	No
Chiofolo, C. et al 2019[112]	19	at ICU admission	from ICU admission to discharge	Probably Yes	Probably No	ML	random forest classification and logistic regression	AUC 0.913(0.904-0.922); sensitivity: 98%, specificity:71%, PPV: 45%, NPV: 99%, PLR 3.3, NLR: 0.0	H-L test: p=0.3	Yes	No
Deng, F. et al 2020[113]	7	at ICU admission	first 24 hours of ICU admission	Probably Yes	Probably No	LR	stepwise logistic regression	AUC: 0.80 (0.78-0.82)	calibration plot	Yes	No
Deng, Y. et al 2017[114]	2	at ICU admission	within 1 week after ICU admission	Probably Yes	Probably No	LR	multivariable logistic regression	AUC: 0.756(0.723-0.789),	not reported	Yes	No

© 2023 Feng Y et al. *JAMA Network Open.*

								sensitivity: 0.49, specificity: 0.91, positive likelihood ratio(+)LR: 5.69, negative likelihood ratio(-)LR: 0.56, PPV: 0.71, NPV: 0.81			
Deng, Y. et al 2020[115]	5	at ICU admission	within 72 hours after surgery	Probably Yes	Probably No	LR	multivariable logistic regression analysis	AUC: 0.808(0.756-0.861), IDI: 0.076(0.035-0.117), cNRI: 0.633(0.386-0.880)	not reported	Yes	No
Ferrari, F. et al 2019[116]	5	at ICU admission	during ICU stay	Probably Yes	Probably No	LR	multivariable logistic regression with stepwise forward selection	AUC: 0.73 (0.68-0.78); Se=0.574, Sp=0.80	H-L test: p=0.5232; IDI=0.012	Yes	No
Ferrari, F. et al 2019[117]	2	at ICU admission	within 48 hours after ICU admission	Probably Yes	Probably No	LR	multivariable logistic regression	AUC: 0.70 (0.65-0.76)	H-L test	No	No
Flechet, M. et al 2017[118]	13	at ICU admission	during ICU stay	Probably Yes	Probably No	ML	random forest machine-learning algorithm	AUC:0.86(0.86-0.86), Sn:0.78(0.78-0.78), Sp:0.81 (0.80-0.81)	calibration slop: 0.87 (0.87-0.88); calibration in-the-large: -0.00 (-0.01- -0.00)	Yes	No
Malhotra, R. et al 2017[119]	10	at ICU admission	within 7 days after study enrollment	Probably No	Probably Yes	LR	multivariate logistic regression analysis	not reported	not reported	Yes	Yes

© 2023 Feng Y et al. *JAMA Network Open.*

Matsuura, R. et al 2020[120]	4	72 hours after ICU admission	within 7 days of ICU admission	No	Probably Yes	LR	multivariate logistic regression analysis	AUC: 0.79(0.77 to 0.81)	not reported	Yes	No
Mohamadlou, H. et al 2018[121]		at 12, 24, 48, and 72 hours after admission	within 72 hours	No	Yes	ML	XGBoost	AUC: BIDMC: 0.674 (0.669-0.679); Sn=0.82, Sp=0.45, accuracy: 0.80; Stanford: 0.728 (0.719-0.737); Sn=0.78, Sp=0.53, accuracy: 0.79	not reported	No	No
Nusshag, C. et al 2019[122]	3	at study inclusion	within 7 days after study inclusion	Probably No	Probably Yes	LR	logistic regression	AUC: 0.93 (0.86-1.00)	not reported	No	No
Parreco, J. et al 2019[123]		at 48 hours after ICU admission	during ICU admission	Probably No	Probably Yes	ML	gradient boosted trees	AUC: 0.834± 0.006	accuracy: 0.9394, F-measure: 0.4296	Yes	No
Wang, Q. et al 2019[124]	10	at ICU admission	7 days after ICU admission	Probably Yes	Probably No	LR	multivariate logistic regression	AUC: 0.833 (0.802-0.864)	calibration plot, H-L test: p=0.511	Yes	No
Wang, Y. et al 2020[125]		at 24 hours after ICU admission	within 48 hours after a given prediction time point during ICU admission, dynamically	Probably No	Probably Yes	ML	ensemble learning and time series model	AUC: 0.806± 0.002	F1 score: 0.44	Yes	No
Wiersema, R. et al 2020[126]	3	at ICU admission	during ICU admission	Probably No	Probably Yes	LR	logistic regression model	AUC: 0.70 (0.66-0.74)	H-L test: x2=10.67, p=0.22	No	No
Xie, Y. et al 2021[127]	3	at ICU admission	during ICU admission	Probably Yes	Probably No	LR	multivariate logistic regression	AUC: 0.9862	H-L test: p=0.000	Yes	Yes

© 2023 Feng Y et al. *JAMA Network Open.*

Zimmerman, L. P. et al 2019[128]	16	at ICU admission	day 2 and day 3 of ICU admission	No	Yes	LR	logistic regression	AUC: 0.783, Sn: 0.698; Sp: 0.736; PPV: 0.342; NPV: 0.925	accuracy: 0.729	No	No
**V. Prediction models of AKI in general hospitalizations (n=35)**											
RISK Investigators et al 2019[129]	4	24 hours after hospital admission	>24 hours after hospital admission	No	Probably Yes	LR	binary logistic regression ans a backwards stepwise procedure	AUC:0.75 (0.70-0.80)	H-L test: P=0.14,x2=5.48	No	No
Abusaada, K. et al 2017[130]	7	at hospital admission	during hospitalization	Probably No	Probably No	LR	backward elimination logistic regression	AUC:0.76 (95 % CI, 0.72–0.80)	H-L test: x2=0.69; NRI: 0.2%	Yes	No
Argyropoulos, A. et al 2019[131]	3	at hospital admission	within 7 days of admission	Probably Yes	Probably No	LR	multivariable logistic regression (MLR) model	AUC: 0.70(0.61-0.79)	not reported	Yes	Yes
Bihorac, A. et al 2013[132]	10	at hospital admission	post-traumatic during the first 28 days of hospitalization	Probably Yes	Probably No	LR	multivariable logistic regression analysis	AUC: 0.83(0.76-0.91)	not reported	No	No
Breidthardt, T. et al 2012[133]	2	at ED presentation	during the first 48 hours after ED presentation	No	Probably No	LR	combined logistic regression model	AUC: 0.82 (95% CI 0.74-0.89)	not reported	No	No
Cheng, P. et al 2017[134]		at hospital admission	during hospitalization	No	Yes	ML	random forest	AUC: 0.751	precision: 0.662; recall: 0.736	Yes	No
Cronin, R. M. et al 2015[135]	3	48 hours after hospital admission	7-day period in the post-admission time window (+48 h to +9 days)	No	Yes	LR	least absolute shrinkage and selection operator (lasso) regression	not reported	not reported	Yes	No

© 2023 Feng Y et al. *JAMA Network Open.*

Drawz, P. E. et al 2008[136]	7	at hospital admission	during hospitalization	Probably Yes	Probably No	LR	multivariable logistic regression analysis	AUC: 0.73	not reported	Yes	No
Elrewihby, W. et al 2021[137]	8	at admission	within 24 hours after admission	Probably Yes	Probably No	LR	multivariable logistic regression analysis	AUC: 0.950(0.922-0.978), sensitivity: 94.39%, specificity: 81.43%, PPV: 79.5%, NPV: 95.0%	not reported	No	Yes
Fan, C. et al 2021[138]	10	at the first measurement after ICU admission	within 48 hours after ICU admission	Probably No	Probably No	LR	multivariable logistic regression analysis with variables selected by LASSO method	AUC: 0.711(0.702-0.721), Sn:0.744, Sp: 0.534, NPV: 0.568, PPV: 0.723	calibration curve	Yes	No
Fan, T. et al 2021[139]	8	at ICU admission	during ICU stay	Probably Yes	Probably No	LR	multivariable logistic regression analysis based on variables derived from LASSO regression	AUC: 0.747(0.706-0.789); Sn: 0.838, Sp: 0.559, PPV: 0.588, NPV: 0.821	H-L test: x2=4.885, p=0.844; calibration plot	Yes	No
Phillips, A. O.et al 2021[140]	8	at ED presentation	in the ED prior to discharge or admission	No	Yes	LR	multivariable Cox proportional hazard modelling	AUC:0.793 (0.774-0.822)	H-L test: x2=27.427, p=0.0006	Yes	No
Haines, R. W. et al 2018[141]	4	at ICU admission	within 7 days of major trauma requiring ICU admission	Probably Yes	Probably No	LR	logistic regression analysis	AUC: 0.77(0.72-0.81)	unweighted sum of squares test and calibration plot	Yes	No

© 2023 Feng Y et al. *JAMA Network Open.*

He, J. et al 2018[142]	1917	at hospital admission	during admission	Probably Yes	Probably No	ML	Naïve Bayes	AUC: 0.687(0.686-0.687)	F-measure: 0.261(0.260-0.262)	No	No
He, L. et al 2021[143]	4	at hospital admission	within 7 days	Probably Yes	Probably No	LR	multivariable logistic regression model with LASSO feature selection	AUC: 0.955(0.916-0.955); Sn:0.95; Sp:0.851	accuracy: 0.954	Yes	No
Hectors, S. J. et al 2020[144]	3	at hospital admission	within 7 days	Probably No	Probably Yes	LR	multivariate logistic regression analysis with stepwise feature selection	AUC: 0.89 (0.78-1); Sn=0.875, Sp: 0.828	not reported	No	Yes
Hsu, C. N. et al 2020[145]	10	at hospital admission	within 48 hours	No	Yes	ML	logistic regression model with XGBoost feature selection	AUC: 0.7670 (0.7608-0.7732); Sn: 0.6142 (0.5855-0.6431); Sp: 0.7848 (0.7529-0.8167)	not reported	Yes	No
Kate, R. J. et al 2020[146]	33	each day, dynamically	within 48 hours, dynamically	Probably No	Probably Yes	ML	not reported	AUC: 0.709 (0.690-0.728)	not reported	No	No
Kate, R. J. et al 2016[147]		24 hours after hospital admission	from 24 hours after admission to discharge	Probably No	Probably Yes	ML	an ensemble of LR, SVM, Decision trees and naïve Bayes	AUC: 0.664 (0.651-0.676)	not reported	No	No
Koyner, J. L. et al 2016[148]	29	every 12 hours during ward hospitalization	within each future 24 hours, dynamically	Probably No	Probably Yes	LR	derived discrete time logistic regression	AUC: 0.74 (0.74-0.74)	not reported	Yes	No
Koyner, J. L. et al 2018[149]	20	at admission	within each future 48 hours	No	Yes	ML	gradient boosting machine	AUC: 0.73 (0.72-0.73)	calibration plot	Yes	No

© 2023 Feng Y et al. *JAMA Network Open.*

Latus, J. et al 2015[150]	3	during the course of disease	during hospitalization	Probably No	Probably Yes	LR	multivariate logistic regression	AUC: 0.706(0.676-0.737)	not reported	Yes	No
Li, Y. et al 2020[151]	11	within the first 7 days after admission, possibly after the first test	within the previous 7 days	Probably No	Probably Yes	ML	Bayesian Network	AUC: 0.823 (0.809-0.837)	Accuracy: 0.870, recall: 0.265, PPV: 0.607, NPV:0.888	Yes	No
Li, Y. et al 2020[152]	11	at hospital admission, possibly after the first test	within 7 days	Probably No	Probably Yes	ML	Bayesian Network	AUC: 0.835 (0.812-0.858)	not reported	No	No
Maiwall, R. et al 2017[153]	7	at day 7 after admission	at day 7 after admission	Probably No	Probably Yes	LR	multivariate logistic regression analysis	not reported	not reported	Yes	Yes
Martinez, D. A. et al 2020[154]		at 24, 48 and 72 hours after ED presentation	within 24, 48 and 72 hours	No	Yes	ML	LASSO feature selection and random forest regression	AUC: 0.74-0.80, Sn: 0.69-0.72, Sp:0.67-0.73	not reported	No	No
Matheny, M. E. et al 2010[155]	23	24 hours after hospital admission	after the first 24 hours of admission and up to 30 days of hospitalization	No	Yes	LR	logistic regression	AUC: 0.78 (0.76-0.79)	H-L test: x2=12.7, p=0.12; risk stratification	Yes	No
Patidar, K. R. et al 2019[156]	3	at hospital admission	within 7 days after admission	Probably Yes	Probably No	LR	multivariate logistic regression analysis	AUC: 0.77 (0.70-0.83)	not reported	No	Yes
Porter, C. J. et al 2017[157]	4	at hospital admission	not reported	Probably No	Probably Yes	ML	logistic regression	AUC: 0.63 (0.59-0.67)	H-L test: p=0.464; calibration plot	Yes	No
Qu, C. et al 2020[158]	5	at hospital admission	during hospitalization	Probably Yes	Probably No	ML	XGBoost (extreme gradient boosting)	AUC: 0.9193	not reported	Yes	No
Simonov, M. et al 2019[159]	35	at 24 hours after hospital admission	within 24 hours after each given observation, dynamically	Probably No	Probably Yes	LR	discrete-time logistic regression approach	AUC: 0.74 (0.73-0.74)	not reported	Yes	Yes

© 2023 Feng Y et al. *JAMA Network Open.*

Song, X. et al 2020[160]	300 0	at 48 hours after hospital admission	within 48 hours after a given prediction time point, dynamically	Probab ly No	Probab ly Yes	ML	Gradient Boosting Tree-based Machines (GBT)	AUC: 0.76 (0.75-0.78)	H-L test: x2=42.8, p=0.01	Yes	Yes
Sujan, R. et al 2018[161]	3	at hospital admission	within hospitalization	Probab ly No	Probab ly Yes	LR	multivariate logistic regression with backward elimination	AUC: 0.74 (0.69-0.80)	H-L test: x2=12.7	Yes	No
Wu, L. et al 2018[162]	NR	at hospital admission	during admission	Probab ly No	Probab ly Yes	ML	GBM	AUC: 0.76 (0.75-0.76)	not reported	Yes	No
Zhou, J. et al 2019[163]	16	at ICU admission	within 48 hours	Probab ly Yes	Probab ly No	LR	stepwise forward logistic regression analysis	AUC: 0.861 (0.840 - 0.878)	H-L test: x2=6.23, p=0.61	Yes	No
**VI. Prediction models of AKI in primary clinics (n=1)**											
Burckhardt, P. et al 2018[164]	11	during study period	study period (2009 to 2013)	Probab ly Yes	Probab ly No	LR	multi-trajectory modelling	AUC: 0.692	not reported	No	No

© 2023 Feng Y et al. *JAMA Network Open.*

eTable 6. Pooled C Statistics, Sensitivities, and Specificities From sROC Analysis

Subgroups	C statistic (95% CI)	Sensitivity	Specificity
All studies (n=103)	0.84 (0.80 - 0.87)	0.76 (0.73 - 0.79)	0.78 (0.75 - 0.80)
CM associated AKI (n=22)	0.83 (0.79 - 0.86)	0.78 (0.73 - 0.82)	0.75 (0.71 - 0.78)
post-operative AKI (n=42)	0.83 (0.80 - 0.86)	0.75 (0.71 - 0.78)	0.78 (0.75 - 0.81)
AKI in ICU (n=17)	0.88 (0.84 - 0.90)	0.79 (0.68 - 0.87)	0.82 (0.75 - 0.87)
AKI in general hospitalizations (n=22)	0.82 (0.78 - 0.85)	0.75 (0.69 - 0.79)	0.76 (0.71 - 0.80)

Abbreviations: AKI, acute kidney injury; CI, confidence interval; CM, contrast medium; ICU, intensive care unit.

© 2023 Feng Y et al. *JAMA Network Open.*

eTable 7. Results of Subgroup Analysis for Potential Significant Sources of High Heterogeneities

Parameter	Sensitivity	P1	Specificity	P2
ROB	0.70 [0.58-0.79]	0.24	0.76 [0.67-0.83]	0.61
Study Design	0.78 [0.70-0.85]	0.58	0.80 [0.75-0.85]	0.38
Region	0.76 [0.72-0.80]	0.90	0.78 [0.75-0.81]	0.93
AKI Definition	0.74 [0.70-0.78]	0.42	0.77 [0.74-0.80]	0.65
Overlap	0.80 [0.73-0.85]	0.29	0.80 [0.75-0.84]	0.41
Predictor Availability	0.71 [0.63-0.78]	0.25	0.76 [0.70-0.81]	0.58
Development Methods	0.77 [0.71-0.83]	0.70	0.79 [0.74-0.83]	0.71
Frequency	0.76 [0.73-0.79]	0.97	0.78 [0.76-0.80]	0.98

Abbreviations: AKI, acute kidney injury; P1, p value for pooled sensitivity; P2, p value for pooled specificity; ROB, risk of bias.

© 2023 Feng Y et al. *JAMA Network Open.*

eFigure 1. Forest Plot for Meta-analysis of C Statistics of Contrast Medium Associated AKI Prediction Models

Study	TE	SE	Weight	Std. Mean Difference IV, Random, 95% CI
Ando 2014	0.91	0.0260	3.9%	0.91 [0.86; 0.96]
Brown 2015	0.74	0.0020	4.3%	0.74 [0.74; 0.74]
Chen 2014	0.82	0.0150	4.2%	0.82 [0.79; 0.85]
Duan 2017	0.89	0.0720	2.4%	0.89 [0.75; 1.03]
Fan 2018	0.87	0.0030	4.3%	0.87 [0.87; 0.88]
Ghani 2009	0.61	0.0800	2.2%	0.61 [0.45; 0.77]
Gurm 2013	0.84	0.0090	4.3%	0.84 [0.82; 0.86]
Hu 2017	0.93	0.0263	3.9%	0.93 [0.88; 0.98]
Huang 2018	0.72	0.0020	4.3%	0.72 [0.72; 0.73]
Ibrahim 2019	0.79	0.0353	3.6%	0.79 [0.72; 0.86]
Inohara 2015	0.80	0.0080	4.3%	0.80 [0.78; 0.81]
Jeon 2019	0.73	0.0390	3.5%	0.73 [0.66; 0.81]
Ji 2015	0.92	0.0188	4.1%	0.92 [0.88; 0.95]
Lian 2017	0.73	0.0370	3.6%	0.73 [0.65; 0.80]
Lin 2017	0.84	0.0490	3.2%	0.84 [0.74; 0.94]
Liu 2020	0.82	0.0270	3.9%	0.82 [0.76; 0.87]
Liu 2020	0.85	0.0300	3.8%	0.85 [0.80; 0.91]
Sun 2020	1.00	0.0010	4.3%	1.00 [1.00; 1.00]
Tsai 2014	0.71	0.0020	4.3%	0.71 [0.71; 0.72]
Yin 2017	0.91	0.0054	4.3%	0.91 [0.90; 0.92]
Zambetti 2017	0.77	0.0360	3.6%	0.77 [0.70; 0.84]
Zhou 2018	0.78	0.0220	4.0%	0.78 [0.73; 0.82]
Yao 2020	0.76	0.0460	3.3%	0.76 [0.67; 0.85]
Roxana 2004	0.69	0.0104	4.2%	0.69 [0.67; 0.71]
Guiseppe 2012	0.88	0.0150	4.2%	0.88 [0.85; 0.91]
Dimitrios 2011	0.76	0.0200	4.1%	0.76 [0.72; 0.80]
**Total (95% CI)**			**100.0%**	**0.81 [0.78; 0.85]**
**Prediction interval**				**[0.64; 0.99]**

Heterogeneity: Tau^2 = 0.0067; Chi^2 = 32765.67, df = 25 (P = 0); I^2 = 100%

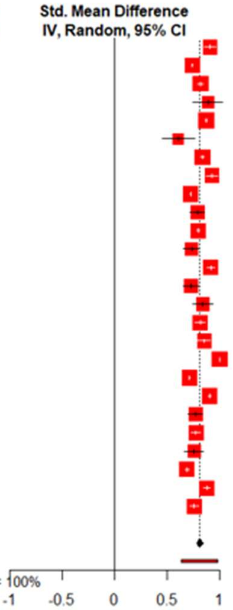

Note: The red line indicates 95% prediction interval, which is wider than 95% CI.

© 2023 Feng Y et al. *JAMA Network Open.*

eFigure 2. Forest Plot for Meta-analysis of C Statistics of Postoperative AKI Prediction Models

Study	TE	SE	Weight	Std. Mean Difference IV, Random, 95% CI
STARSurg Collaborative 2018	0.66	0.0100	1.7%	0.66 [0.64; 0.68]
Adhikari 2019	0.84	0.0100	1.7%	0.84 [0.82; 0.86]
Al-Jefri 2020	0.69	0.0052	1.7%	0.69 [0.68; 0.70]
Antunes 2009	0.78	0.0200	1.6%	0.78 [0.74; 0.82]
Bell 2015	0.74	0.0101	1.7%	0.74 [0.72; 0.76]
Berg 2013	0.82	0.0090	1.7%	0.82 [0.80; 0.84]
Bihorac 2019	0.80	0.0050	1.7%	0.80 [0.79; 0.81]
Birnie 2014	0.73	0.0050	1.7%	0.73 [0.72; 0.74]
Che 2019	0.82	0.0200	1.6%	0.82 [0.78; 0.86]
Chen 2020	0.87	0.0360	1.4%	0.87 [0.80; 0.94]
Coulson 2021	0.68	0.0040	1.7%	0.68 [0.67; 0.69]
Mehmet 2020	0.80	0.0305	1.5%	0.80 [0.74; 0.86]
Du 2021	0.97	0.0170	1.6%	0.97 [0.93; 1.00]
Grimm 2015	0.71	0.0108	1.7%	0.71 [0.69; 0.73]
Guan 2019	0.80	0.0010	1.7%	0.80 [0.79; 0.80]
Hofer 2020	0.79	0.0090	1.7%	0.79 [0.77; 0.81]
Hu 2021	0.80	0.0180	1.6%	0.80 [0.76; 0.83]
Hu 2020	0.78	0.0360	1.4%	0.78 [0.71; 0.85]
Jiang 2016	0.75	0.0065	1.7%	0.75 [0.74; 0.76]
Jorge-Monjas 2016	0.89	0.0200	1.6%	0.89 [0.85; 0.93]
Kalisvaart 2019	0.70	0.0155	1.6%	0.70 [0.67; 0.73]
Kashani 2015	0.67	0.0210	1.6%	0.67 [0.63; 0.71]
Kim 2014	0.90	0.0260	1.5%	0.90 [0.85; 0.95]
Kim 2011	0.73	0.0460	1.2%	0.73 [0.64; 0.82]
Ko 2020	0.78	0.0200	1.6%	0.78 [0.74; 0.82]
Lee 2018	0.78	0.0150	1.6%	0.78 [0.75; 0.81]
Lee 2018	0.90	0.0120	1.7%	0.90 [0.88; 0.92]
Legrand 2013	0.76	0.0350	1.4%	0.76 [0.69; 0.83]
Lei 2020	1.00	0.0032	1.7%	1.00 [0.99; 1.00]
Lei 2019	0.82	0.0080	1.7%	0.82 [0.80; 0.83]
Li 2020	0.76	0.0090	1.7%	0.76 [0.74; 0.77]
Lin 2020	0.74	0.0271	1.5%	0.74 [0.68; 0.79]
Liu 2016	0.89	0.0050	1.7%	0.89 [0.88; 0.90]
Martini 2019	0.74	0.0150	1.6%	0.74 [0.71; 0.77]
McBride 2019	0.88	0.0232	1.6%	0.88 [0.84; 0.93]
Metzger 2016	0.81	0.0397	1.3%	0.81 [0.73; 0.89]
Meyer 2018	0.96	0.0071	1.7%	0.96 [0.95; 0.97]
Nah 2016	0.80	0.0610	1.0%	0.80 [0.68; 0.92]
Ortega-Loubon 2018	0.78	0.0360	1.4%	0.78 [0.71; 0.85]
Pannu 2016	0.87	0.0100	1.7%	0.87 [0.85; 0.89]
Paparella 2014	0.84	0.0240	1.5%	0.84 [0.80; 0.89]
Penny-Dimri 2020	0.78	0.0035	1.7%	0.78 [0.77; 0.79]
Ramonell 2016	0.79	0.0121	1.7%	0.79 [0.77; 0.81]
Rank 2020	0.89	0.0158	1.6%	0.89 [0.86; 0.92]
Rueggeberg 2008	0.91	0.0459	1.2%	0.91 [0.82; 1.00]
Simonini 2014	0.84	0.0260	1.5%	0.84 [0.79; 0.89]
Slankamenac 2013	0.81	0.0260	1.5%	0.81 [0.76; 0.86]
Slankamenac 2009	0.80	0.0316	1.4%	0.80 [0.74; 0.86]
Thakar 2005	0.81	0.0150	1.6%	0.81 [0.78; 0.84]
Tian 2021	0.69	0.0050	1.7%	0.69 [0.68; 0.70]
Trongtrakul 2020	0.84	0.0070	1.7%	0.84 [0.83; 0.85]
Tseng 2020	0.84	0.0330	1.4%	0.84 [0.78; 0.91]
Xue 2021	0.85	0.0010	1.7%	0.85 [0.85; 0.85]
Yayac 2021	0.97	0.0040	1.7%	0.97 [0.96; 0.97]
Zhang 2020	0.80	0.0010	1.7%	0.80 [0.79; 0.80]
Zhou 2020	0.89	0.0345	1.4%	0.89 [0.82; 0.96]
Jeremiah 2013	0.72	0.0200	1.6%	0.72 [0.68; 0.76]
Rahendra 2006	0.75	0.1000	0.6%	0.75 [0.55; 0.95]
Duminda 2007	0.81	0.0150	1.6%	0.81 [0.78; 0.84]
H Paloma 2007	0.84	0.0320	1.4%	0.84 [0.78; 0.91]
Sevag 2012	0.88	0.0080	1.7%	0.88 [0.86; 0.89]
Sachin 2009	0.80	0.0094	1.7%	0.80 [0.78; 0.82]
Ng 2014	0.81	0.0055	1.7%	0.81 [0.80; 0.82]
Kim 2013	0.84	0.0770	0.8%	0.84 [0.69; 0.99]
**Total (95% CI)**			**100.0%**	**0.81 [0.79; 0.83]**
**Prediction interval**				**[0.66; 0.96]**

Heterogeneity: $Tau^2 = 0.0055$; $Chi^2 = 10831.27$, df = 63 (P = 0); $I^2 = 99\%$

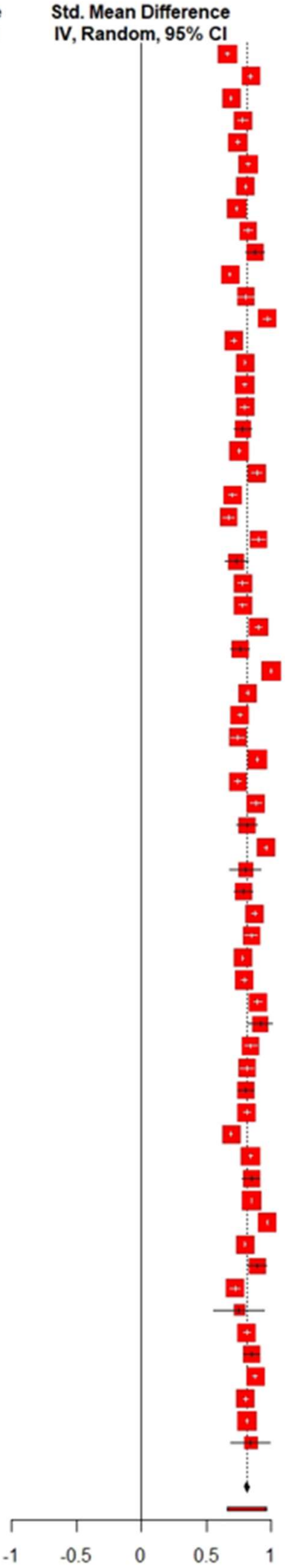

Note: The red line indicates 95% prediction interval, which is wider than 95% CI.

© 2023 Feng Y et al. *JAMA Network Open.*

eFigure 3. Forest Plot for Meta-analysis of C Statistics of Prediction Models of AKI in ICU

Study	TE	SE	Weight	Std. Mean Difference IV, Random, 95% CI
An 2020	0.88	0.0222	4.8%	0.88 [0.84; 0.92]
Asada 2016	0.94	0.0750	2.8%	0.94 [0.79; 1.09]
Bhatraju 2019	0.95	0.0200	4.8%	0.95 [0.91; 0.99]
Chen 2019	0.81	0.0150	5.0%	0.81 [0.78; 0.84]
Chiofolo 2019	0.91	0.0050	5.1%	0.91 [0.90; 0.92]
Deng 2020	0.80	0.0100	5.1%	0.80 [0.78; 0.82]
Deng 2017	0.76	0.0170	4.9%	0.76 [0.72; 0.79]
Deng 2020	0.81	0.0270	4.6%	0.81 [0.76; 0.86]
Ferrari 2019	0.73	0.0260	4.7%	0.73 [0.68; 0.78]
Ferrari 2019	0.70	0.0260	4.7%	0.70 [0.65; 0.75]
Flechet 2017	0.86	0.0001	5.1%	0.86 [0.86; 0.86]
Malhotra 2017	0.79	0.0520	3.7%	0.79 [0.69; 0.89]
Matsuura 2020	0.79	0.0100	5.1%	0.79 [0.77; 0.81]
Mohamadlou 2018	0.67	0.0030	5.1%	0.67 [0.67; 0.68]
Nusshag 2019	0.93	0.0360	4.3%	0.93 [0.86; 1.00]
Parreco 2019	0.83	0.0023	5.1%	0.83 [0.83; 0.84]
Wang 2019	0.83	0.0170	4.9%	0.83 [0.80; 0.87]
Wang 2020	0.78	0.0056	5.1%	0.78 [0.77; 0.79]
Wiersema 2020	0.70	0.0200	4.8%	0.70 [0.66; 0.74]
Xie 2021	0.99	0.0095	5.1%	0.99 [0.97; 1.00]
Zimmerman 2019	0.78	0.0040	5.1%	0.78 [0.78; 0.79]
**Total (95% CI)**			**100.0%**	**0.82 [0.78; 0.86]**
**Prediction interval**				**[0.64; 1.00]**

Heterogeneity: $Tau^2 = 0.0070$; $Chi^2 = 5127.58$, df = 20 (P = 0); $I^2 = 100\%$

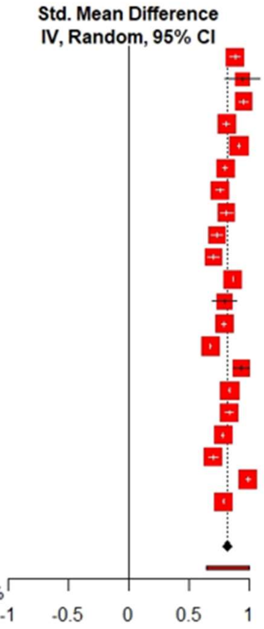

Note: The red line indicates 95% prediction interval, which is wider than 95% CI.

© 2023 Feng Y et al. *JAMA Network Open.*

eFigure 4. Forest Plot for Meta-analysis of C Statistics of Prediction Models of AKI in General Hospitalization

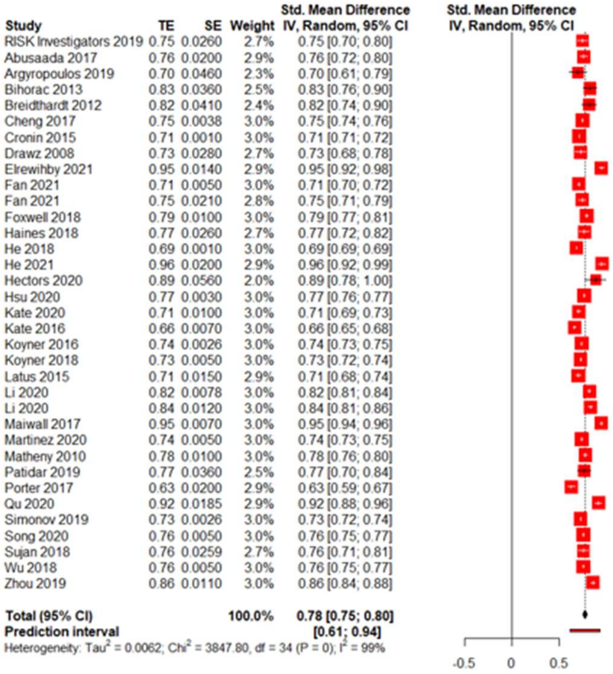

Note: The red line indicates 95% prediction interval, which is wider than 95% CI.

© 2023 Feng Y et al. *JAMA Network Open.*

eFigure 5. Drapery Plot for Meta-analysis of C Statistics of Contrast Medium Associated AKI Prediction Models

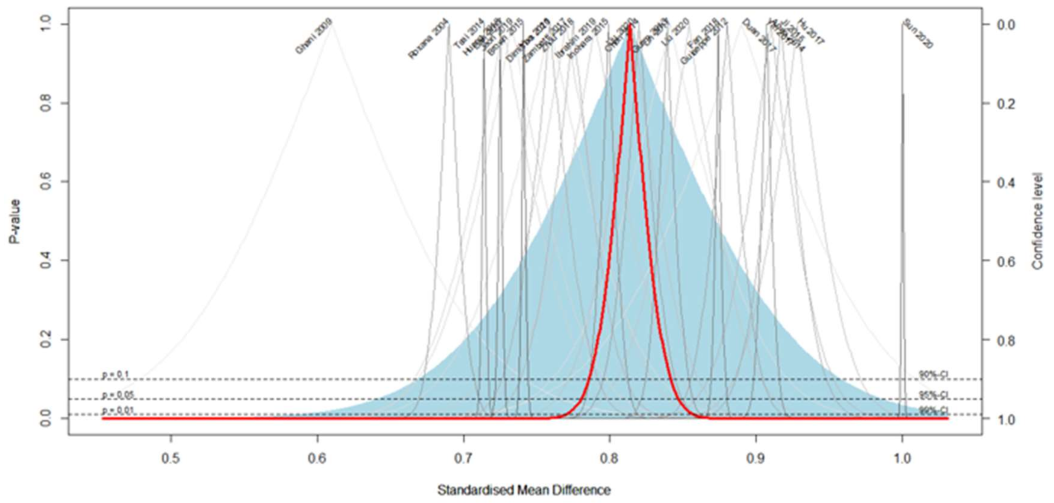

Note: The red line indicates 95% confidence interval, and the blue shadowed area indicates 95% prediction interval.

© 2023 Feng Y et al. *JAMA Network Open.*

eFigure 6. Drapery Plot for Meta-analysis of C Statistics of Postoperative AKI Prediction Models

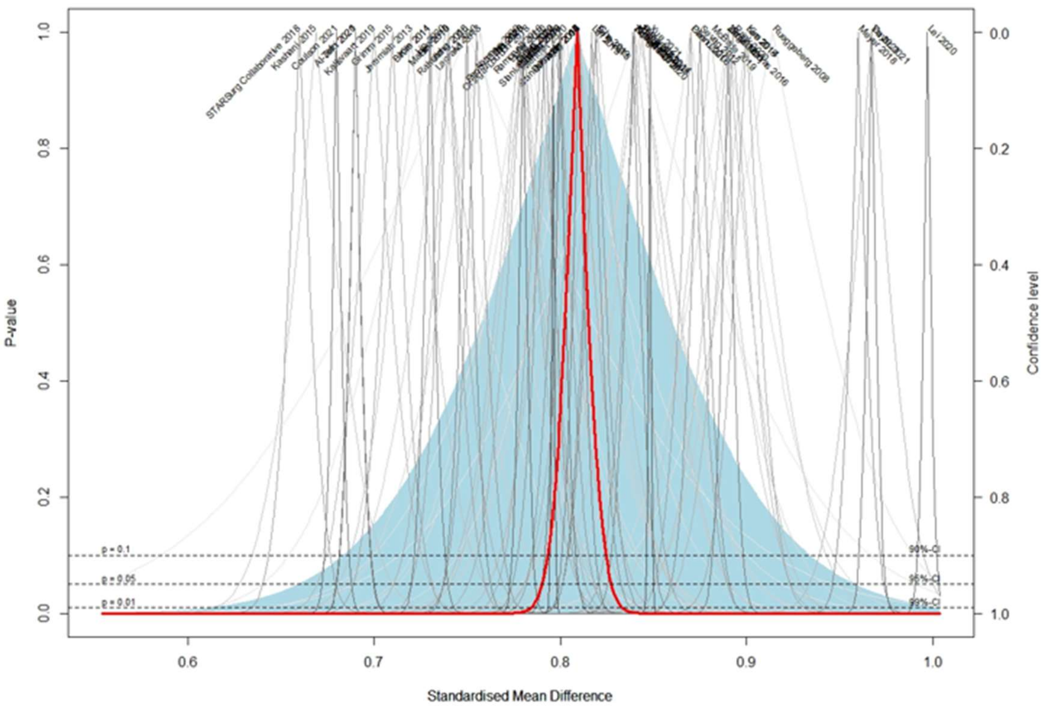

Note: The red line indicates 95% confidence interval, and the blue shadowed area indicates 95% prediction interval.

© 2023 Feng Y et al. *JAMA Network Open.*

eFigure 7. Drapery Plot for Meta-analysis of C Statistics of Prediction Models of AKI in ICU

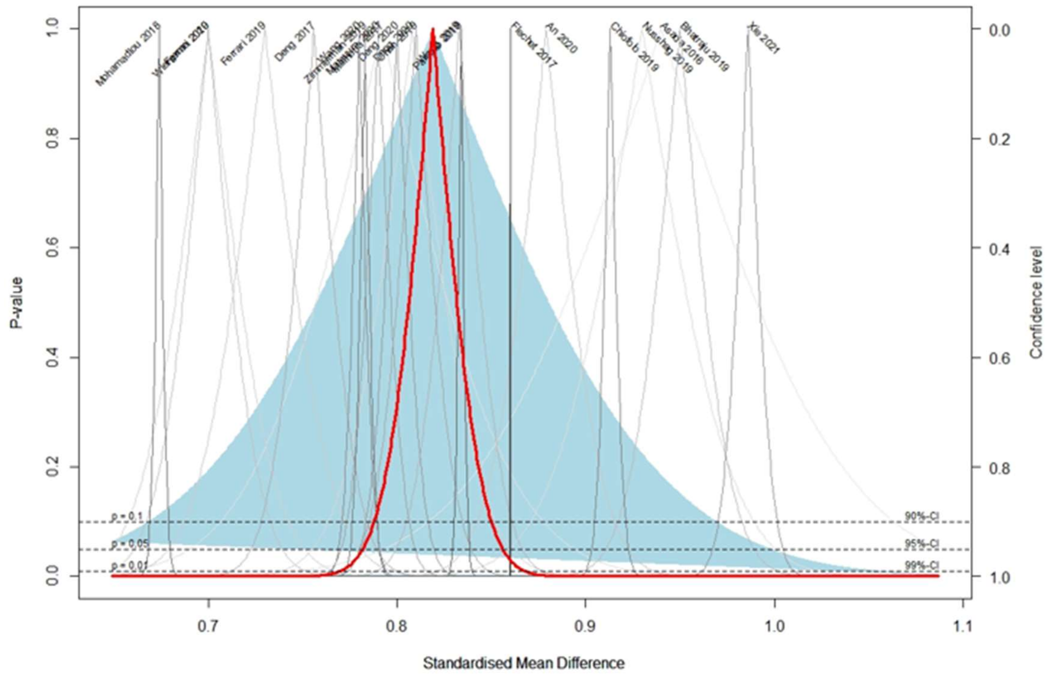

Note: The red line indicates 95% confidence interval, and the blue shadowed area indicates 95% prediction interval.

© 2023 Feng Y et al. *JAMA Network Open.*

eFigure 8. Drapery Plot for Meta-analysis of C Statistics of Prediction Models of AKI in General Hospitalization

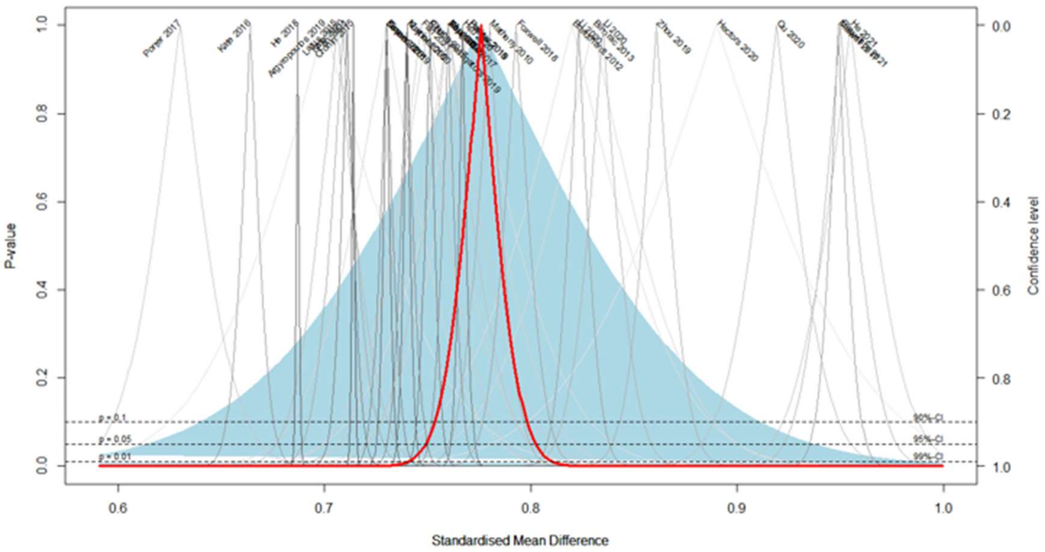

Note: The red line indicates 95% confidence interval, and the blue shadowed area indicates 95% prediction interval.

© 2023 Feng Y et al. *JAMA Network Open.*

eFigure 9. Summary ROC Curves of C Statistics of Contrast Medium Associated AKI Prediction Models

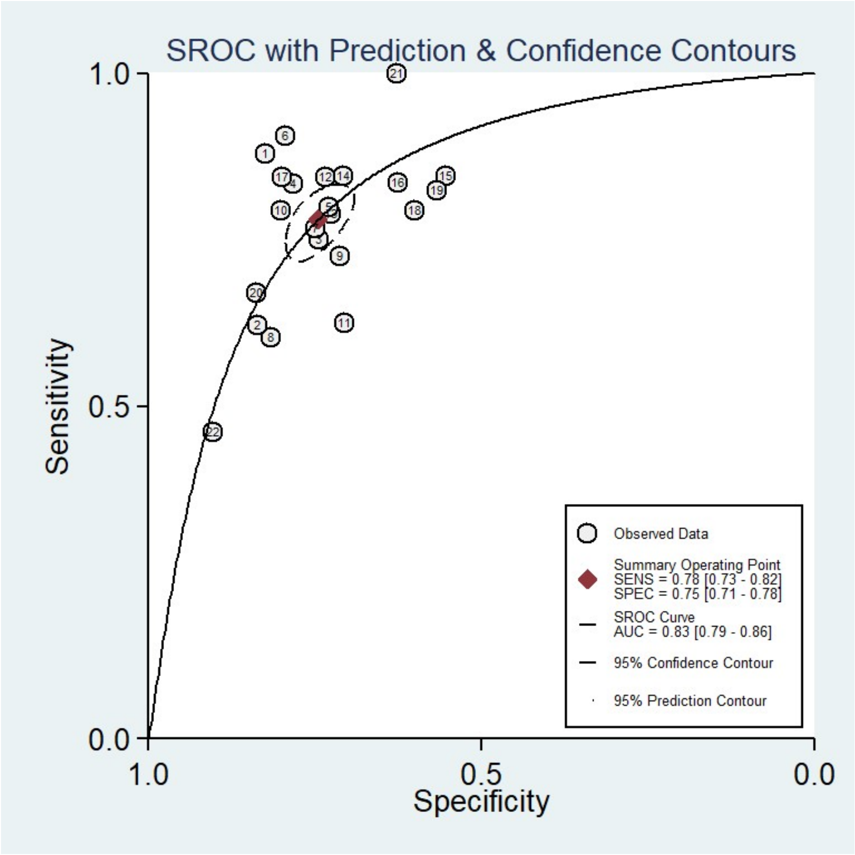

© 2023 Feng Y et al. *JAMA Network Open.*

eFigure 10. Summary ROC curves of C Statistics of Postoperative AKI Prediction Models

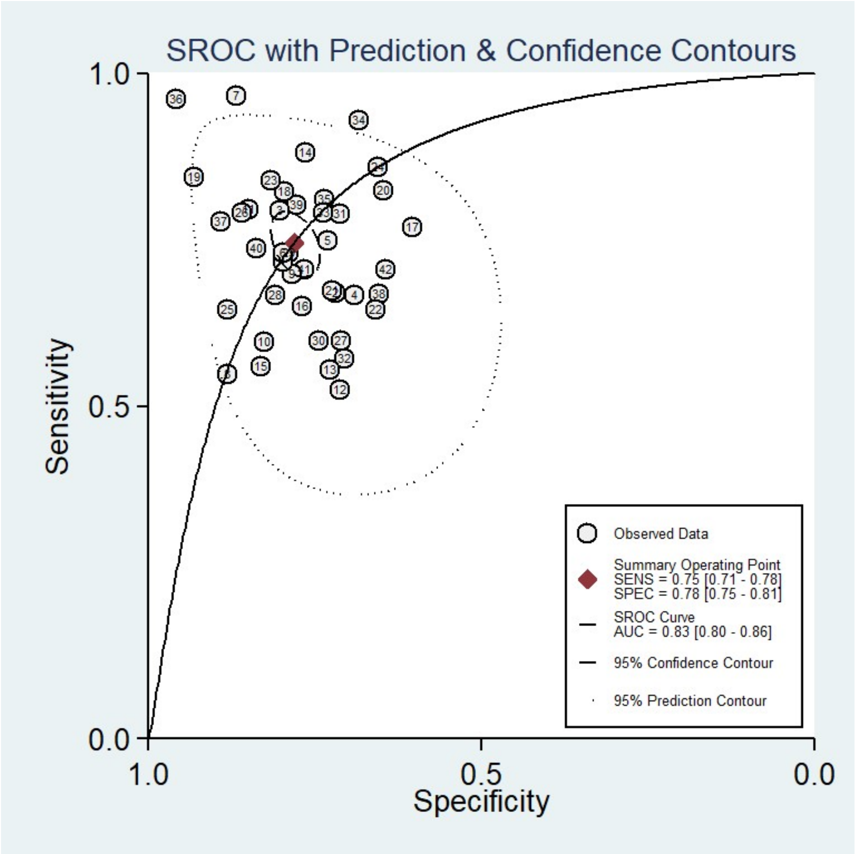

© 2023 Feng Y et al. *JAMA Network Open.*

eFigure 11. Summary ROC Curves of C Statistics of Prediction Models of AKI in ICU

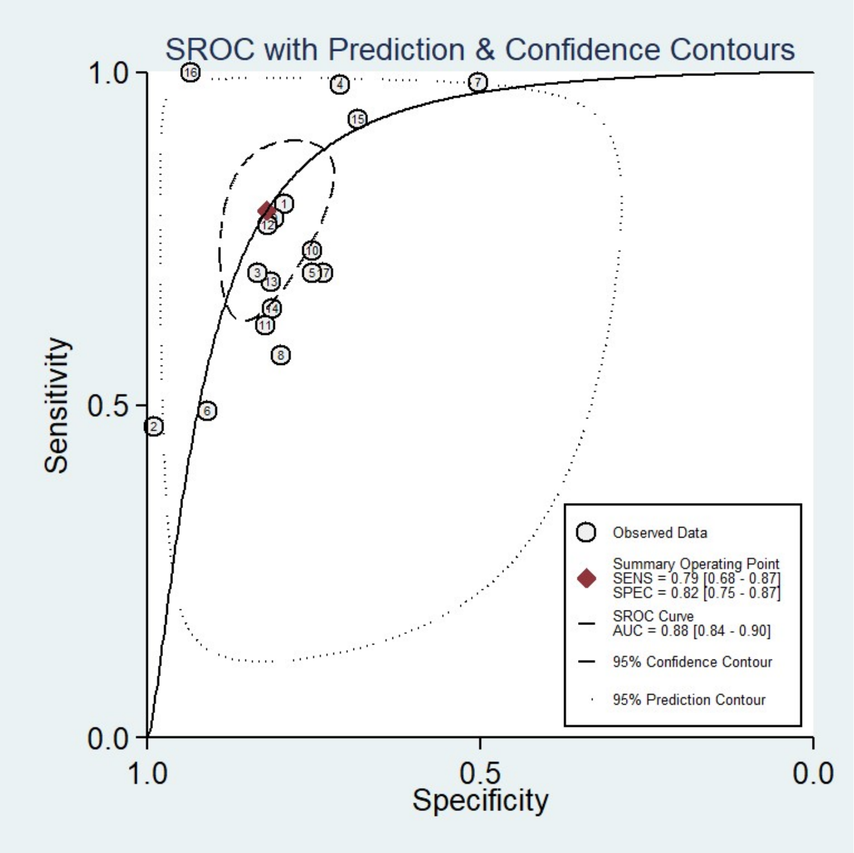

© 2023 Feng Y et al. *JAMA Network Open.*

eFigure 12. Summary ROC Curves of C Statistics of Prediction Models of AKI in General Hospitalization

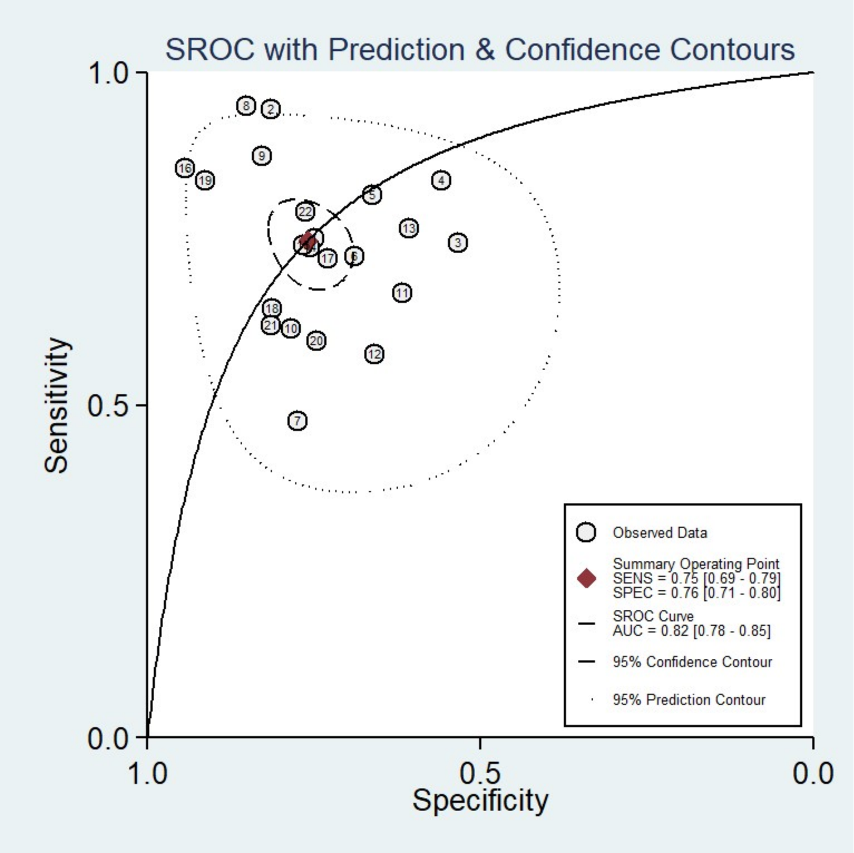

© 2023 Feng Y et al. *JAMA Network Open.*

eFigure 13. Bayesian Analysis of C Statistics of AKI Prediction Models in All Clinical Settings

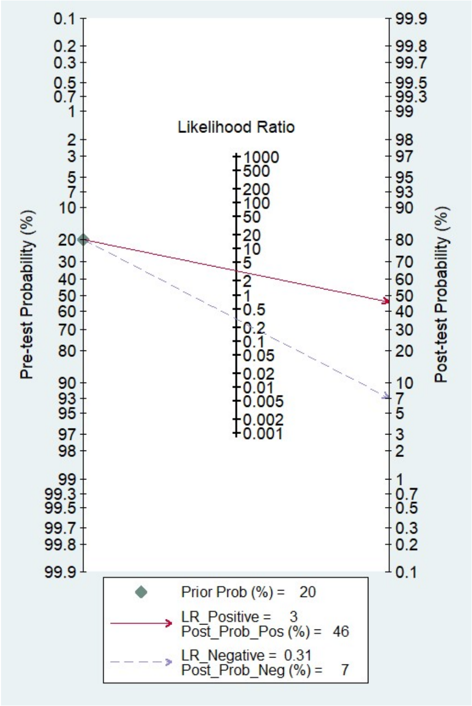

© 2023 Feng Y et al. *JAMA Network Open.*

eFigure 14. Bayesian Analysis of C Statistics of Contrast Medium Associated AKI Prediction Models

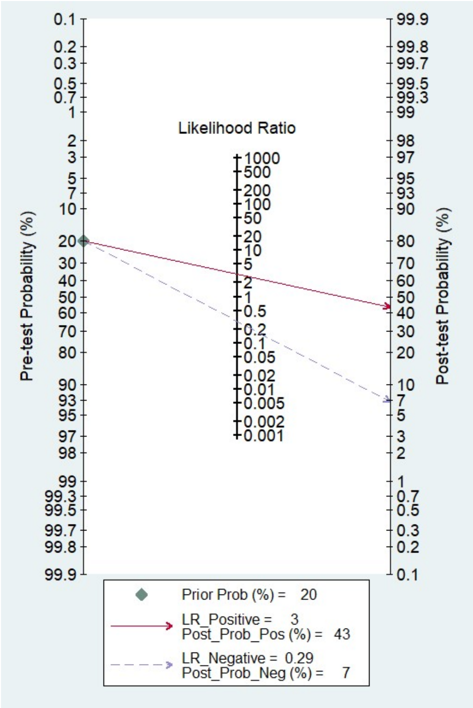

© 2023 Feng Y et al. *JAMA Network Open.*

eFigure 15. Bayesian Analysis of C Statistics of Postoperative AKI Prediction Models

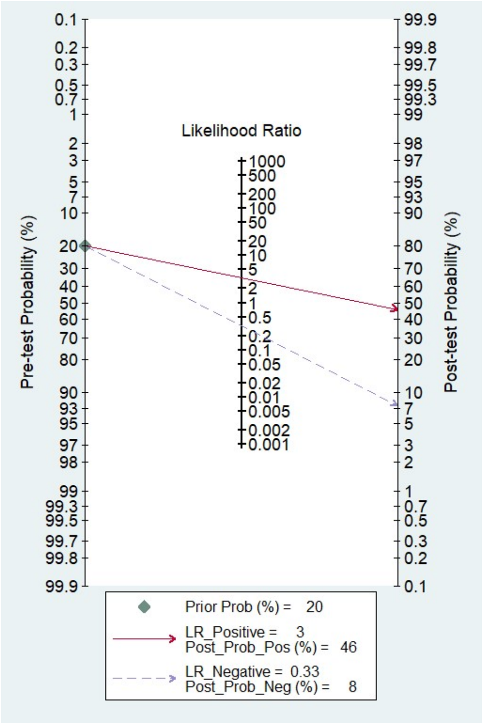

© 2023 Feng Y et al. *JAMA Network Open.*

eFigure 16. Bayesian Analysis of C Statistics of Prediction Models of AKI in ICU

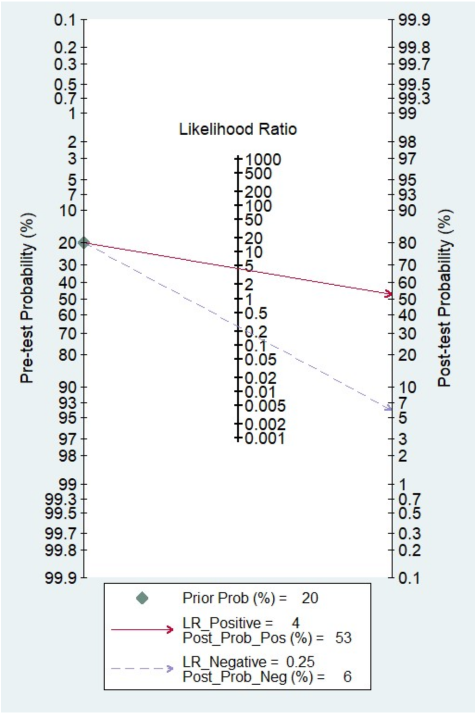

© 2023 Feng Y et al. *JAMA Network Open.*

eFigure 17. Bayesian Analysis of C Statistics of Prediction Models of AKI in General Hospitalization

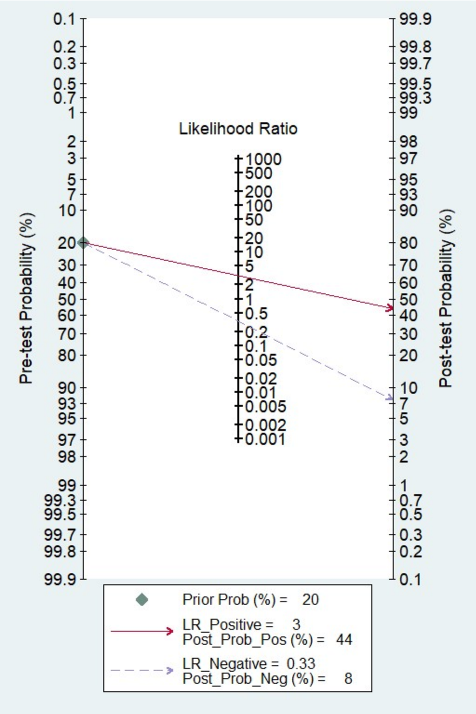

© 2023 Feng Y et al. *JAMA Network Open.*

eFigure 18. Metaregression Analysis of Variables That Were Suspected to Be the Sources of Heterogeneities Against C Statistics (Weighted Against Standard Errors of C Statistics)

(A) C-statistics against AKI events

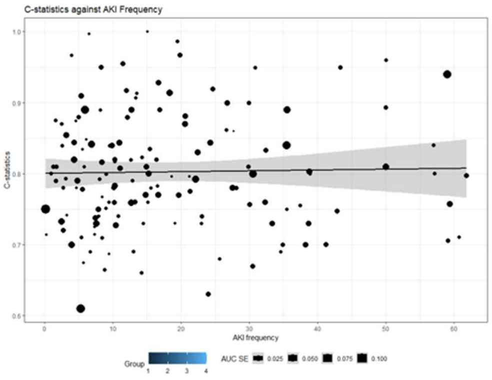

(B) C-statistics against predictor numbers

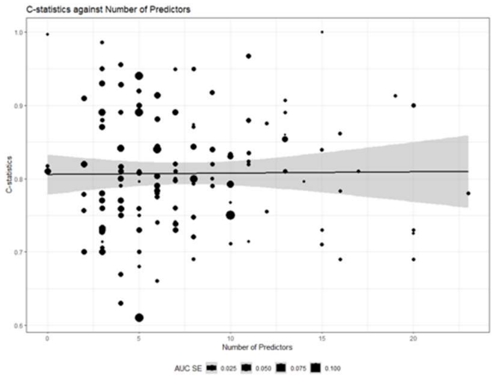

(C) C-statistics against publication year

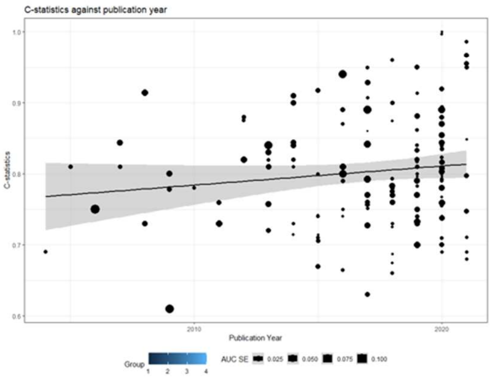

(D) C-statistics against AKI stage

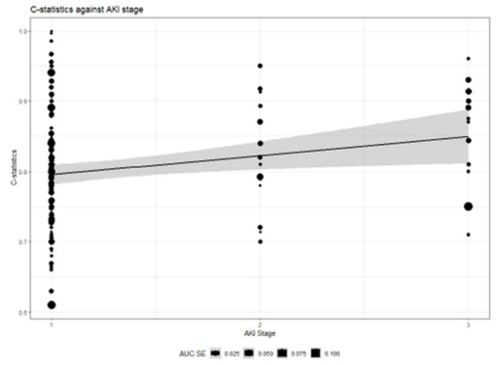

Note: Meta regression analysis indicated that the number of predictive variables in the model, AKI event rate rates, publication year, or predicted AKI stage were not source of the heterogeneity in the pooled C statistics.

© 2023 Feng Y et al. *JAMA Network Open.*

eFigure 19. The Baujat Plot for Between-Study Heterogeneity Analysis of Contrast Medium Associated AKI Prediction Models

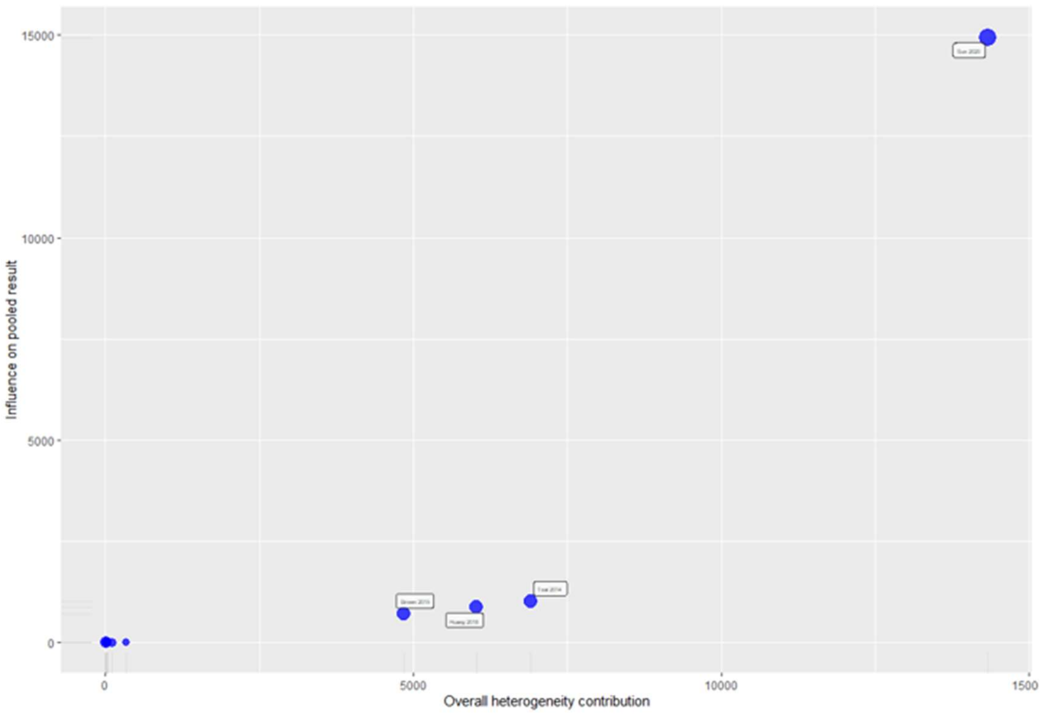

Note: this plot shows the contribution of each study to the overall heterogeneity of a meta-analysis, as measured by Cochran's Q test, on the horizontal axis, and the influence of the corresponding study on the pooled effect size on the vertical axis, thus detecting studies that contribute to the between-study heterogeneity.

© 2023 Feng Y et al. *JAMA Network Open.*

eFigure 20. The Baujat Plot for Between-Study Heterogeneity Analysis of Postoperative AKI Prediction Models

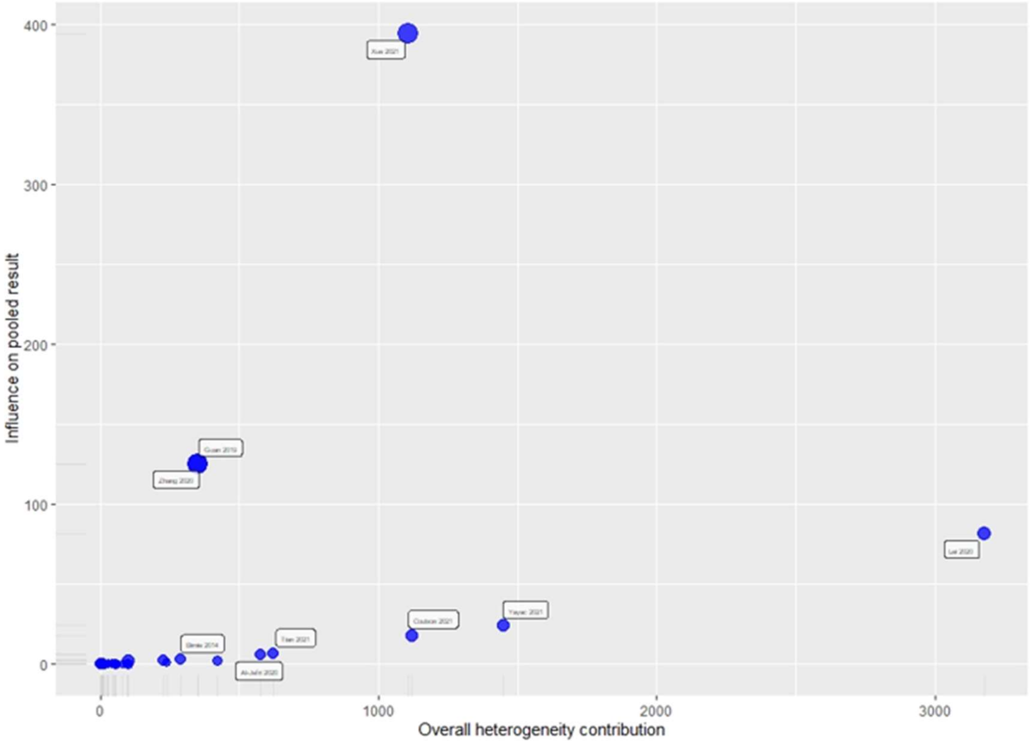

Note: this plot shows the contribution of each study to the overall heterogeneity of a meta-analysis, as measured by Cochran's Q test, on the horizontal axis, and the influence of the corresponding study on the pooled effect size on the vertical axis, thus detecting studies that contribute to the between-study heterogeneity.

© 2023 Feng Y et al. *JAMA Network Open.*

eFigure 21. The Baujat Plot for Between-Study Heterogeneity Analysis of Prediction Models of AKI in ICU

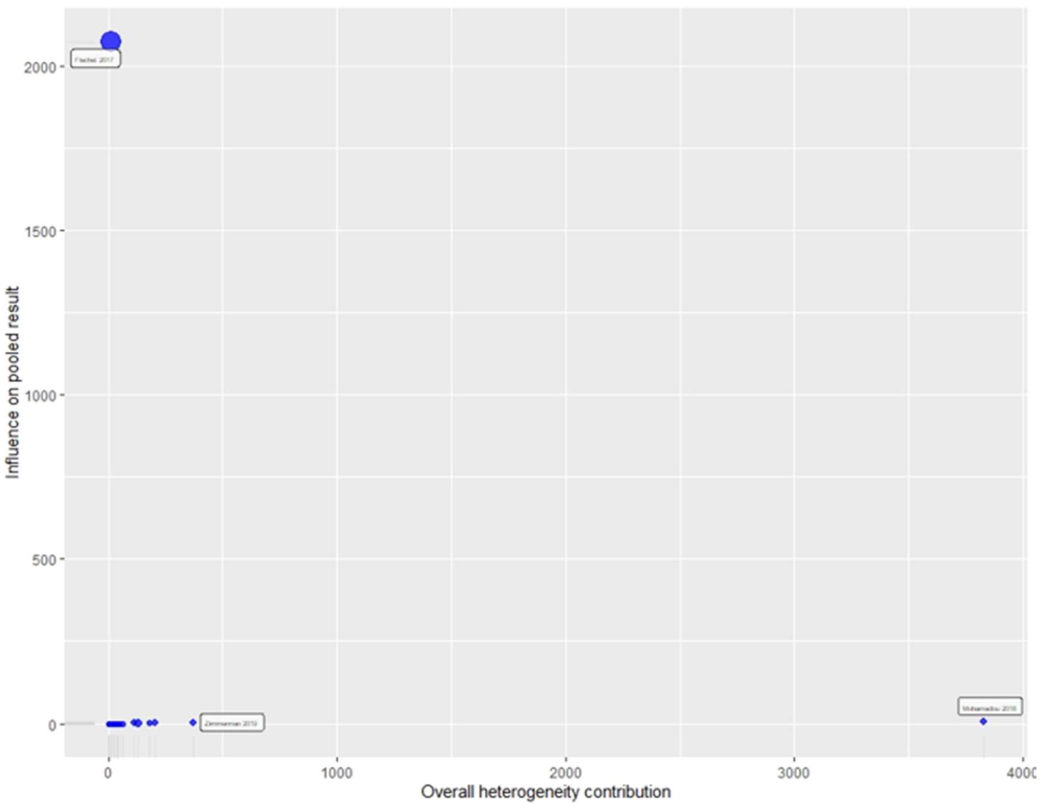

Note: this plot shows the contribution of each study to the overall heterogeneity of a meta-analysis, as measured by Cochran's Q test, on the horizontal axis, and the influence of the corresponding study on the pooled effect size on the vertical axis, thus detecting studies that contribute to the between-study heterogeneity.

© 2023 Feng Y et al. *JAMA Network Open.*

eFigure 22. The Baujat Plot for Between-Study Heterogeneity Analysis of Prediction Models of AKI in General Hospitalization

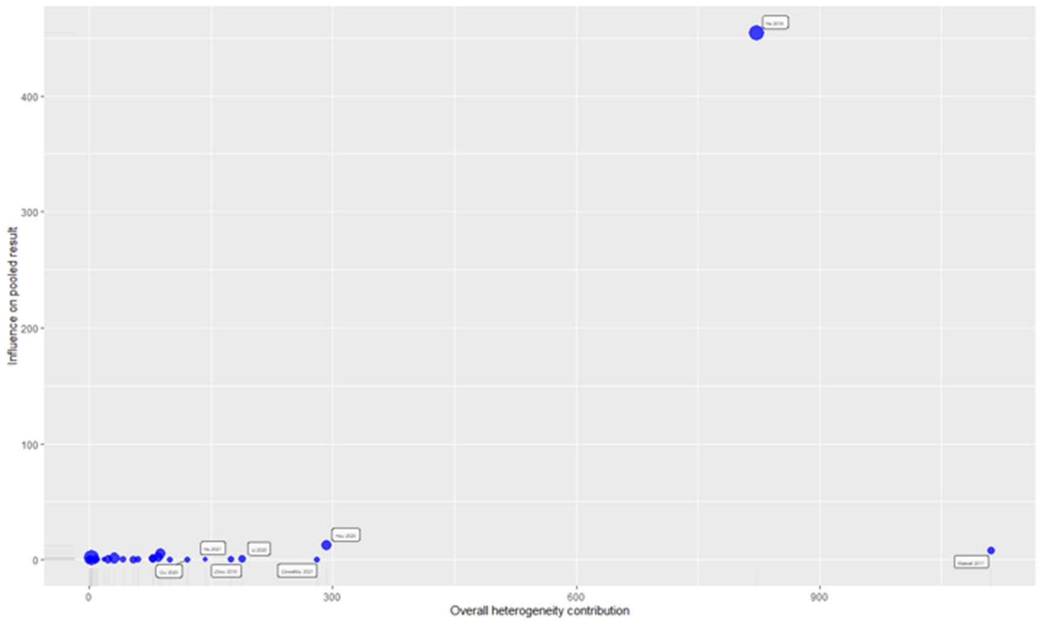

Note: this plot shows the contribution of each study to the overall heterogeneity of a meta-analysis, as measured by Cochran's Q test, on the horizontal axis, and the influence of the corresponding study on the pooled effect size on the vertical axis, thus detecting studies that contribute to the between-study heterogeneity.

© 2023 Feng Y et al. *JAMA Network Open.*

eFigure 23. Influence Analysis for Between-Study Heterogeneity Analysis of Contrast Medium Associated AKI Prediction Models

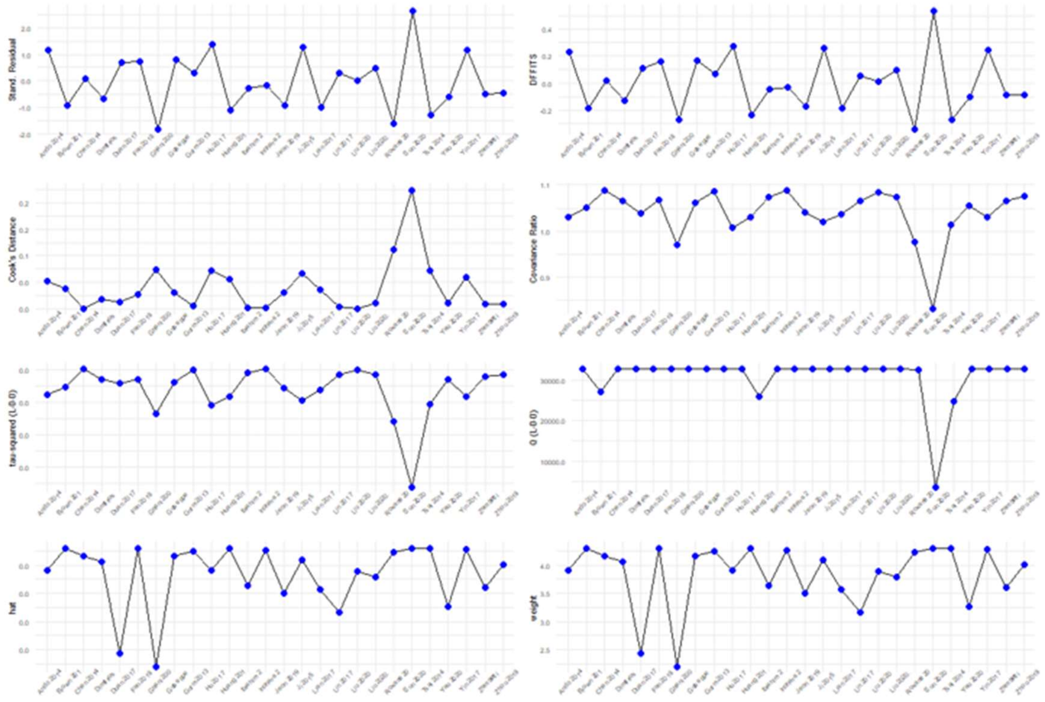

© 2023 Feng Y et al. *JAMA Network Open.*

eFigure 24. Influence Analysis for Between-Study Heterogeneity Analysis of Postoperative AKI Prediction Models

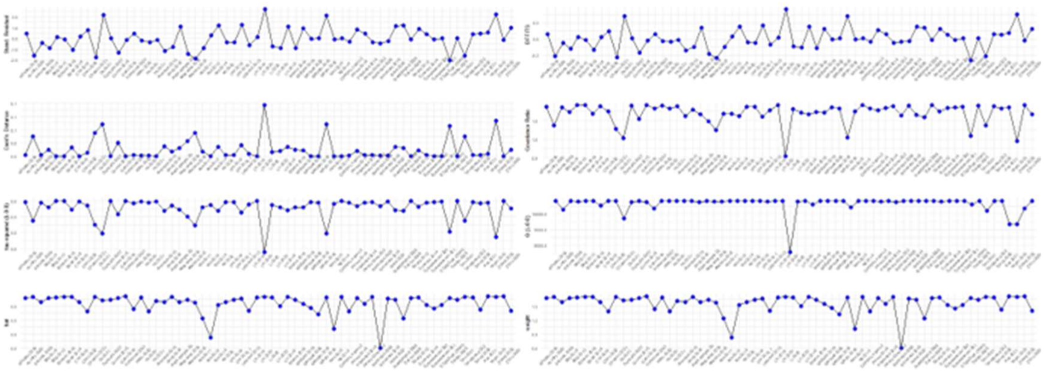

© 2023 Feng Y et al. *JAMA Network Open.*

eFigure 25. Influence Analysis for Between-Study Heterogeneity Analysis of Prediction Models of AKI in ICU

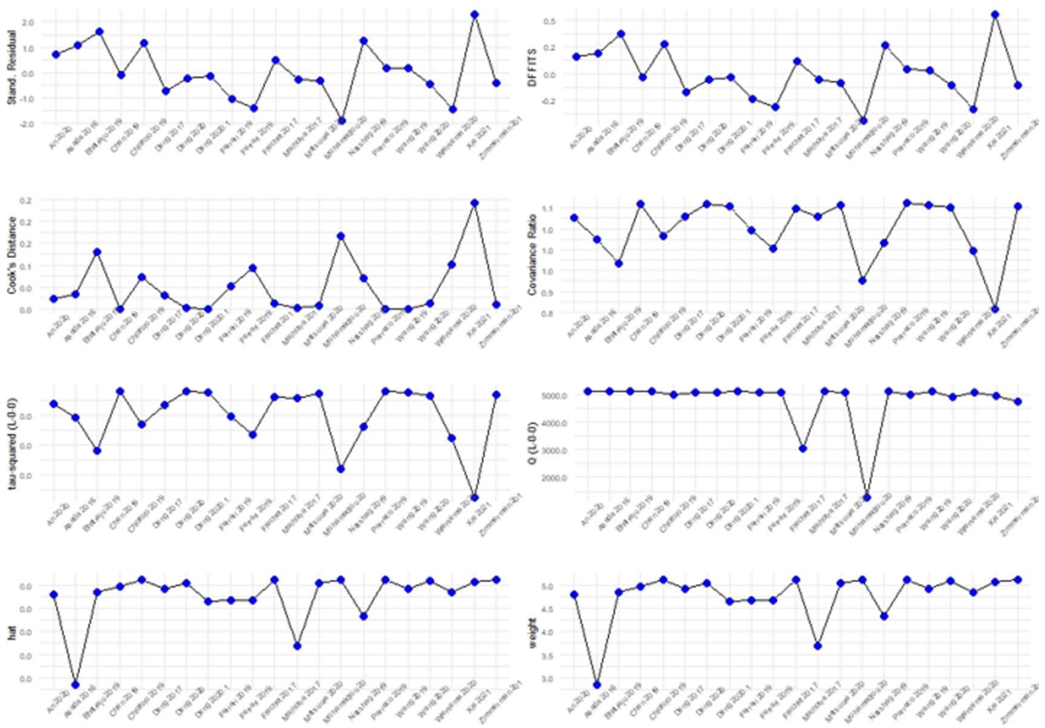

© 2023 Feng Y et al. *JAMA Network Open.*

eFigure 26. Influence Analysis for Between-Study Heterogeneity Analysis of Prediction Models of AKI in General Hospitalization

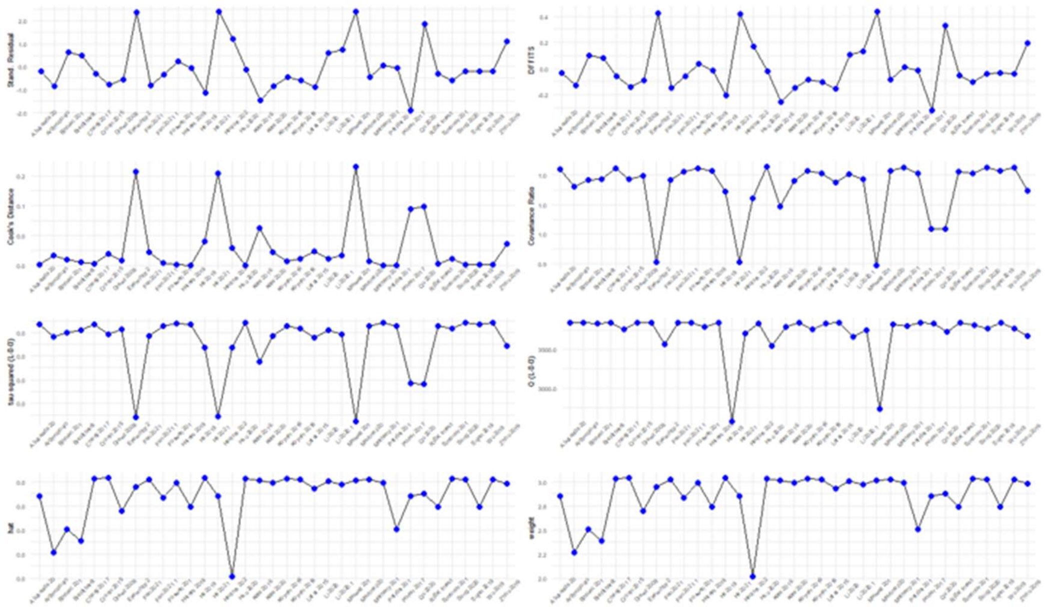

© 2023 Feng Y et al. *JAMA Network Open.*

eFigure 27. The Comparisons Between Reported Standard Error and Estimated Error of C Statistics of Each Study in the Whole Population and Each Subgroup

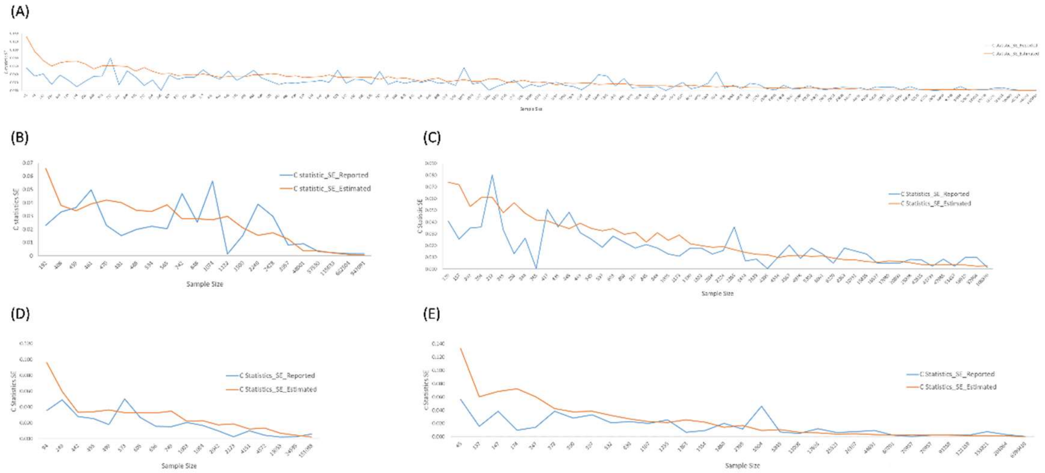

Note: The comparison of reported standard error (SE) (blue line) and estimated SE (orange line) of each study in the whole population (A), contrast medium associated AKI (B), post-operative AKI (C), AKI in ICU (D), and AKI in general hospitalizations (E). Generally, the reported SE in studies with small sample size tended to be smaller than estimated SE. The differences between reported SE and estimated SE decreased with the increase of sample size.

© 2023 Feng Y et al. *JAMA Network Open.*

eFigure 28. Funnel Plot Analysis for Meta-analysis of C Statistics of Contrast Medium Associated AKI Prediction Models

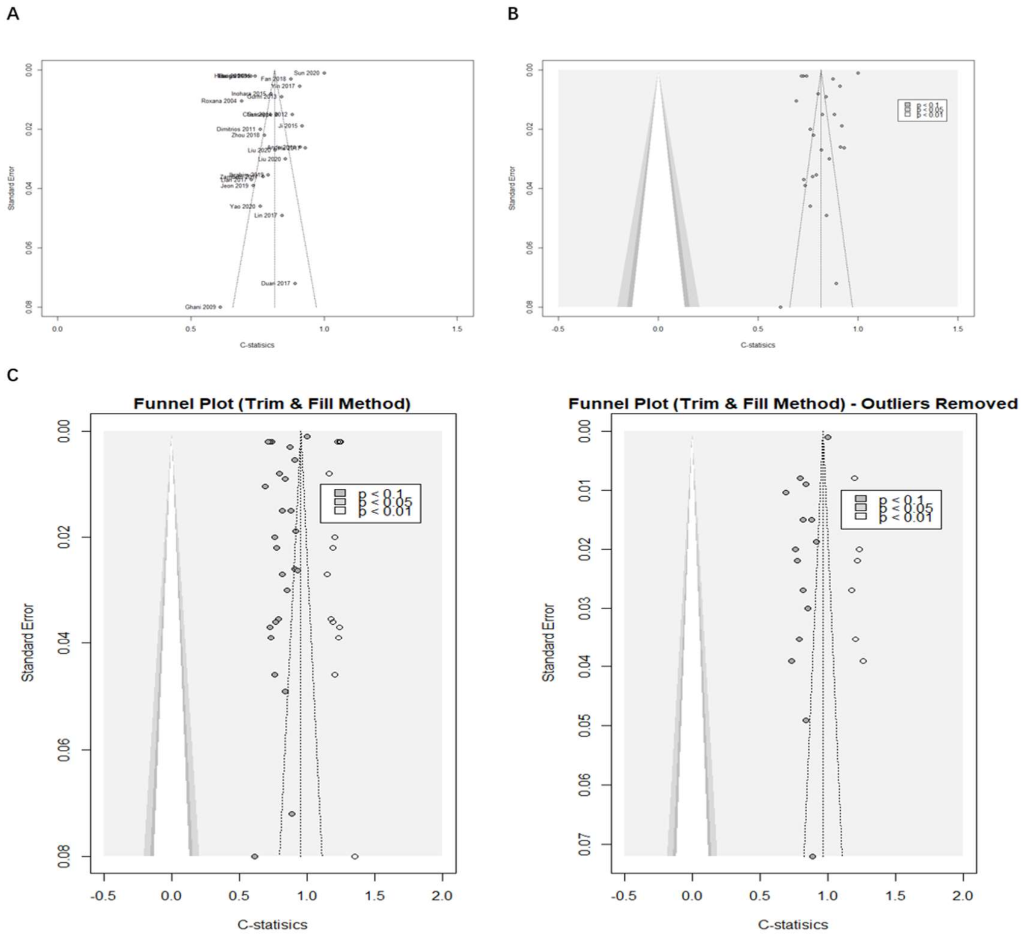

© 2023 Feng Y et al. *JAMA Network Open.*

eFigure 29. Funnel Plot Analysis for Meta-analysis of C Statistics of Postoperative AKI Prediction Models

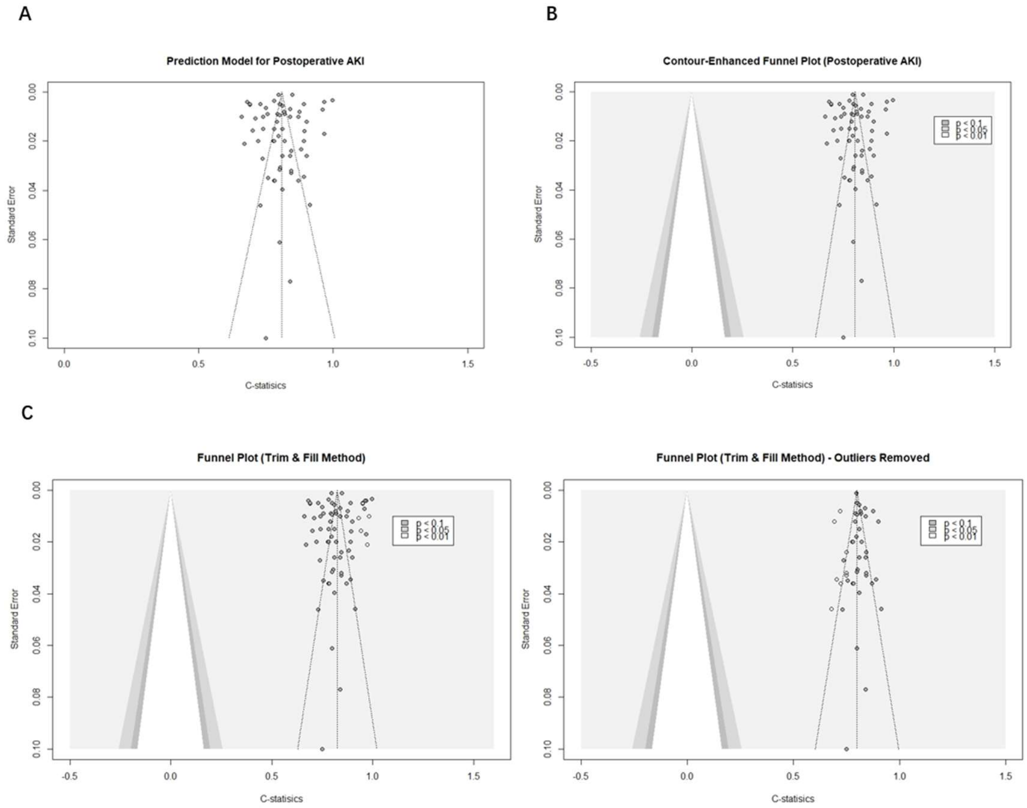

© 2023 Feng Y et al. *JAMA Network Open.*

eFigure 30. Funnel Plot Analysis for Meta-analysis of C Statistics of Prediction Models of AKI in ICU

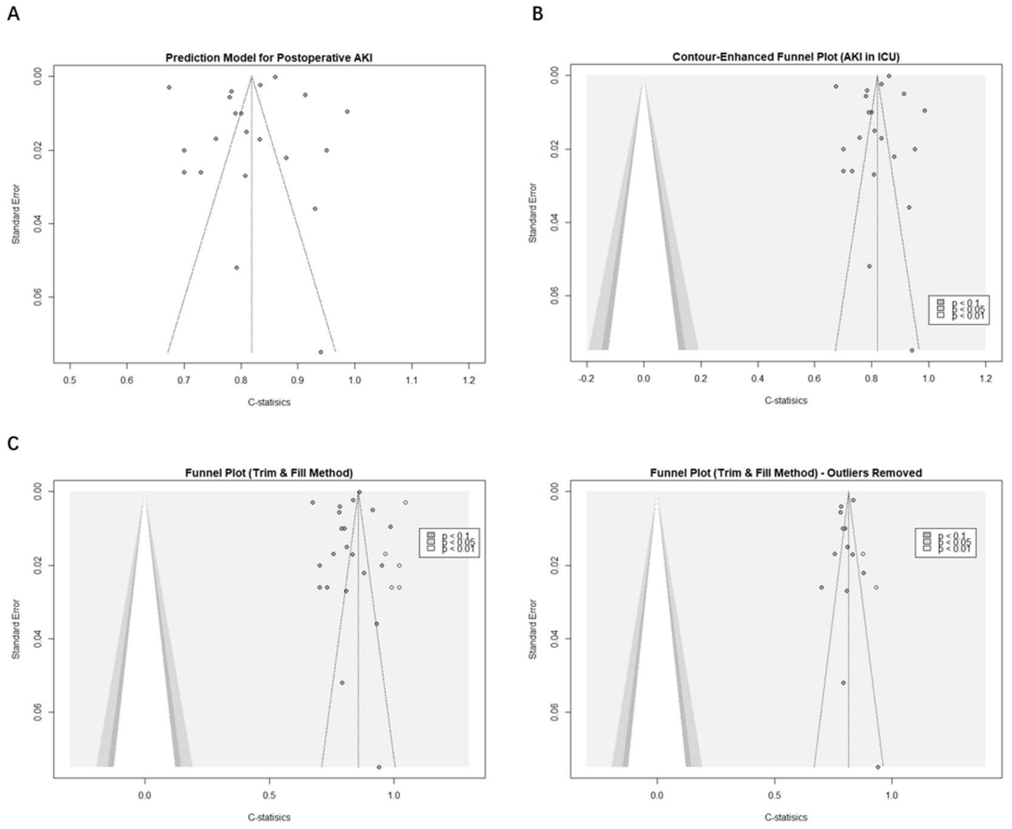

© 2023 Feng Y et al. *JAMA Network Open.*

eFigure 31. Funnel Plot Analysis for Meta-analysis of C Statistics of Prediction Models of AKI in General Hospitalization

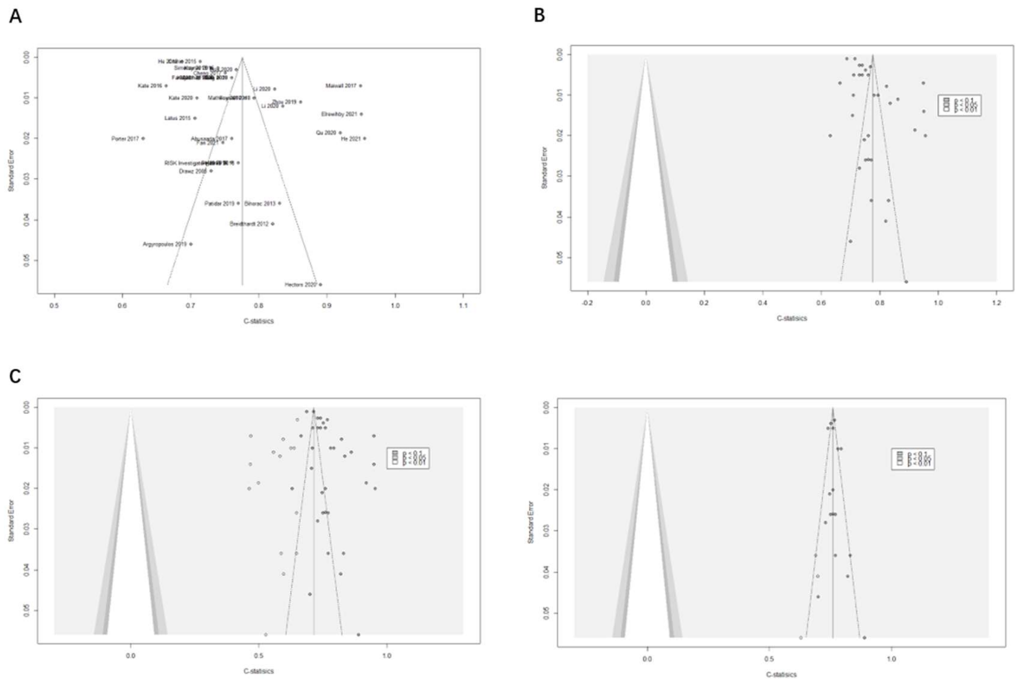

© 2023 Feng Y et al. *JAMA Network Open.*

eFigure 32. Risk of Bias Assessment Results Using PROBAST Tool for Prediction Models on Contrast Medium Associated AKI

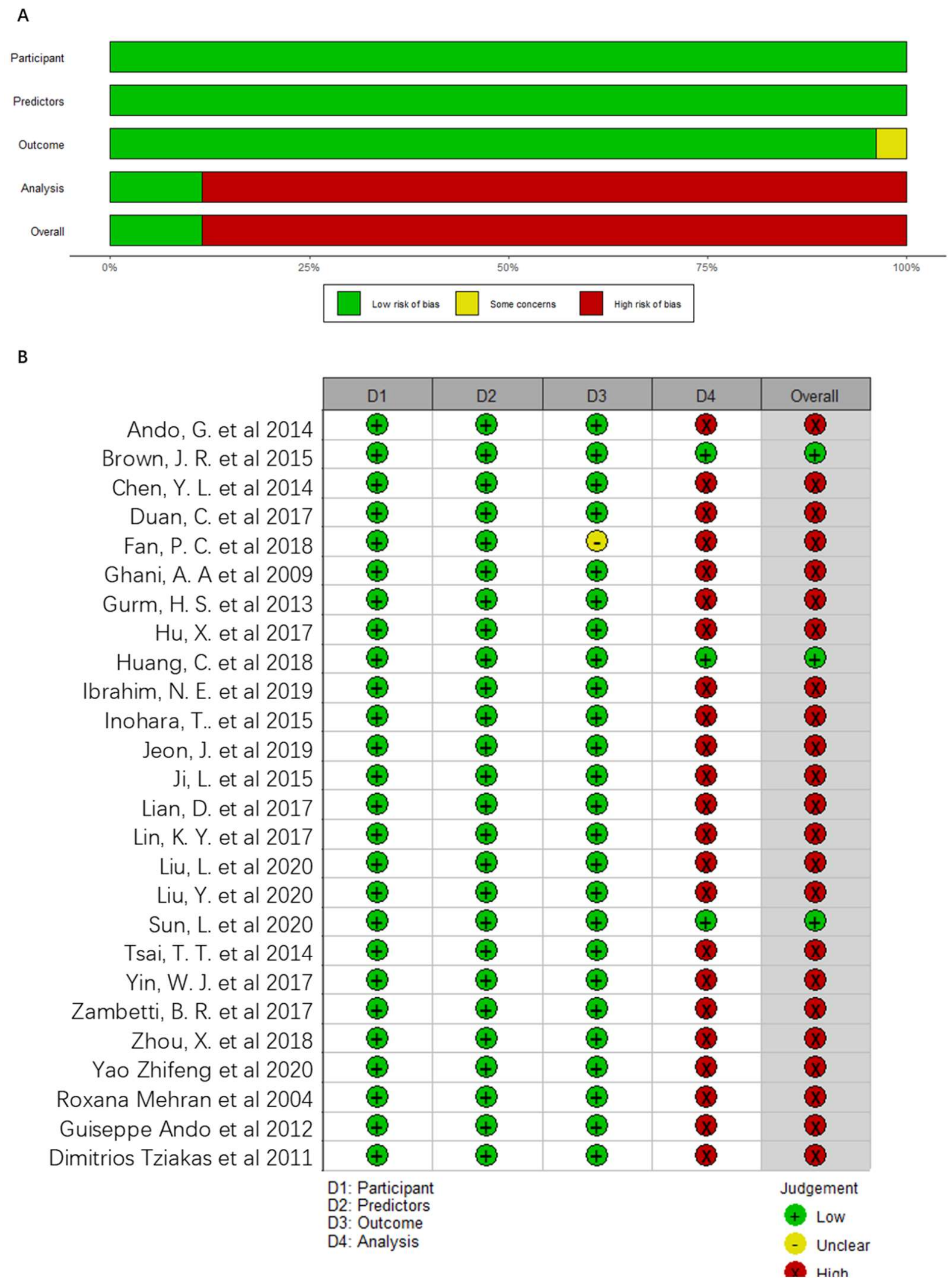

Note: (A) Summary of risk of bias assessment. (B) Traffic light plot of risk of bias assessment.

© 2023 Feng Y et al. *JAMA Network Open.*

eFigure 33. Risk of Bias Assessment Results Using PROBAST Tool for Prediction Models on Postoperative AKI

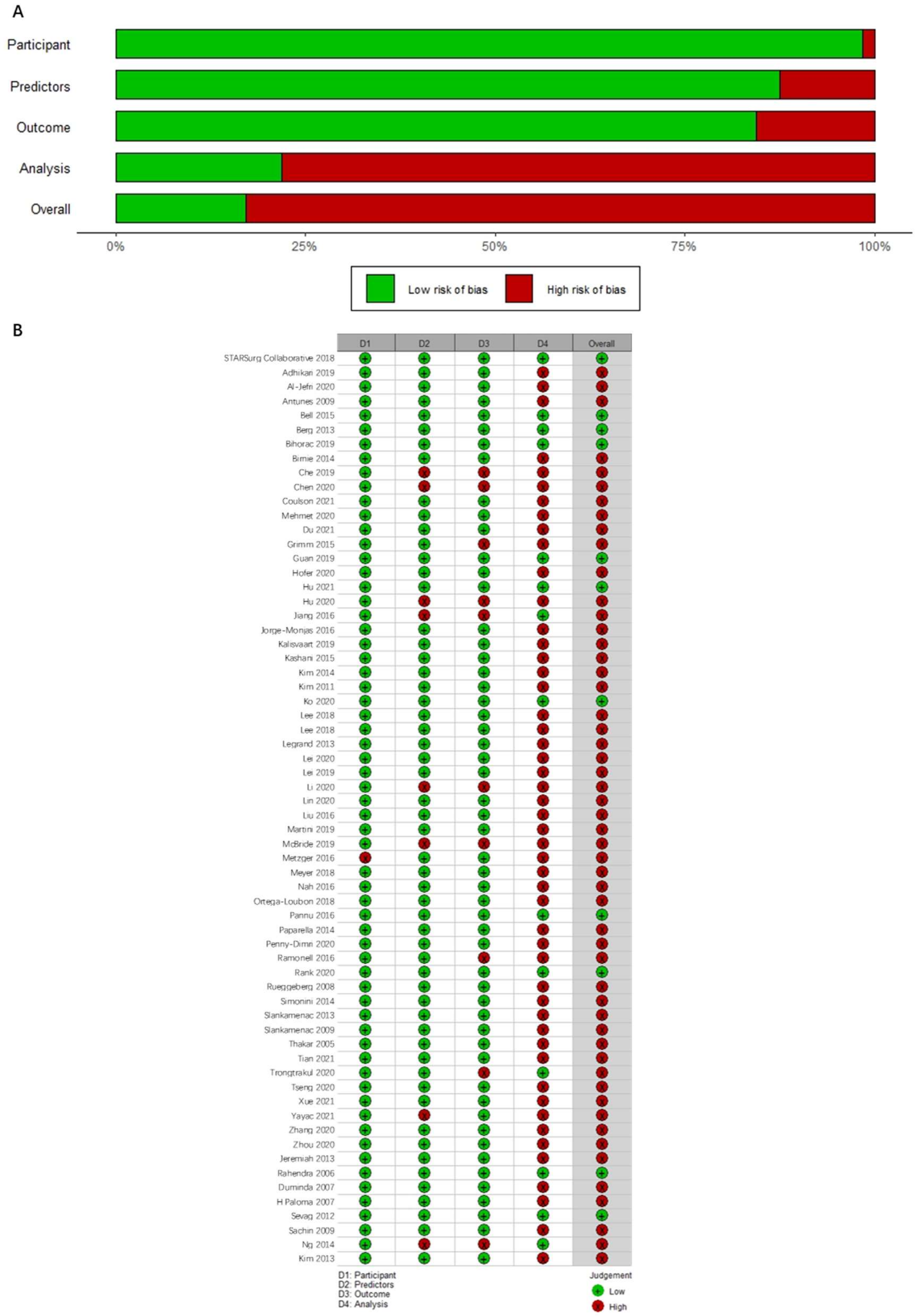

Note: (A) Summary of risk of bias assessment. (B) Traffic light plot of risk of bias assessment.

© 2023 Feng Y et al. *JAMA Network Open.*

eFigure 34. Risk of Bias Assessment Results Using PROBAST Tool for Prediction Models on Postoperative AKI

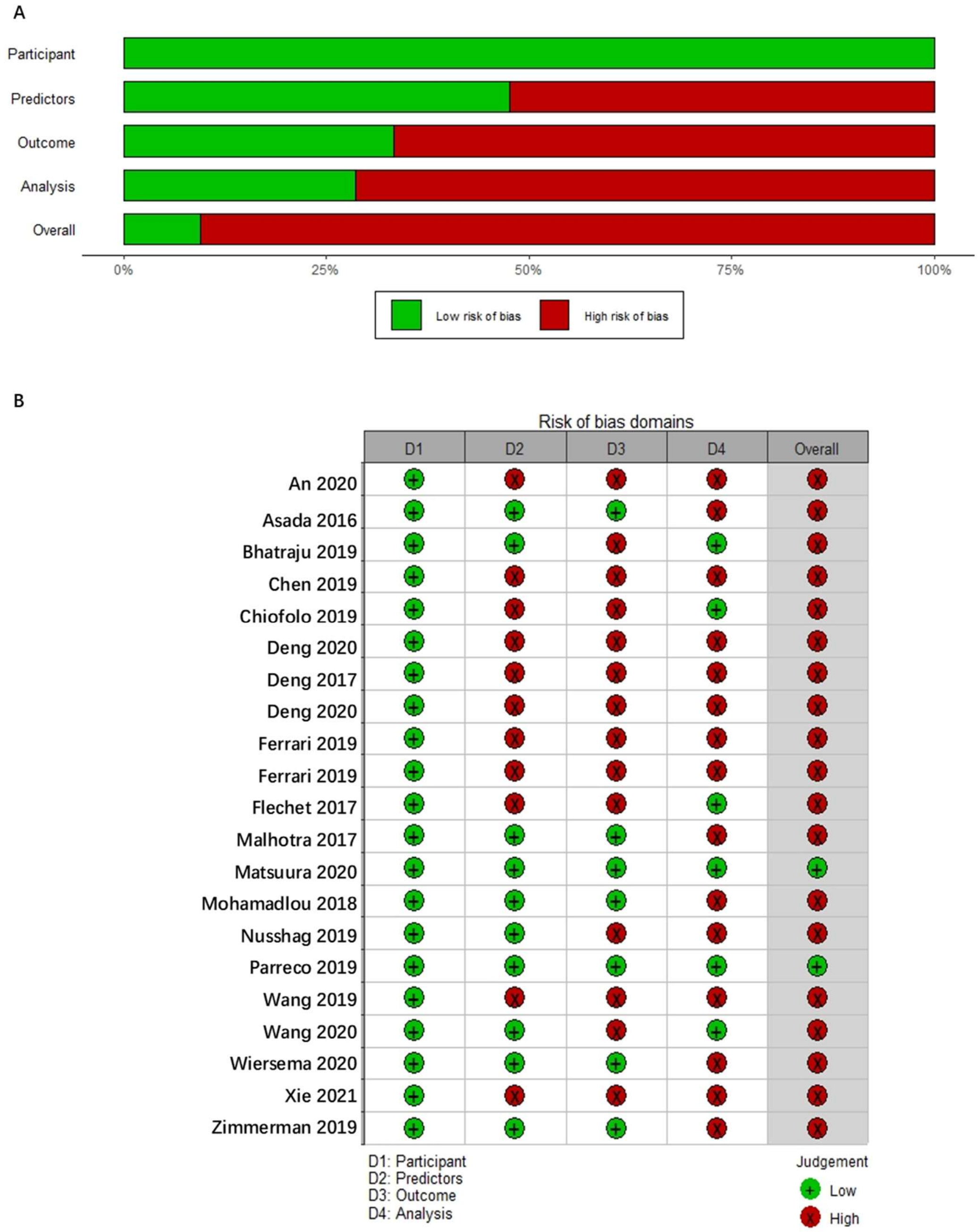

Note: (A) Summary of risk of bias assessment. (B) Traffic light plot of risk of bias assessment.

© 2023 Feng Y et al. *JAMA Network Open.*

eFigure 35. Risk of Bias Assessment Results Using PROBAST Tool for Prediction Models of AKI in General Hospitalization

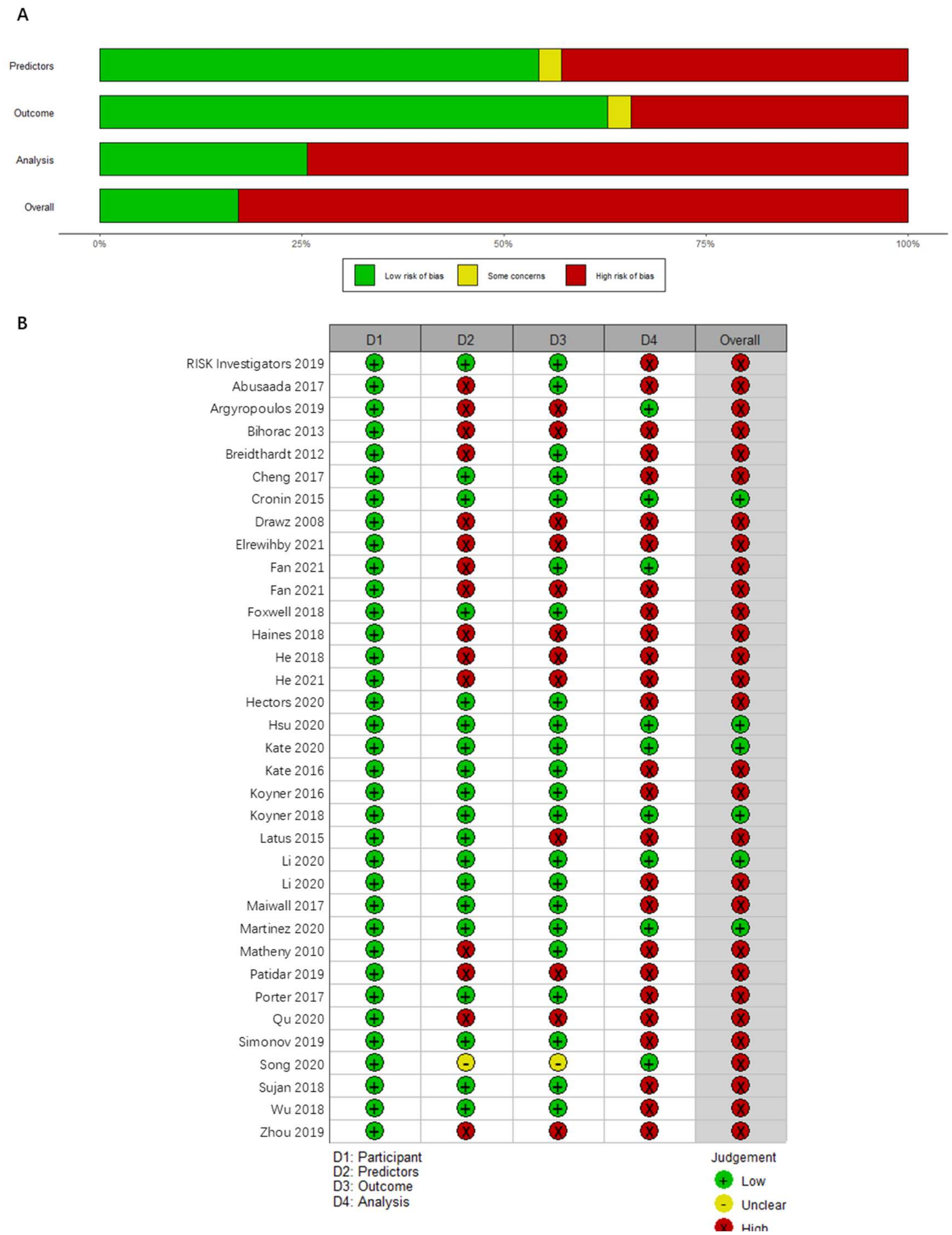

Note: (A) Summary of risk of bias assessment. (B) Traffic light plot of risk of bias assessment.

© 2023 Feng Y et al. *JAMA Network Open.*

eReferences.

1. Moons KG, Altman DG, Reitsma JB, Ioannidis JP, Macaskill P, Steyerberg EW, Vickers AJ, Ransohoff DF, Collins GS: **Transparent Reporting of a multivariable prediction model for Individual Prognosis or Diagnosis (TRIPOD): explanation and elaboration**. *Ann Intern Med* 2015, **162**(1):W1-73.
2. Hanley JA, McNeil BJ: **The meaning and use of the area under a receiver operating characteristic (ROC) curve**. *Radiology* 1982, **143**(1):29-36.
3. Reitsma JB, Glas AS, Rutjes AW, Scholten RJ, Bossuyt PM, Zwinderman AH: **Bivariate analysis of sensitivity and specificity produces informative summary measures in diagnostic reviews**. *J Clin Epidemiol* 2005, **58**(10):982-990.
4. Safari S, Baratloo A, Elfil M, Negida A: **Evidence Based Emergency Medicine; Part 4: Pre-test and Post-test Probabilities and Fagan's nomogram**. *Emerg (Tehran)* 2016, **4**(1):48-51.
5. Knapp G, Hartung J: **Improved tests for a random effects meta-regression with a single covariate**. *Stat Med* 2003, **22**(17):2693-2710.
6. Rucker G, Schwarzer G: **Beyond the forest plot: The drapery plot**. *Res Synth Methods* 2021, **12**(1):13-19.
7. Higgins JP, Thompson SG, Spiegelhalter DJ: **A re-evaluation of random-effects meta-analysis**. *J R Stat Soc Ser A Stat Soc* 2009, **172**(1):137-159.
8. Veroniki AA, Jackson D, Viechtbauer W, Bender R, Bowden J, Knapp G, Kuss O, Higgins JP, Langan D, Salanti G: **Methods to estimate the between-study variance and its uncertainty in meta-analysis**. *Res Synth Methods* 2016, **7**(1):55-79.
9. Higgins JP, Thompson SG: **Quantifying heterogeneity in a meta-analysis**. *Stat Med* 2002, **21**(11):1539-1558.
10. Baujat B, Mahe C, Pignon JP, Hill C: **A graphical method for exploring heterogeneity in meta-analyses: application to a meta-analysis of 65 trials**. *Stat Med* 2002, **21**(18):2641-2652.
11. Viechtbauer W, Cheung MW: **Outlier and influence diagnostics for meta-analysis**. *Res Synth Methods* 2010, **1**(2):112-125.
12. Peters JL, Sutton AJ, Jones DR, Abrams KR, Rushton L: **Contour-enhanced meta-analysis funnel plots help distinguish publication bias from other causes of asymmetry**. *J Clin Epidemiol* 2008, **61**(10):991-996.
13. Duval S, Tweedie R: **Trim and fill: A simple funnel-plot-based method of testing and adjusting for publication bias in meta-analysis**. *Biometrics* 2000, **56**(2):455-463.
14. Egger M, Davey Smith G, Schneider M, Minder C: **Bias in meta-analysis detected by a simple, graphical test**. *BMJ* 1997, **315**(7109):629-634.
15. Ando G, de Gregorio C, Morabito G, Trio O, Saporito F, Oreto G: **Renal function-adjusted contrast volume redefines the baseline estimation of contrast-induced acute kidney injury risk in patients undergoing primary percutaneous coronary intervention**. *Circ Cardiovasc Interv* 2014, **7**(4):465-472.
16. Brown JR, MacKenzie TA, Maddox TM, Fly J, Tsai TT, Plomondon ME, Nielson CD, Siew ED, Resnic FS, Baker CR *et al*: **Acute Kidney Injury Risk Prediction in Patients Undergoing Coronary Angiography in a National Veterans Health Administration Cohort With External Validation**. *J Am Heart Assoc* 2015, **4**(12).

© 2023 Feng Y et al. *JAMA Network Open.*

17. Chen Y-L, Fu N-K, Xu J, Yang S-C, Li S, Liu Y-Y, Cong H-L: **A simple preprocedural score for risk of contrast-induced acute kidney injury after percutaneous coronary intervention**. *Catheterization and Cardiovascular Interventions* 2014, **83**(1):E8-E16.
18. Duan C, Cao Y, Liu Y, Zhou L, Ping K, Tan MT, Tan N, Chen J, Chen P: **A New Preprocedure Risk Score for Predicting Contrast-Induced Acute Kidney Injury**. *Can J Cardiol* 2017, **33**(6):714-723.
19. Fan PC, Chen TH, Lee CC, Tsai TY, Chen YC, Chang CH: **ADVANCIS Score Predicts Acute Kidney Injury After Percutaneous Coronary Intervention for Acute Coronary Syndrome**. *Int J Med Sci* 2018, **15**(5):528-535.
20. Amal Abdel Ghani KYT: **Risk Score for Contrast Induced Nephropathy following Percutaneous Coronary Intervention**. *Saudi J Kidney Dis Transpl* 2009, **20**(2):240-245.
21. Gurm HS, Seth M, Kooiman J, Share D: **A novel tool for reliable and accurate prediction of renal complications in patients undergoing percutaneous coronary intervention**. *J Am Coll Cardiol* 2013, **61**(22):2242-2248.
22. Xun Hu, Xiao-Dong Zhuang, Yi Li, Fei-Fei Li, Yue Guo,Zhi-Min Du, and Xin-Xue Liao: **A Nomogram to Predict Contrast Induced Nephropathy in Patients Undergoing Percutaneous Coronary Intervention Is the "Anti-Aging" Agent Klotho a Candidate Predictor?** *Int Heart J* 2017, **58**: 191-196.
23. Huang C, Murugiah K, Mahajan S, Li SX, Dhruva SS, Haimovich JS, Wang Y, Schulz WL, Testani JM, Wilson FP *et al*: **Enhancing the prediction of acute kidney injury risk after percutaneous coronary intervention using machine learning techniques: A retrospective cohort study**. *PLoS Med* 2018, **15**(11):e1002703.
24. Ibrahim NE, McCarthy CP, Shrestha S, Gaggin HK, Mukai R, Magaret CA, Rhyne RF, Januzzi JL, Jr.: **A clinical, proteomics, and artificial intelligence-driven model to predict acute kidney injury in patients undergoing coronary angiography**. *Clin Cardiol* 2019, **42**(2):292-298.
25. Inohara T, Kohsaka S, Abe T, Miyata H, Numasawa Y, Ueda I, Nishi Y, Naito K, Shibata M, Hayashida K *et al*: **Development and validation of a pre-percutaneous coronary intervention risk model of contrast-induced acute kidney injury with an integer scoring system**. *Am J Cardiol* 2015, **115**(12):1636-1642.
26. Jeon J, Kim S, Yoo H, Kim K, Kim Y, Park S, Jang HR, Kim DK, Huh W, Kim YG *et al*: **Risk Prediction for Contrast-Induced Nephropathy in Cancer Patients Undergoing Computed Tomography under Preventive Measures**. *J Oncol* 2019, **2019**:8736163.
27. Ji L, Su X, Qin W, Mi X, Liu F, Tang X, Li Z, Yang L: **Novel risk score of contrast-induced nephropathy after percutaneous coronary intervention**. *Nephrology (Carlton)* 2015, **20**(8):544-551.
28. Dan Lian YL, Yuan-hui Liu, Hua-long Li, Chong-yang Duan, and Dan-qing Yu: **Pre-Procedural Risk Score of Contrast-Induced Nephropathy in Elderly Patients Undergoing Elective Coronary Angiography**. *Int Heart J* 2017, **58**:197-204.
29. Lin KY, Zheng WP, Bei WJ, Chen SQ, Islam SM, Liu Y, Xue L, Tan N, Chen JY: **A novel risk score model for prediction of contrast-induced nephropathy after emergent percutaneous coronary intervention**. *Int J Cardiol* 2017, **230**:402-412.
30. Liu L, Liu J, Lei L, Wang B, Sun G, Guo Z, He Y, Song F, Lun Z, Liu B *et al*: **A prediction model of contrast-associated acute kidney injury in patients with**

hypoalbuminemia undergoing coronary angiography. *BMC Cardiovasc Disord* 2020, **20**(1):399.

31. Liu Y, Chen S, Ye J, Xian Y, Wang X, Xuan J, Tan N, Li Q, Chen J, Ni Z: **Random forest for prediction of contrast-induced nephropathy following coronary angiography**. *Int J Cardiovasc Imaging* 2020, **36**(6):983-991.
32. Sun L, Zhu W, Chen X, Jiang J, Ji Y, Liu N, Xu Y, Zhuang Y, Sun Z, Wang Q *et al*: **Machine Learning to Predict Contrast-Induced Acute Kidney Injury in Patients With Acute Myocardial Infarction**. *Front Med (Lausanne)* 2020, **7**:592007.
33. Tsai TT, Patel UD, Chang TI, Kennedy KF, Masoudi FA, Matheny ME, Kosiborod M, Amin AP, Weintraub WS, Curtis JP *et al*: **Validated contemporary risk model of acute kidney injury in patients undergoing percutaneous coronary interventions: insights from the National Cardiovascular Data Registry Cath-PCI Registry**. *J Am Heart Assoc* 2014, **3**(6):e001380.
34. Yin WJ, Yi YH, Guan XF, Zhou LY, Wang JL, Li DY, Zuo XC: **Preprocedural Prediction Model for Contrast-Induced Nephropathy Patients**. *J Am Heart Assoc* 2017, **6**(2).
35. Zambetti BR, Thomas F, Hwang I, Brown AC, Chumpia M, Ellis RT, Naik D, Khouzam RN, Ibebuogu UN, Reed GL: **A web-based tool to predict acute kidney injury in patients with ST-elevation myocardial infarction: Development, internal validation and comparison**. *PLoS One* 2017, **12**(7):e0181658.
36. Zhou X, Sun Z, Zhuang Y, Jiang J, Liu N, Zang X, Chen X, Li H, Cao H, Sun L *et al*: **Development and Validation of Nomogram to Predict Acute Kidney Injury in Patients with Acute Myocardial Infarction Treated Invasively**. *Sci Rep* 2018, **8**(1):9769.
37. Yao ZF, Shen H, Tang MN, Yan Y, Ge JB: **A novel risk assessment model of contrast-induced nephropathy after percutaneous coronary intervention in patients with diabetes**. *Basic Clin Pharmacol Toxicol* 2021, **128**(2):305-314.
38. Mehran R, Aymong ED, Nikolsky E, Lasic Z, Iakovou I, Fahy M, Mintz GS, Lansky AJ, Moses JW, Stone GW *et al*: **A simple risk score for prediction of contrast-induced nephropathy after percutaneous coronary intervention: development and initial validation**. *J Am Coll Cardiol* 2004, **44**(7):1393-1399.
39. Ando G, Morabito G, de Gregorio C, Trio O, Saporito F, Oreto G: **Age, glomerular filtration rate, ejection fraction, and the AGEF score predict contrast-induced nephropathy in patients with acute myocardial infarction undergoing primary percutaneous coronary intervention**. *Catheter Cardiovasc Interv* 2013, **82**(6):878-885.
40. Tziakas D, Chalikias G, Stakos D, Apostolakis S, Adina T, Kikas P, Alexoudis A, Passadakis P, Thodis E, Vargemezis V *et al*: **Development of an easily applicable risk score model for contrast-induced nephropathy prediction after percutaneous coronary intervention: a novel approach tailored to current practice**. *Int J Cardiol* 2013, **163**(1):46-55.
41. Jeon N, Staley B, Henriksen C, Lipori GP, Winterstein AG: **Development and validation of an automated algorithm for identifying patients at higher risk for drug-induced acute kidney injury**. *Am J Health Syst Pharm* 2019, **76**(10):654-666.

© 2023 Feng Y et al. *JAMA Network Open.*

42. Shveta S. Motwani GMM, Benjamin D. Humphreys, Ann H. Partridge, Sushrut S. Waikar, and Gary C. Curhan: **Development and Validation of a Risk Prediction Model for Acute Kidney Injury After the First Course of Cisplatin**. *JOURNAL OF CLINICAL ONCOLOGY* 2018, **36**(7):682-688.
43. Collaborative ST: **Prognostic model to predict postoperative acute kidney injury in patients undergoing major gastrointestinal surgery based on a national prospective observational cohort study**. *BJS Open* 2018, **2**(6):400-410.
44. Adhikari L, Ozrazgat-Baslanti T, Ruppert M, Madushani R, Paliwal S, Hashemighouchani H, Zheng F, Tao M, Lopes JM, Li X *et al*: **Improved predictive models for acute kidney injury with IDEA: Intraoperative Data Embedded Analytics**. *PLoS One* 2019, **14**(4):e0214904.
45. Al-Jefri M, Lee J, James M: **Predicting Acute Kidney Injury after Surgery**. *Annu Int Conf IEEE Eng Med Biol Soc* 2020, **2020**:5606-5609.
46. Antunes PE, de Oliveira JF, Antunes MJ: **Risk-prediction for postoperative major morbidity in coronary surgery**. *Eur J Cardiothorac Surg* 2009, **35**(5):760-766; discussion 766-767.
47. Bell S, Dekker FW, Vadiveloo T, Marwick C, Deshmukh H, Donnan PT, Van Diepen M: **Risk of postoperative acute kidney injury in patients undergoing orthopaedic surgery--development and validation of a risk score and effect of acute kidney injury on survival: observational cohort study**. *BMJ* 2015, **351**:h5639.
48. Berg KS, Stenseth R, Wahba A, Pleym H, Videm V: **How can we best predict acute kidney injury following cardiac surgery?: a prospective observational study**. *Eur J Anaesthesiol* 2013, **30**(11):704-712.
49. Bihorac A, Ozrazgat-Baslanti T, Ebadi A, Motaei A, Madkour M, Pardalos PM, Lipori G, Hogan WR, Efron PA, Moore F *et al*: **MySurgeryRisk: Development and Validation of a Machine-learning Risk Algorithm for Major Complications and Death After Surgery**. *Ann Surg* 2019, **269**(4):652-662.
50. Birnie K, Verheyden V, Pagano D, Bhabra M, Tilling K, Sterne JA, Murphy GJ, Collaborators UAiCS: **Predictive models for kidney disease: improving global outcomes (KDIGO) defined acute kidney injury in UK cardiac surgery**. *Crit Care* 2014, **18**(6):606.
51. Che M, Wang X, Liu S, Xie B, Xue S, Yan Y, Zhu M, Lu R, Qian J, Ni Z *et al*: **A Clinical Score to Predict Severe Acute Kidney Injury in Chinese Patients after Cardiac Surgery**. *Nephron* 2019, **142**(4):291-300.
52. Chen Z, Chen L, Yao G, Yang W, Yang K, Xiong C: **Novel Blood Cytokine-Based Model for Predicting Severe Acute Kidney Injury and Poor Outcomes After Cardiac Surgery**. *J Am Heart Assoc* 2020, **9**(22):e018004.
53. Coulson T, Bailey M, Pilcher D, Reid CM, Seevanayagam S, Williams-Spence J, Bellomo R: **Predicting Acute Kidney Injury After Cardiac Surgery Using a Simpler Model**. *J Cardiothorac Vasc Anesth* 2021, **35**(3):866-873.
54. Dedemoglu M, Tuysuz ME: **Risk estimation model for acute kidney injury defined by KDIGO classification after heart valve replacement surgery**. *Gen Thorac Cardiovasc Surg* 2020, **68**(9):922-931.

© 2023 Feng Y et al. *JAMA Network Open.*

55. Du Y, Wang XZ, Wu WD, Shi HP, Yang XJ, Wu WJ, Chen SX: **Predicting the Risk of Acute Kidney Injury in Patients After Percutaneous Coronary Intervention (PCI) or Cardiopulmonary Bypass (CPB) Surgery: Development and Assessment of a Nomogram Prediction Model**. *Med Sci Monit* 2021, **27**:e929791.
56. Grimm JC, Lui C, Kilic A, Valero V, 3rd, Sciortino CM, Whitman GJ, Shah AS: **A risk score to predict acute renal failure in adult patients after lung transplantation**. *Ann Thorac Surg* 2015, **99**(1):251-257.
57. Guan C, Li C, Xu L, Zhen L, Zhang Y, Zhao L, Zhou B, Che L, Wang Y, Xu Y: **Risk factors of cardiac surgery-associated acute kidney injury: development and validation of a perioperative predictive nomogram**. *J Nephrol* 2019, **32**(6):937-945.
58. Hofer IS, Lee C, Gabel E, Baldi P, Cannesson M: **Development and validation of a deep neural network model to predict postoperative mortality, acute kidney injury, and reintubation using a single feature set**. *NPJ Digit Med* 2020, **3**:58.
59. Hu P, Chen Y, Wu Y, Song L, Zhang L, Li Z, Fu L, Liu S, Ye Z, Shi W *et al*: **Development and validation of a model for predicting acute kidney injury after cardiac surgery in patients of advanced age**. *J Card Surg* 2021, **36**(3):806-814.
60. Hu XY, Liu DW, Qiao YJ, Zheng X, Duan JY, Pan SK, Liu ZS: **Development and Validation of a Nomogram Model to Predict Acute Kidney Disease After Nephrectomy in Patients with Renal Cell Carcinoma**. *Cancer Manag Res* 2020, **12**:11783-11791.
61. Jiang W, Teng J, Xu J, Shen B, Wang Y, Fang Y, Zou Z, Jin J, Zhuang Y, Liu L *et al*: **Dynamic Predictive Scores for Cardiac Surgery-Associated Acute Kidney Injury**. *J Am Heart Assoc* 2016, **5**(8).
62. Jorge-Monjas P, Bustamante-Munguira J, Lorenzo M, Heredia-Rodriguez M, Fierro I, Gomez-Sanchez E, Hernandez A, Alvarez FJ, Bermejo-Martin JF, Gomez-Pesquera E *et al*: **Predicting cardiac surgery-associated acute kidney injury: The CRATE score**. *J Crit Care* 2016, **31**(1):130-138.
63. Kalisvaart M, Schlegel A, Umbro I, de Haan JE, Polak WG, JN IJ, Mirza DF, Perera MTP, Isaac JR, Ferguson J *et al*: **The AKI Prediction Score: a new prediction model for acute kidney injury after liver transplantation**. *HPB (Oxford)* 2019, **21**(12):1707-1717.
64. Kashani K, Steuernagle JHt, Akhoundi A, Alsara A, Hanson AC, Kor DJ: **Vascular Surgery Kidney Injury Predictive Score: A Historical Cohort Study**. *J Cardiothorac Vasc Anesth* 2015, **29**(6):1588-1595.
65. Kim JM, Jo YY, Na SW, Kim SI, Choi YS, Kim NO, Park JE, Koh SO: **The predictors for continuous renal replacement therapy in liver transplant recipients**. *Transplant Proc* 2014, **46**(1):184-191.
66. Kim MY, Jang HR, Huh W, Kim YG, Kim DJ, Lee YT, Oh HY, Eun Lee J: **Incidence, risk factors, and prediction of acute kidney injury after off-pump coronary artery bypass grafting**. *Ren Fail* 2011, **33**(3):316-322.
67. Ko S, Jo C, Chang CB, Lee YS, Moon YW, Youm JW, Han HS, Lee MC, Lee H, Ro DH: **A web-based machine-learning algorithm predicting postoperative acute kidney injury after total knee arthroplasty**. *Knee Surg Sports Traumatol Arthrosc* 2020.

© 2023 Feng Y et al. *JAMA Network Open.*

68. Lee HC, Yoon HK, Nam K, Cho YJ, Kim TK, Kim WH, Bahk JH: **Derivation and Validation of Machine Learning Approaches to Predict Acute Kidney Injury after Cardiac Surgery**. *J Clin Med* 2018, **7**(10).
69. Lee HC, Yoon SB, Yang SM, Kim WH, Ryu HG, Jung CW, Suh KS, Lee KH: **Prediction of Acute Kidney Injury after Liver Transplantation: Machine Learning Approaches vs. Logistic Regression Model**. *J Clin Med* 2018, **7**(11).
70. Legrand M, Pirracchio R, Rosa A, Petersen ML, Van der Laan M, Fabiani JN, Fernandez-gerlinger MP, Podglajen I, Safran D, Cholley B *et al*: **Incidence, risk factors and prediction of post-operative acute kidney injury following cardiac surgery for active infective endocarditis: an observational study**. *Crit Care* 2013, **17**(5):R220.
71. Lei L, Wang Y, Xue Q, Tong J, Zhou CM, Yang JJ: **A comparative study of machine learning algorithms for predicting acute kidney injury after liver cancer resection**. *PeerJ* 2020, **8**:e8583.
72. Lei VJ, Luong T, Shan E, Chen X, Neuman MD, Eneanya ND, Polsky DE, Volpp KG, Fleisher LA, Holmes JH *et al*: **Risk Stratification for Postoperative Acute Kidney Injury in Major Noncardiac Surgery Using Preoperative and Intraoperative Data**. *JAMA Netw Open* 2019, **2**(12):e1916921.
73. Li Y, Xu J, Wang Y, Zhang Y, Jiang W, Shen B, Ding X: **A novel machine learning algorithm, Bayesian networks model, to predict the high-risk patients with cardiac surgery-associated acute kidney injury**. *Clin Cardiol* 2020, **43**(7):752-761.
74. Lin H, Hou J, Tang H, Chen K, Sun H, Zheng Z, Hu S: **A novel nomogram to predict perioperative acute kidney injury following isolated coronary artery bypass grafting surgery with impaired left ventricular ejection fraction**. *BMC Cardiovasc Disord* 2020, **20**(1):517.
75. Liu X, Ye Y, Mi Q, Huang W, He T, Huang P, Xu N, Wu Q, Wang A, Li Y *et al*: **A Predictive Model for Assessing Surgery-Related Acute Kidney Injury Risk in Hypertensive Patients: A Retrospective Cohort Study**. *PLoS One* 2016, **11**(11):e0165280.
76. Martini A, Sfakianos JP, Paulucci DJ, Abaza R, Eun DD, Bhandari A, Hemal AK, Badani KK: **Predicting acute kidney injury after robot-assisted partial nephrectomy: Implications for patient selection and postoperative management**. *Urol Oncol* 2019, **37**(7):445-451.
77. McBride WT, Kurth MJ, McLean G, Domanska A, Lamont JV, Maguire D, Watt J, Fitzgerald P, Young I, Joseph J *et al*: **Stratifying risk of acute kidney injury in pre and post cardiac surgery patients using a novel biomarker-based algorithm and clinical risk score**. *Sci Rep* 2019, **9**(1):16963.
78. Metzger J, Mullen W, Husi H, Stalmach A, Herget-Rosenthal S, Groesdonk HV, Mischak H, Klingele M: **Acute kidney injury prediction in cardiac surgery patients by a urinary peptide pattern: a case-control validation study**. *Crit Care* 2016, **20**(1):157.
79. Meyer A, Zverinski D, Pfahringer B, Kempfert J, Kuehne T, Sündermann SH, Stamm C, Hofmann T, Falk V, Eickhoff C: **Machine learning for real-time prediction of complications in critical care: a retrospective study**. *The Lancet Respiratory Medicine* 2018, **6**(12):905-914.

© 2023 Feng Y et al. *JAMA Network Open.*

80. Nah CW, Ti LK, Liu W, Ng RR, Shen L, Chew ST: **A clinical score to predict acute kidney injury after cardiac surgery in a Southeast-Asian population**. *Interact Cardiovasc Thorac Surg* 2016, **23**(5):757-761.
81. Ortega-Loubon C, Fernandez-Molina M, Paneda-Delgado L, Jorge-Monjas P, Carrascal Y: **Predictors of Postoperative Acute Kidney Injury after Coronary Artery Bypass Graft Surgery**. *Braz J Cardiovasc Surg* 2018, **33**(4):323-329.
82. Pannu N, Graham M, Klarenbach S, Meyer S, Kieser T, Hemmelgarn B, Ye F, James M, Investigators A, the Alberta Kidney Disease N: **A new model to predict acute kidney injury requiring renal replacement therapy after cardiac surgery**. *CMAJ* 2016, **188**(15):1076-1083.
83. Paparella D, Guida P, Mazzei V, Carbone C, Speziale G, Fanelli V, Zaccaria S, Labriola C, Scrascia G: **Hemoglobin and renal replacement therapy after cardiopulmonary bypass surgery: a predictive score from the Cardiac Surgery Registry of Puglia**. *Int J Cardiol* 2014, **176**(3):866-873.
84. Penny-Dimri JC, Bergmeir C, Reid CM, Williams-Spence J, Cochrane AD, Smith JA: **Machine Learning Algorithms for Predicting and Risk Profiling of Cardiac Surgery-Associated Acute Kidney Injury**. *Semin Thorac Cardiovasc Surg* 2021, **33**(3):735-745.
85. Ramonell KM, Fang, S., Perez, S. D., Srinivasan, J. K., Sullivan, P. S., Galloway, J. R., Staley, C. A., Lin, E., Sharma, J., Sweeney, J. F., & Shaffer, V. O.: **Development and Validation of a Risk Calculator for Renal Complications after Colorectal Surgery Using the National Surgical Quality Improvement Program Participant Use Files**. *The American surgeon* 2016, **82**(12):1244-1249.
86. Rank N, Pfahringer B, Kempfert J, Stamm C, Kuhne T, Schoenrath F, Falk V, Eickhoff C, Meyer A: **Deep-learning-based real-time prediction of acute kidney injury outperforms human predictive performance**. *NPJ Digit Med* 2020, **3**:139.
87. Rueggeberg A, Boehm S, Napieralski F, Mueller AR, Neuhaus P, Falke KJ, Gerlach H: **Development of a risk stratification model for predicting acute renal failure in orthotopic liver transplantation recipients**. *Anaesthesia* 2008, **63**(11):1174-1180.
88. Simonini M, Lanzani C, Bignami E, Casamassima N, Frati E, Meroni R, Messaggio E, Alfieri O, Hamlyn J, Body SC *et al*: **A new clinical multivariable model that predicts postoperative acute kidney injury: impact of endogenous ouabain**. *Nephrol Dial Transplant* 2014, **29**(9):1696-1701.
89. Slankamenac K, Beck-Schimmer B, Breitenstein S, Puhan MA, Clavien PA: **Novel prediction score including pre- and intraoperative parameters best predicts acute kidney injury after liver surgery**. *World J Surg* 2013, **37**(11):2618-2628.
90. Slankamenac K, Breitenstein S, Held U, Beck-Schimmer B, Puhan MA, Clavien PA: **Development and validation of a prediction score for postoperative acute renal failure following liver resection**. *Ann Surg* 2009, **250**(5):720-728.
91. Thakar CV, Arrigain S, Worley S, Yared JP, Paganini EP: **A clinical score to predict acute renal failure after cardiac surgery**. *J Am Soc Nephrol* 2005, **16**(1):162-168.
92. Tian Y, Diao X, Wang Y, Wang C, Wang W, Xu X, Gao Y, Wang S, Liu J, Ji B *et al*: **Prediction Scores for Any-Stage and Stage-3 Acute Kidney Injury After Adult Cardiac Surgery in a Chinese Population**. *J Cardiothorac Vasc Anesth* 2021, **35**(10):3001-3009.

© 2023 Feng Y et al. *JAMA Network Open.*

93. Trongtrakul K, Patumanond J, Kongsayreepong S, Morakul S, Pipanmekaporn T, Akaraborworn O, Poopipatpab S: **Acute kidney injury risk prediction score for critically-ill surgical patients**. *BMC Anesthesiol* 2020, **20**(1):140.

94. Tseng PY, Chen YT, Wang CH, Chiu KM, Peng YS, Hsu SP, Chen KL, Yang CY, Lee OK: **Prediction of the development of acute kidney injury following cardiac surgery by machine learning**. *Crit Care* 2020, **24**(1):478.

95. Xue B, Li D, Lu C, King CR, Wildes T, Avidan MS, Kannampallil T, Abraham J: **Use of Machine Learning to Develop and Evaluate Models Using Preoperative and Intraoperative Data to Identify Risks of Postoperative Complications**. *JAMA Netw Open* 2021, **4**(3):e212240.

96. Yayac M, Aman ZS, Rondon AJ, Tan TL, Courtney PM, Purtill JJ: **Risk Factors and Effect of Acute Kidney Injury on Outcomes Following Total Hip and Knee Arthroplasty**. *J Arthroplasty* 2021, **36**(1):331-338.

97. Zhang P, Guan C, Li C, Zhu Z, Zhang W, Luan H, Zhou B, Man X, Che L, Wang Y *et al*: **A visual risk assessment tool for acute kidney injury after intracranial aneurysm clipping surgery**. *Ren Fail* 2020, **42**(1):1093-1099.

98. Zhou C, Wang R, Jiang W, Zhu J, Liu Y, Zheng J, Wang X, Shang W, Sun L: **Machine learning for the prediction of acute kidney injury and paraplegia after thoracoabdominal aortic aneurysm repair**. *J Card Surg* 2020, **35**(1):89-99.

99. Brown JR, Cochran RP, Leavitt BJ, Dacey LJ, Ross CS, MacKenzie TA, Kunzelman KS, Kramer RS, Hernandez F, Jr., Helm RE *et al*: **Multivariable prediction of renal insufficiency developing after cardiac surgery**. *Circulation* 2007, **116**(11 Suppl):I139-143.

100. Mehta RH, Grab JD, O'Brien SM, Bridges CR, Gammie JS, Haan CK, Ferguson TB, Peterson ED, Society of Thoracic Surgeons National Cardiac Surgery Database I: **Bedside tool for predicting the risk of postoperative dialysis in patients undergoing cardiac surgery**. *Circulation* 2006, **114**(21):2208-2216; quiz 2208.

101. Wijeysundera DN, Karkouti K, Dupuis JY, Rao V, Chan CT, Granton JT, Beattie WS: **Derivation and validation of a simplified predictive index for renal replacement therapy after cardiac surgery**. *JAMA* 2007, **297**(16):1801-1809.

102. Palomba H, de Castro I, Neto AL, Lage S, Yu L: **Acute kidney injury prediction following elective cardiac surgery: AKICS Score**. *Kidney Int* 2007, **72**(5):624-631.

103. Demirjian S, Schold JD, Navia J, Mastracci TM, Paganini EP, Yared JP, Bashour CA: **Predictive models for acute kidney injury following cardiac surgery**. *Am J Kidney Dis* 2012, **59**(3):382-389.

104. Kheterpal S, Tremper KK, Heung M, Rosenberg AL, Englesbe M, Shanks AM, Campbell DA, Jr.: **Development and validation of an acute kidney injury risk index for patients undergoing general surgery: results from a national data set**. *Anesthesiology* 2009, **110**(3):505-515.

105. Ng SY, Sanagou M, Wolfe R, Cochrane A, Smith JA, Reid CM: **Prediction of acute kidney injury within 30 days of cardiac surgery**. *J Thorac Cardiovasc Surg* 2014, **147**(6):1875-1883, 1883 e1871.

© 2023 Feng Y et al. *JAMA Network Open.*

106. Kim WH, Lee SM, Choi JW, Kim EH, Lee JH, Jung JW, Ahn JH, Sung KI, Kim CS, Cho HS: **Simplified clinical risk score to predict acute kidney injury after aortic surgery**. *J Cardiothorac Vasc Anesth* 2013, **27**(6):1158-1166.

107. An S, Luo H, Wang J, Gong Z, Tian Y, Liu X, Ma J, Jiang R: **An acute kidney injury prediction nomogram based on neurosurgical intensive care unit profiles**. *Ann Transl Med* 2020, **8**(5):194.

108. Asada T, Isshiki R, Hayase N, Sumida M, Inokuchi R, Noiri E, Nangaku M, Yahagi N, Doi K: **Impact of clinical context on acute kidney injury biomarker performances: differences between neutrophil gelatinase-associated lipocalin and L-type fatty acid-binding protein**. *Sci Rep* 2016, **6**:33077.

109. Bhatraju PK, Zelnick LR, Katz R, Mikacenic C, Kosamo S, Hahn WO, Dmyterko V, Kestenbaum B, Christiani DC, Liles WC *et al*: **A Prediction Model for Severe AKI in Critically Ill Adults That Incorporates Clinical and Biomarker Data**. *Clin J Am Soc Nephrol* 2019, **14**(4):506-514.

110. Chen Y, Feng F, Li M, Chang X, Wei B, Dong C: **Development of a risk stratification-based model for prediction of acute kidney injury in critically ill patients**. *Medicine (Baltimore)* 2019, **98**(33):e16867.

111. Chiofolo C, Chbat N, Ghosh E, Eshelman L, Kashani K: **Automated Continuous Acute Kidney Injury Prediction and Surveillance: A Random Forest Model**. *Mayo Clin Proc* 2019, **94**(5):783-792.

112. Deng F, Peng M, Li J, Chen Y, Zhang B, Zhao S: **Nomogram to predict the risk of septic acute kidney injury in the first 24 h of admission: an analysis of intensive care unit data**. *Ren Fail* 2020, **42**(1):428-436.

113. Deng Y, Chi R, Chen S, Ye H, Yuan J, Wang L, Zhai Y, Gao L, Zhang D, Hu L *et al*: **Evaluation of clinically available renal biomarkers in critically ill adults: a prospective multicenter observational study**. *Crit Care* 2017, **21**(1):46.

114. Deng Y, Ma J, Hou Y, Zhou D, Hou T, Li J, Liang S, Tan N, Chen C: **Combining Serum Cystatin C and Urinary N-Acetyl-Beta-D-Glucosaminidase Improves the Precision for Acute Kidney Injury Diagnosis after Resection of Intracranial Space-Occupying Lesions**. *Kidney Blood Press Res* 2020, **45**(1):142-156.

115. Ferrari F, Puci MV, Ferraro OE, Romero-Gonzalez G, Husain-Syed F, Rizo-Topete L, Senzolo M, Lorenzin A, Muraro E, Baracca A *et al*: **Development and validation of quick Acute Kidney Injury-score (q-AKI) to predict acute kidney injury at admission to a multidisciplinary intensive care unit**. *PLoS One* 2019, **14**(6):e0217424.

116. Ferrari F, Romero-Gonzalez G, Topete LR, Senzolo M, Lorenzin A, Husain-Syed F, Puci MV, Ferraro OE, Muraro E, Serrano-Soto M *et al*: **Routine Adoption of Urinary [IGFBP7][TIMP-2] to Assess Acute Kidney Injury at Any Stage 12 hours After Intensive Care Unit Admission: a Prospective Cohort Study**. *Sci Rep* 2019, **9**(1):16484.

117. Flechet M, Guiza F, Schetz M, Wouters P, Vanhorebeek I, Derese I, Gunst J, Spriet I, Casaer M, Van den Berghe G *et al*: **AKIpredictor, an online prognostic calculator for acute kidney injury in adult critically ill patients: development, validation and comparison to serum neutrophil gelatinase-associated lipocalin**. *Intensive Care Med* 2017, **43**(6):764-773.

© 2023 Feng Y et al. *JAMA Network Open.*

118. Malhotra R, Kashani KB, Macedo E, Kim J, Bouchard J, Wynn S, Li G, Ohno-Machado L, Mehta R: **A risk prediction score for acute kidney injury in the intensive care unit**. *Nephrol Dial Transplant* 2017, **32**(5):814-822.
119. Matsuura R, Iwagami M, Moriya H, Ohtake T, Hamasaki Y, Nangaku M, Doi K, Kobayashi S, Noiri E: **A Simple Scoring Method for Predicting the Low Risk of Persistent Acute Kidney Injury in Critically Ill Adult Patients**. *Sci Rep* 2020, **10**(1):5726.
120. Mohamadlou H, Lynn-Palevsky A, Barton C, Chettipally U, Shieh L, Calvert J, Saber NR, Das R: **Prediction of Acute Kidney Injury With a Machine Learning Algorithm Using Electronic Health Record Data**. *Can J Kidney Health Dis* 2018, **5**:2054358118776326.
121. Nusshag C, Rupp C, Schmitt F, Krautkramer E, Speer C, Kalble F, Tamulyte S, Bruckner T, Zeier M, Reiser J *et al*: **Cell Cycle Biomarkers and Soluble Urokinase-Type Plasminogen Activator Receptor for the Prediction of Sepsis-Induced Acute Kidney Injury Requiring Renal Replacement Therapy: A Prospective, Exploratory Study**. *Crit Care Med* 2019, **47**(12):e999-e1007.
122. Parreco J, Soe-Lin H, Parks JJ, Byerly S, Chatoor M, Buicko JL, Namias N, Rattan R: **Comparing Machine Learning Algorithms for Predicting Acute Kidney Injury**. *Am Surg* 2019, **85**(7):725-729.
123. Wang Q, Tang Y, Zhou J, Qin W: **A prospective study of acute kidney injury in the intensive care unit: development and validation of a risk prediction model**. *J Transl Med* 2019, **17**(1):359.
124. Wang Y, Wei Y, Yang H, Li J, Zhou Y, Wu Q: **Utilizing imbalanced electronic health records to predict acute kidney injury by ensemble learning and time series model**. *BMC Med Inform Decis Mak* 2020, **20**(1):238.
125. Wiersema R, Koeze J, Eck RJ, Kaufmann T, Hiemstra B, Koster G, Franssen CFM, Vaara ST, Keus F, Van der Horst ICC: **Clinical examination findings as predictors of acute kidney injury in critically ill patients**. *Acta Anaesthesiol Scand* 2020, **64**(1):69-74.
126. Xie Y, Zhang Y, Tian R, Jin W, Du J, Zhou Z, Wang R: **A prediction model of sepsis-associated acute kidney injury based on antithrombin III**. *Clin Exp Med* 2021, **21**(1):89-100.
127. Zimmerman LP, Reyfman PA, Smith ADR, Zeng Z, Kho A, Sanchez-Pinto LN, Luo Y: **Early prediction of acute kidney injury following ICU admission using a multivariate panel of physiological measurements**. *BMC Med Inform Decis Mak* 2019, **19**(Suppl 1):16.
128. Risk I: **Risk prediction for acute kidney injury in acute medical admissions in the UK**. *QJM* 2019, **112**(3):197-205.
129. Abusaada K, Yuan C, Sabzwari R, Butt K, Maqsood A: **Development of a novel score to predict the risk of acute kidney injury in patient with acute myocardial infarction**. *J Nephrol* 2017, **30**(3):419-425.
130. Argyropoulos A, Townley S, Upton PM, Dickinson S, Pollard AS: **Identifying on admission patients likely to develop acute kidney injury in hospital**. *BMC Nephrol* 2019, **20**(1):56.

© 2023 Feng Y et al. *JAMA Network Open.*

131. Bihorac A, Baslanti TO, Cuenca AG, Hobson CE, Ang D, Efron PA, Maier RV, Moore FA, Moldawer LL: **Acute kidney injury is associated with early cytokine changes after trauma**. *J Trauma Acute Care Surg* 2013, **74**(4):1005-1013.
132. Breidthardt T, Christ-Crain M, Stolz D, Bingisser R, Drexler B, Klima T, Balmelli C, Schuetz P, Haaf P, Scharer M *et al*: **A combined cardiorenal assessment for the prediction of acute kidney injury in lower respiratory tract infections**. *Am J Med* 2012, **125**(2):168-175.
133. Cheng P WL, Hu Y, Liu M.: **Predicting Inpatient Acute Kidney Injury over Different Time Horizons: How Early and Accurate?** *AMIA Annu Symp Proc* 2018, **2017**:10.
134. Cronin RM, VanHouten JP, Siew ED, Eden SK, Fihn SD, Nielson CD, Peterson JF, Baker CR, Ikizler TA, Speroff T *et al*: **National Veterans Health Administration inpatient risk stratification models for hospital-acquired acute kidney injury**. *J Am Med Inform Assoc* 2015, **22**(5):1054-1071.
135. Drawz PE, Miller RT, Sehgal AR: **Predicting hospital-acquired acute kidney injury--a case-controlled study**. *Ren Fail* 2008, **30**(9):848-855.
136. Elrewihby W, Kasem H, Raghib A, Salah Y, Kora M: **Developing and validating a risk score model for prediction of acute kidney injury in non-ICU hospitalized patients**. *Clin Nephrol* 2021, **95**(4):182-188.
137. Fan C, Ding X, Song Y: **A new prediction model for acute kidney injury in patients with sepsis**. *Ann Palliat Med* 2021, **10**(2):1772-1778.
138. Fan T, Wang H, Wang J, Wang W, Guan H, Zhang C: **Nomogram to predict the risk of acute kidney injury in patients with diabetic ketoacidosis: an analysis of the MIMIC-III database**. *BMC Endocr Disord* 2021, **21**(1):37.
139. Phillips AO, Foxwell DA, Pradhan S, Zouwail S, Rainer TH: **Derivation of a prediction model for emergency department acute kidney injury**. *Am J Emerg Med* 2021, **40**:64-69.
140. Haines RW, Lin SP, Hewson R, Kirwan CJ, Torrance HD, O'Dwyer MJ, West A, Brohi K, Pearse RM, Zolfaghari P *et al*: **Acute Kidney Injury in Trauma Patients Admitted to Critical Care: Development and Validation of a Diagnostic Prediction Model**. *Sci Rep* 2018, **8**(1):3665.
141. He J, Hu Y, Zhang X, Wu L, Waitman LR, Liu M: **Multi-perspective predictive modeling for acute kidney injury in general hospital populations using electronic medical records**. *JAMIA Open* 2019, **2**(1):115-122.
142. He L, Zhang Q, Li Z, Shen L, Zhang J, Wang P, Wu S, Zhou T, Xu Q, Chen X *et al*: **Incorporation of Urinary Neutrophil Gelatinase-Associated Lipocalin and Computed Tomography Quantification to Predict Acute Kidney Injury and In-Hospital Death in COVID-19 Patients**. *Kidney Dis (Basel)* 2021, **7**(2):120-130.
143. Hectors SJ, Riyahi S, Dev H, Krishnan K, Margolis DJA, Prince MR: **Multivariate analysis of CT imaging, laboratory, and demographical features for prediction of acute kidney injury in COVID-19 patients: a Bi-centric analysis**. *Abdom Radiol (NY)* 2021, **46**(4):1651-1658.
144. Hsu CN, Liu CL, Tain YL, Kuo CY, Lin YC: **Machine Learning Model for Risk Prediction of Community-Acquired Acute Kidney Injury Hospitalization From**

© 2023 Feng Y et al. *JAMA Network Open.*

Electronic Health Records: Development and Validation Study. *J Med Internet Res* 2020, **22**(8):e16903.

145. Kate RJ, Pearce N, Mazumdar D, Nilakantan V: **A continual prediction model for inpatient acute kidney injury**. *Comput Biol Med* 2020, **116**:103580.
146. Kate RJ, Perez RM, Mazumdar D, Pasupathy KS, Nilakantan V: **Prediction and detection models for acute kidney injury in hospitalized older adults**. *BMC Med Inform Decis Mak* 2016, **16**:39.
147. Koyner JL, Adhikari R, Edelson DP, Churpek MM: **Development of a Multicenter Ward-Based AKI Prediction Model**. *Clin J Am Soc Nephrol* 2016, **11**(11):1935-1943.
148. Koyner JL, Carey KA, Edelson DP, Churpek MM: **The Development of a Machine Learning Inpatient Acute Kidney Injury Prediction Model**. *Crit Care Med* 2018, **46**(7):1070-1077.
149. Latus J, Schwab M, Tacconelli E, Pieper FM, Wegener D, Rettenmaier B, Schwab A, Hoffmann L, Dippon J, Muller S *et al*: **Acute kidney injury and tools for risk-stratification in 456 patients with hantavirus-induced nephropathia epidemica**. *Nephrol Dial Transplant* 2015, **30**(2):245-251.
150. Li Y, Chen X, Shen Z, Wang Y, Hu J, Zhang Y, Xu J, Ding X: **Prediction models for acute kidney injury in patients with gastrointestinal cancers: a real-world study based on Bayesian networks**. *Ren Fail* 2020, **42**(1):869-876.
151. Li Y, Chen X, Wang Y, Hu J, Shen Z, Ding X: **Application of group LASSO regression based Bayesian networks in risk factors exploration and disease prediction for acute kidney injury in hospitalized patients with hematologic malignancies**. *BMC Nephrol* 2020, **21**(1):162.
152. Maiwall R, Sarin SK, Kumar S, Jain P, Kumar G, Bhadoria AS, Moreau R, Kedarisetty CK, Abbas Z, Amarapurkar D *et al*: **Development of predisposition, injury, response, organ failure model for predicting acute kidney injury in acute on chronic liver failure**. *Liver Int* 2017, **37**(10):1497-1507.
153. Martinez DA, Levin SR, Klein EY, Parikh CR, Menez S, Taylor RA, Hinson JS: **Early Prediction of Acute Kidney Injury in the Emergency Department With Machine-Learning Methods Applied to Electronic Health Record Data**. *Ann Emerg Med* 2020, **76**(4):501-514.
154. Matheny ME, Miller RA, Ikizler TA, Waitman LR, Denny JC, Schildcrout JS, Dittus RS, Peterson JF: **Development of inpatient risk stratification models of acute kidney injury for use in electronic health records**. *Med Decis Making* 2010, **30**(6):639-650.
155. Patidar KR, Xu C, Shamseddeen H, Cheng YW, Ghabril MS, Mukthinuthalapati V, Fricker ZP, Akinyeye S, Nephew LD, Desai AP *et al*: **Development and Validation of a Model to Predict Acute Kidney Injury in Hospitalized Patients With Cirrhosis**. *Clin Transl Gastroenterol* 2019, **10**(9):e00075.
156. Porter CJ, Moppett IK, Juurlink I, Nightingale J, Moran CG, Devonald MA: **Acute and chronic kidney disease in elderly patients with hip fracture: prevalence, risk factors and outcome with development and validation of a risk prediction model for acute kidney injury**. *BMC Nephrol* 2017, **18**(1):20.

© 2023 Feng Y et al. *JAMA Network Open.*

157. Qu C, Gao L, Yu XQ, Wei M, Fang GQ, He J, Cao LX, Ke L, Tong ZH, Li WQ: **Machine Learning Models of Acute Kidney Injury Prediction in Acute Pancreatitis Patients**. *Gastroenterol Res Pract* 2020, **2020**:3431290.

158. Simonov M, Ugwuowo U, Moreira E, Yamamoto Y, Biswas A, Martin M, Testani J, Wilson FP: **A simple real-time model for predicting acute kidney injury in hospitalized patients in the US: A descriptive modeling study**. *PLoS Med* 2019, **16**(7):e1002861.

159. Song X, Yu ASL, Kellum JA, Waitman LR, Matheny ME, Simpson SQ, Hu Y, Liu M: **Cross-site transportability of an explainable artificial intelligence model for acute kidney injury prediction**. *Nat Commun* 2020, **11**(1):5668.

160. Sujan R, Cruz-Lemini M, Altamirano J, Simonetto DA, Maiwall R, Axley P, Richardson T, Desai V, Cabezas J, Vargas V *et al*: **A Validated Score Predicts Acute Kidney Injury and Survival in Patients With Alcoholic Hepatitis**. *Liver Transpl* 2018, **24**(12):1655-1664.

161. Wu L, Hu Y, Liu X, Zhang X, Chen W, Yu ASL, Kellum JA, Waitman LR, Liu M: **Feature Ranking in Predictive Models for Hospital-Acquired Acute Kidney Injury**. *Sci Rep* 2018, **8**(1):17298.

162. Zhou J, Bai Y, Wang X, Yang J, Fu P, Cai D, Yang L: **A simple risk score for prediction of sepsis associated-acute kidney injury in critically ill patients**. *J Nephrol* 2019, **32**(6):947-956.

163. Burckhardt P, Nagin D, Vijayasarathy VPR, Padman R: **Multi-Trajectory Modeling to Predict Acute Kidney Injury in Chronic Kidney Disease Patients**. *AMIA Annu Symp Proc* 2018, **2018**:1196-1205.

© 2023 Feng Y et al. *JAMA Network Open.*
