# Supplementary material for: Characterization of Risk Prediction Models for Acute Kidney Injury: A Systematic Review and Meta-analysis
Source: JAMA Netw Open. 2023 May 15;6(5):e2313359. doi: 10.1001/jamanetworkopen.2023.13359 (PMC12011341; doi:10.1001/jamanetworkopen.2023.13359)
Supplement: Supplement 2. — Data Sharing Statement [file jamanetwopen-e2313359-s002.pdf]

Data Sharing Statement

Feng. Characterization of Risk Prediction Models for Acute Kidney Injury. *JAMA Netw Open*. Published May 15, 2023. doi:10.1001/jamanetworkopen.2023.13359

Data
Data available: Yes
Data types: Data (not involving human participants)
How to access data: The data used and generated in this study and analytic codes can be obtained from corresponding author on reasonable request.
When available: With publication

Supporting Documents
Document types: None

Additional Information
Who can access the data: The data used and generated in this study and analytic codes can be obtained from corresponding author on reasonable request.
Types of analyses: for any purpose
Mechanisms of data availability: The data used and generated in this study and analytic codes can be obtained from corresponding author on reasonable request.
